# Tcf15 promotes an open nucleolar chromatin state to safeguard ribosome biogenesis and genome stability in mouse embryonic stem cells

**DOI:** 10.1371/journal.pbio.3003954

**Published:** 2026-08-25

**Authors:** Yu-ping Dong, Yi-Min Chen, Min Tang, Hu Zhou, Lin Wang, Ping Zheng

**Affiliations:** 1 State Key Laboratory of Genetic Evolution and Animal Models, Key Laboratory of Animal Models and Human Disease Mechanisms of Yunnan Province, Kunming Institute of Zoology, Chinese Academy of Sciences, Kunming, Yunnan, China; 2 University of Chinese Academy of Sciences, Beijing, China; 3 Department of Analytical Chemistry and CAS Key Laboratory of Receptor Research, Shanghai Institute of Materia Medica, Chinese Academy of Sciences, Shanghai, China; 4 KIZ/CUHK Joint Laboratory of Bioresources and Molecular Research in Common Diseases, Kunming Institute of Zoology, Chinese Academy of Sciences, Kunming, Yunnan, China; Max F Perutz Laboratories Center of Molecular Biology, AUSTRIA

## Abstract

Embryonic stem cells (ESCs) exhibit a hyperactive chromatin state at ribosomal RNA (rRNA) genes, which not only plays roles in active rRNA synthesis and ribosome biogenesis (RiBi), but also links to genome architecture. However, how this active chromatin state is maintained in ESCs remains poorly understood. Here, we identify Tcf15, a mouse ESC-specific factor, as a novel regulator of ribosomal DNA (rDNA) chromatin state. Tcf15 localizes to the nucleolus, binds the coding region of rRNA genes, and independently recruits epigenetic modifiers—either Tet2 or Rbbp5 (a core component of H3K4 methyltransferases)—to promote an active chromatin configuration. Depletion of Tcf15 increases DNA methylation and H3K27me3 levels at rDNA. Intriguingly, the Tcf15-Rbbp5 axis ensures precursor rRNA transcription and RiBi, whereas the Tcf15-Tet2 axis is not involved in rRNA synthesis. Ribosome profiling further revealed compromised translation of a subset of mRNAs involved in DNA replication, damage response, and repair. Consequently, Tcf15- or Rbbp5-deficient ESCs exhibit severe genomic instability. Our findings add a new regulatory layer of chromatin state in rDNA of stem cells, and reveal a previously unrecognized phenotypic consequence of defective RiBi in ESCs.

## Abstract 2

Embryonic stem cells maintain an unusually active rDNA chromatin state to support their maintenance. This study shows that the transcription factor Tcf15 preserves this open chromatin state by recruiting the epigenetic regulators Tet2 or Rbbp5 to rRNA genes to sustain their transcription.

## Introduction

Ribosome biogenesis (RiBi) is closely associated with cellular states [1]. It has been well documented that stem cells, including embryonic stem cells (ESCs) and adult stem cells, possess a high rate of RiBi when compared to their differentiated progeny. Many stem cell types are sensitive to the loss of function of RiBi components, and stem cell maintenance heavily relies on the up-regulated RiBi. RiBi consists of many carefully orchestrated events including RiBi component synthesis, processing, transportation, and assembly. RiBi starts with RNA polymerase I (Pol I)-dependent transcription of ribosomal RNA (rRNA) genes to produce 47S precursor rRNA (pre-rRNA). The nucleolus is the largest compartment in the nucleus, where the rRNAs are transcribed and processed. In mouse and human genomes, ~ 200–300 copies of tandem rRNA genes from up to six chromosomes are clustered. The rRNA genes are transcribed to generate pre-rRNAs, which are then processed and cleaved to produce mature 28S, 18S, and 5.8S rRNAs, essential for downstream ribosome assembly. A high rate of rRNA synthesis is therefore a general phenomenon in stem cells [2].

ESCs are derived from the inner cell mass of a blastocyst and characterized by pluripotency and rapid self-renewal and proliferation. The up-regulated RiBi in ESCs is achieved by multiple mechanisms [1]. For instance, ESCs establish a hyperactive chromatin state at ribosomal DNA loci (rDNA) to allow for active rRNA transcription [3]. The hyperactive chromatin state at rDNA is characterized by DNA hypomethylation as well as low levels of repressive histone modifications, including H3K9me2/3 and H3K27me3. In addition, ESCs up-regulate a number of protein factors that associate with either RNA Pol I or Pol II complexes to promote the transcription of pre-rRNA and ribosomal protein (r-protein) RNAs, or to function in pre-rRNA processing and maturation [1]. Examples of such proteins include Myc [4], HTATSF1 [5], Fibrillarin (FBL) [6], UBR5 [7], RSL24D1 [8], and DDX10 [9]. Myc can localize in the nucleolus of ESCs and functions as a transcription factor to stimulate the transcription of rRNA, various r-proteins, and RiBi components [4]. HTATSF1 plays roles in rRNA and r-protein mRNA transcription and splicing [5]. FBL is a nucleolar methyltransferase responsible for the 2′-O-methylation of rRNAs essential for rRNA processing [6]. E3 ubiquitin ligase UBR5 participates in rRNA maturation [7]. RSL24D1 is associated with nuclear pre-ribosomes and is required for the biogenesis of 60S subunits [8]. RNA-binding protein DDX10 localizes in the nucleolar dense fibrillar component and the granular component and is required for 18S rRNA maturation [9].

Because RiBi starts with Pol I-dependent transcription of rRNA genes to produce 47S pre-rRNA, the establishment of a hyperactive chromatin state at rDNA is critical for ESCs. Of note, the open chromatin state in the nucleolus of ESCs not only allows active rRNA transcription and RiBi, but also plays an active role in remodeling genome organization and architecture outside the nucleolus [3]. How ESCs establish the hyperactive chromatin state at rDNA is not fully understood. Two mechanisms have been proposed. Savic and colleagues reported that the lack of DNA methylation in rRNA genes of mouse ESCs was attributed to the blockage of promoter-associated RNA (pRNA)–nucleolar remodeling complex (NoRC)--mediated rRNA gene silencing pathway [10]. In differentiated cells, rRNA genes are silenced by NoRC composed of TIP5 (TTF-1-interacting protein 5) and SNF2H. NoRC interacts with DNA methyltransferases, and once recruited to the promoter of rRNA genes, these enzymes methylate the promoter regions. The recruitment of NoRC to rRNA gene promoters is under the control of a lncRNA called promoter-associated RNA (pRNA), which regulates the association of TIP5 with TTF-1 located at the promoter of rRNA genes. Mature pRNA is produced through processing of intergenic spacer (IGS)-rRNA by RNA helicase DHX9. In ESCs, the IGS-rRNA processing is impaired, disrupting the mature pRNA production and NoRC recruitment to rRNA genes. A recent study revealed a mechanism underpinning the suppression of H3K27me3 modification at rRNA genes of ESCs [11]. They identified DEAD-box RNA helicase 18 (DDX18), which binds and sequesters PRC2 (polycomb repressive complex 2) in the outer layer of the nucleolus, thereby preventing the deposition of repressive H3K27me3 mark onto rRNA genes.

Transcription factor Tcf15 is highly expressed in mouse ESCs and the inner cell mass (ICM) of the pre-implantation blastocyst when compared to epiblast stem cells or differentiated somatic cells [12]. *Tcf15* has been identified as an ESC identity gene whose transcription is under the control of a super-enhancer [13]. In addition, Tcf15 expression marks the most primitive subset of true multipotent hematopoietic stem cells (HSCs), and is required and sufficient to drive long-term self-renewal of HSCs [14]. However, the functions of Tcf15 in ESCs and HSCs are poorly explored. A previous study reported that *Tcf15* expression was elevated in mouse ESCs primed for differentiation, and Tcf15 played pro-differentiation roles [12]. Using mouse ESCs as a model, we reveal that Tcf15 is a novel regulator of rDNA chromatin state by interacting with Tet2 or Rbbp5 to ensure the hyperactive chromatin structure at rDNA. Furthermore, the Tcf15-Rbbp5 axis participates in RiBi, thereby playing a unique role in maintaining ESC genomic stability.

## Results

### Tcf15 localizes in the nucleolus and binds to rRNA gene coding regions in mouse ESCs

Previously, we performed CRISPR/Cas9 screening to identify the regulators of genomic stability in mouse ESCs. Among the candidates, we found the transcription factor *Tcf15*, an ESC identity gene, as a regulator. ESC identity genes often play critical roles in determining/maintaining cellular properties [15], and their expression is driven by super-enhancers [13]. Although a previous study reported that Tcf15 was essential for ESC differentiation [12], the importance of Tcf15 in ESCs remains poorly characterized. The identification of *Tcf15* by CRISPR screening suggested its novel functions in ESCs, encouraging us to further explore them.

**Fig 1:**
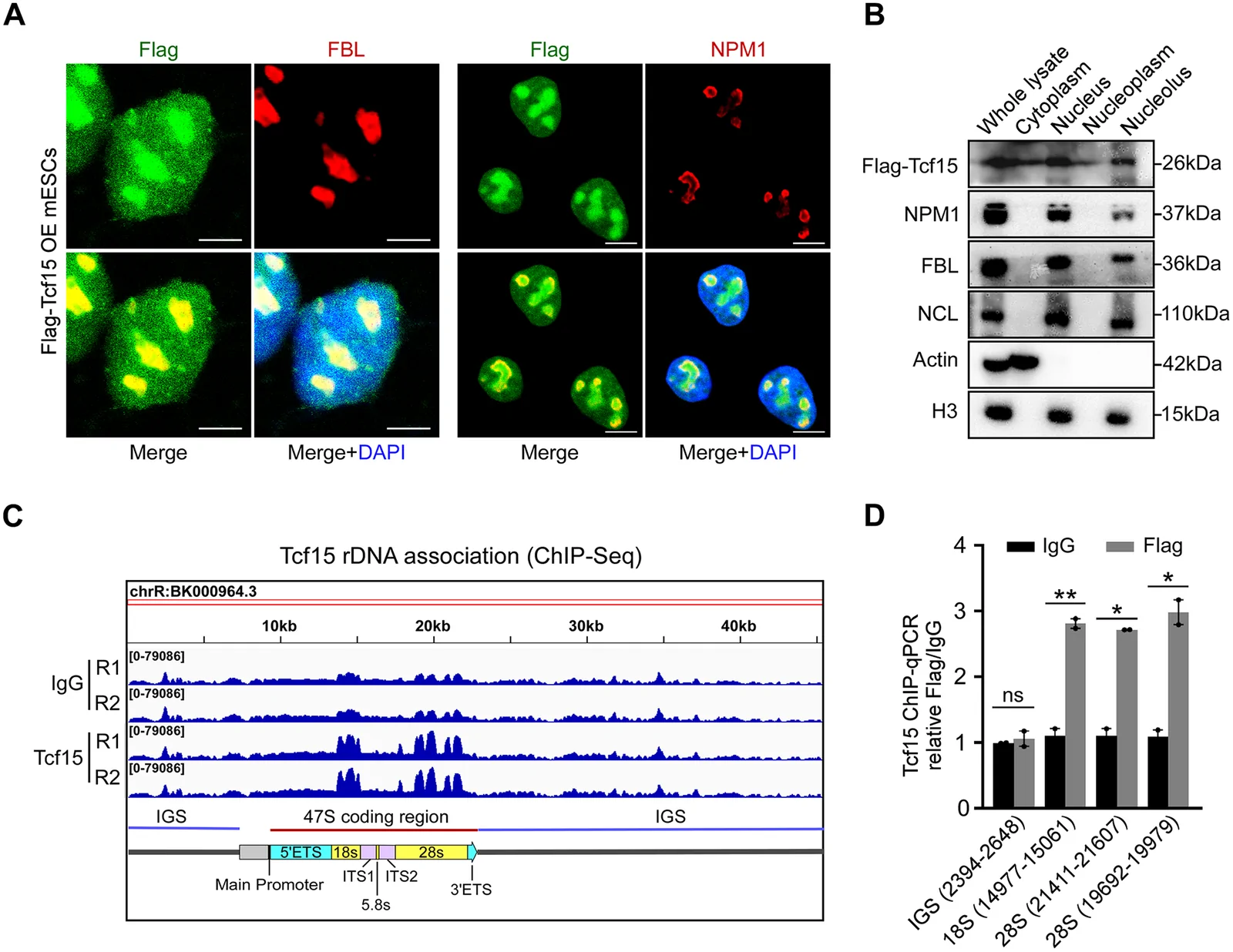
Tcf15 localizes in the nucleolus and binds to the coding regions of rRNA genes in ESCs. (**A**) Immunofluorescence staining revealed that Flag-tagged Tcf15 (Flag-Tcf15, green) co-localized with nucleolar markers FBL (red) and NPM1 (red) in ESCs. Scale bar, 5 μm. (**B**) Subcellular fractionation and immunoblotting showed that Tcf15 was detected in the nucleolus. (**C**) Genomic track from ChIP-seq data showed Tcf15 bound to 18S and 28S coding regions of rRNA genes. (**D**) ChIP-qPCR validated the binding of Tcf15 to the 18S and 28S regions of rRNA genes. Experiments in (**A**, **B**) were repeated three times using independent biological samples, and similar results were obtained. Data in (**D**) were shown as mean ± SEM (*n* = 2). Two-tailed Student *t* test, *P < 0.05, **P < 0.01; ns, not significant. The data underlying this figure can be found in S10 Table.

To this end, we first investigated the subcellular localization of Tcf15. Due to the lack of a reliable antibody recognizing endogenous Tcf15, 3×Flag-tagged Tcf15 was forcibly expressed in wild-type mouse ESCs with a C57BL/6J genetic background (Flag-Tcf15 OE cells) (S1A Fig) and maintained in conventional Knockout Serum Replacement (KSR)/LIF culture medium. Intriguingly, immunostaining with Flag antibody detected intense immunoreactive foci in nuclear areas with faint DAPI staining, suggestive of nucleolar localization. We then counterstained Flag-Tcf15 with different nucleolus markers, including FBL and Nucleophosmin 1 (NPM1), which marks the dense fibrillar component and the granular component of the nucleolus, respectively [16]. Interestingly, co-immunostaining showed that Tcf15 foci were predominantly localized in the nucleolus (Fig 1A). To exclude the artifact of protein overexpression, we tried to insert a Flag tag at the C-terminus of Tcf15 by a knock-in strategy. However, despite many attempts, we failed to insert the tag, probably owing to the DNA sequence issue. Alternatively, we knocked down (KD) Tcf15 via two independent short hairpin RNAs (shRNAs) (S1B Fig), and re-introduced Flag-*Tcf15* into KD ESCs (KD-rescue) (S1C and S1D Fig). After confirming that the mRNA level of *Tcf15* in KD-rescue ESCs was comparable to that in wild-type (WT) cells (S1E Fig), we re-examined the subcellular localization of Tcf15 in KD-rescue ESCs. Consistently, Tcf15 was reproducibly detected in the nucleolus of KD-rescue ESCs (S1F Fig). When ESCs were maintained in the 2i/LIF condition, which favors the ground state of naïve pluripotency, Tcf15 was consistently detected in the nucleolus (S1G Fig). Moreover, 3×Flag-tagged Tcf15 was also detected in the nucleolus in R1 mouse ESCs with a different genetic background (S1H Fig). These observations suggest that the localization of Tcf15 in the nucleolus is a general characteristic of ESCs. We therefore used ESCs with a C57BL/6J background, maintained in the conventional KSR/LIF condition, for all the downstream studies.

To further define the subcellular distribution of Tcf15, we performed cellular fractionation and purified nucleoli according to the protocol described previously [17,18]. Immunoblotting analysis detected Tcf15 in the cytoplasm and nucleus. In the nucleus, Tcf15 was enriched in the nucleolus compared with the nucleoplasm (Fig 1B). As a transcription factor, Tcf15 may bind to DNA. We performed chromatin immunoprecipitation sequencing (ChIP-seq) on Flag-Tcf15 OE ESCs to map the chromatin binding sites of Tcf15. Strong Tcf15 binding peaks were detected at the 18S and 28S rRNA gene loci (Fig 1C). The binding of Tcf15 at these loci was validated by quantitative PCR analysis of ChIP samples (Fig 1D). To systematically explore Tcf15 binding beyond rDNA loci, we annotated all identified ChIP-seq peaks across the genome. Genomic distribution analysis demonstrated that only 68 additional binding peaks were detected in genomic regions outside rDNA (S1 Table). These peaks were predominantly enriched at the distal regulatory regions of protein-coding genes (S1I Fig and S1 Table). Collectively, these data suggest that Tcf15 predominantly functions as a dedicated rDNA regulator in ESCs, with only a small fraction of its chromatin binding events occurring at other genomic loci.

### Tcf15 suppresses repressive epigenetic modifications of rRNA genes in ESCs

**Fig 2:**
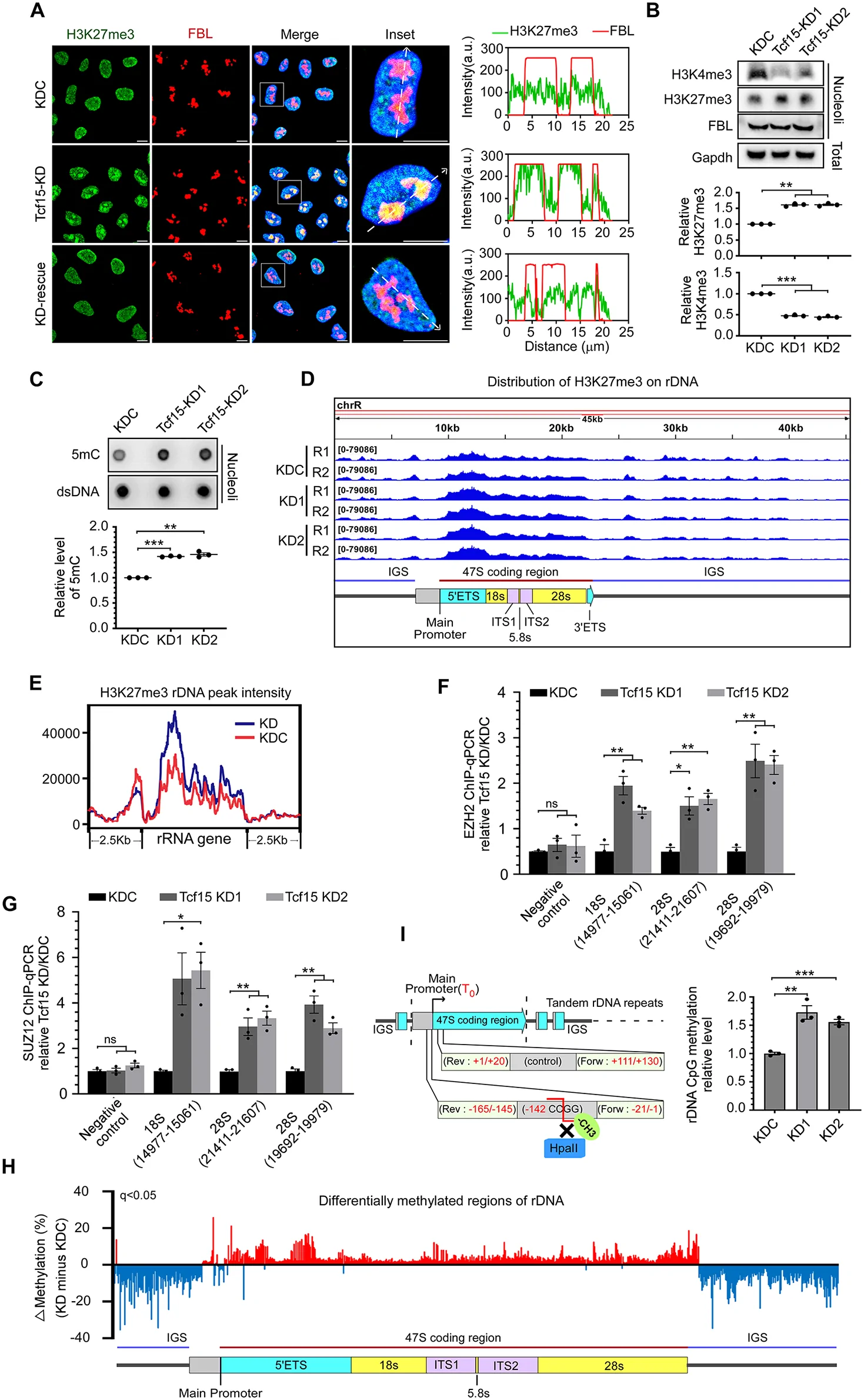
Tcf15 suppresses H3K27me3 modification and DNA methylation in rRNA genes of ESCs. (**A**) Immunofluorescence staining revealed that Tcf15 KD increased the H3K27me3 level in the nucleolus. The defect was rescued by re-expression of Tcf15 in KD cells (KD-rescue). Fluorescence intensity analyses of H3K27me3 and FBL along the line-labeled areas were shown (right panel). Scale bar, 10 μm. (**B**) Nucleoli were purified. Western blot analyzed the changes of H3K4me3 and H3K27me3 levels in nucleoli of KDC, Tcf15-KD1, and Tcf15-KD2 ESCs. FBL was used as a nucleolus loading control (upper panel). The changes were quantified in the lower panel. (**C**) Nucleoli were purified. Dot blot analysis of nucleolar DNA showed that Tcf15 KD ESCs had higher DNA m5C methylation levels than KDC cells. DNA was used as a loading control. The fold changes of methylation level were quantified in the lower panel. (**D**) Profiles of H3K27me3 modification on rDNA regions (chrR) in KDC and Tcf15-KD ESCs. (**E**) Peak intensity profiles of H3K27me3 at rRNA genes in KDC and Tcf15-KD ESCs. (**F**) ChIP-qPCR analysis showing increased binding of PRC2 core subunit EZH2 at rDNA regions in Tcf15-KD ESCs. (**G**) ChIP-qPCR analysis showing increased binding of PRC2 core subunit SUZ12 at rDNA regions in Tcf15-KD ESCs. (**H**) rDNA regions showing differential DNA methylation levels between KDC and Tcf15-KD ESCs. Note that red peaks indicated regions with increased DNA methylation in Tcf15-KD ESCs. (**I**) CpG methylation levels of rDNA in KDC and Tcf15-KD ESCs were validated using qPCR (right panel). Left panel was the schematic showing the working principle of PCR. Experiments in (**A**, **B**, **C**, **F**, **G**, and **I**) were repeated three times from independent biological samples, and similar results were obtained. Two replicates were performed in (**D** and **H**). Data were shown as mean ± SEM. Two-tailed Student *t* test, **P < 0.01, ***P < 0.001, ns, not significant. The data underlying this figure can be found in S10 Table.

Distinct from lineage-committed cells, the nucleoli of ESCs, no matter whether cells are cultured in the 2i/LIF condition (the naïve ground state) or in conventional KSR/LIF condition, contain unique epigenetic features characterized by DNA hypomethylation and lack of heterochromatic marks (H3K9me3/2 or H3K27me3) at rRNA genes [3]. Given that Tcf15 binds to rDNA regions, we sought to determine whether Tcf15 regulates these epigenetic features. To this end, we investigated whether Tcf15 knockdown alters the abundance of H3K27me3, H3K4me3, H3K9me3, and CpG DNA methylation within the nucleolus. Initial analysis by immunofluorescence staining showed that H3K27me3 (Fig 2A), H3K4me3 (S2A Fig), and 5-methylcytosine (m5C) (S2B Fig) displayed visible changes upon Tcf15 KD, whereas H3K9me3 signal intensity was not disturbed (S2C Fig). Re-expression of Tcf15 in KD cells rescued the changes (Figs 2A, S2A, and S2B). Immunoblotting of whole-cell lysates and dot blotting of nuclear DNA confirmed the genome-wide changes in H3K27me3 (S2D Fig), H3K4me3 (S2D Fig), and m5C (S2E Fig). Notably, the immunofluorescence staining intensity of H3K27me3 in the nucleolus showed a significant increase in KD cells, which was reversed by re-expression of Tcf15 (Fig 2A). To quantify the epigenetic changes in the nucleolus after Tcf15 KD, we purified nucleoli and repeated the immunoblotting and dot blotting analyses. Consistently, Tcf15 KD resulted in an increased abundance of H3K27me3 (Fig 2B) and m5C (Fig 2C), and a concomitant reduction in H3K4me3 (Fig 2B) in the nucleolus of ESCs. Further, we mapped the distribution profiles of H3K27me3 by Cleavage Under Targets and Tagmentation (CUT&Tag). The increased peak intensity of H3K27me3 was reproducibly detected in rRNA genes encoding the 47S rRNA precursor in KD1 and KD2 ESCs compared to KD control (KDC) (Fig 2D and 2E). Consistent with the elevated H3K27me3 levels at rDNA in Tcf15 KD ESCs, we observed PRC2 occupancy, as confirmed by ChIP‑qPCR showing significant enrichment of its core subunits EZH2 and SUZ12 at rDNA loci relative to controls (Fig 2F and 2G). Collectively, these data indicate that Tcf15-mediated H3K4me3 maintenance antagonizes PRC2 recruitment and activity at rDNA, and that Tcf15 loss relieves this inhibition, leading to ectopic PRC2 deposition and subsequent H3K27me3 accumulation. We also performed whole-genome bisulfite sequencing to map DNA methylation. DNA methylation at the promoter and coding regions is responsible for rRNA gene silencing [19]. Focusing on rDNA loci, we observed an increase in DNA methylation in the rRNA gene encoding regions after Tcf15 KD (Fig 2H). The methylation change was further quantified by qPCR following HpaII digestion of rDNA [10,20] (Fig 2I). Altogether, these results indicate that Tcf15 is required for the chromatin hyper-activation in the nucleolus of ESCs by repressing repressive epigenetic modifications.

To further confirm the direct role of Tcf15 in epigenetic regulations of rDNA loci, we attempted to insert the degron protein degradation cassette at the C-terminus of Tcf15. Again, we failed to achieve precise insertion, probably due to the DNA sequence issue. We therefore generated Tet-on-inducible shRNA KD ESCs. Doxycycline (Dox) treatment for 48 h caused a ~ 70% decrease in *Tcf15* mRNA expression (S3A Fig). Concordantly, Dox treatment for 48 h led to an increase in immunostaining intensity for nucleolar H3K27me3 (S3B Fig). Quantification of rDNA methylation by qPCR following HpaII digestion consistently showed the elevated methylation level in transient KD cells (S3C Fig). Thus, Tcf15 directly regulates the epigenetic modifications of rDNA loci.

### Tcf15 interacts with Tet2 or Rbbp5 in the nucleolus and recruits them onto rRNA genes to sustain a hyperactive chromatin architecture at nucleoli

**Fig 3:**
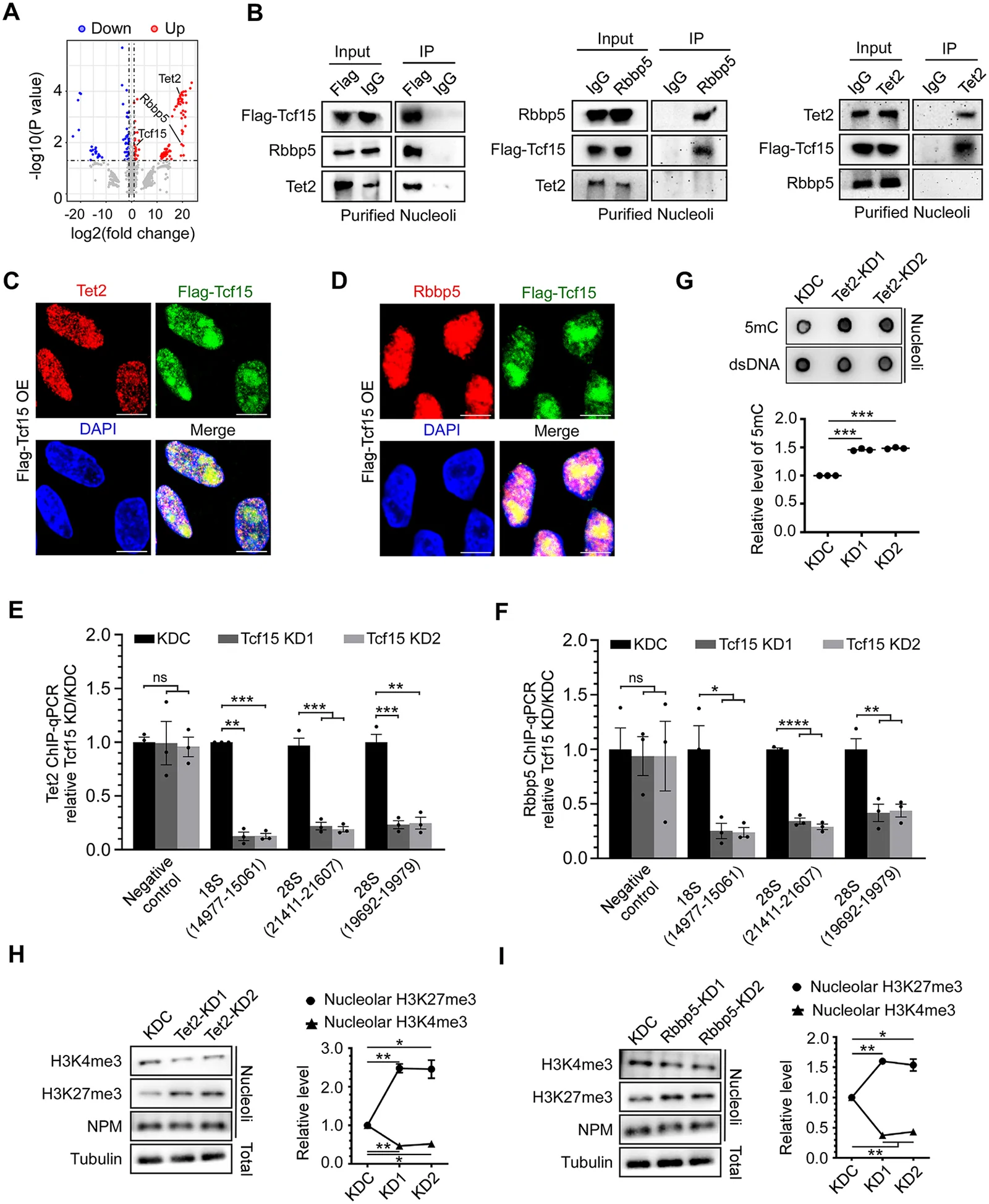
Tcf15 exerts its regulatory effects on nucleolar chromatin architecture via its physical interactions with Tet2 and Rbbp5 within the nucleolus. (**A**) Volcano plot analysis of Tcf15 interacting proteins identified by immunoprecipitation followed by mass spectrometry. IgG served as a control. Red dots represent proteins enriched in Tcf15 immunoprecipitates. (**B**) Reciprocal immunoprecipitation followed by immunoblotting validated the interaction of Tcf15 with Tet2 or Rbbp5 in purified nucleoli. No physical association was detected between Tet2 and Rbbp5. (**C**) Immunofluorescence staining of Tet2 (red) and Tcf15 (green) revealed partial colocalization. Scale bar, 10 μm. (**D**) Immunofluorescence staining of Rbbp5 (red) and Tcf15 (green) revealed partial co-localization. Scale bar, 10 μm. (**E** and **F**) ChIP‑qPCR analysis revealed that Tcf15 knockdown significantly reduced the binding of Tet2 and Rbbp5 to the 18S and 28S rDNA regions compared with control (KDC) cells. (**G**) Dot blot analysis of nucleolar DNA showed that Tet2 KD ESCs had higher DNA m5C methylation levels than KDC cells. DNA was used as a loading control. The fold changes of methylation levels were quantified in the lower panel. (**H, I**) KD of Tet2 (**H**) or Rbbp5 (**I**) decreased the H3K4me3 level but increased the H3K27me3 modification in the nucleoli. The relative levels of H3K4me3 and H3K27me3 in the nucleoli were normalized to NPM. Experiments were repeated three times from independent biological samples, and similar results were obtained. Data were shown as mean ± SEM. Two-tailed Student *t* test, *P < 0.05, **P < 0.01, ***P < 0.001, ****P < 0.0001, ns, not significant. The data underlying this figure can be found in S10 Table.

To understand how Tcf15 regulates the epigenetic modifications of rRNA genes, we constructed the TurboID system [21] to identify the interaction proteins (S4A and S4B Fig). Mass spectrometry analysis identified a list of potential Tcf15 interaction proteins (Fig 3A and S2 Table). Among the top candidates, we found two epigenetic regulators, Tet2 and Rbbp5 (Fig 3A). Tet2 regulates DNA demethylation as well as H3K4 methylation [22–25]. Rbbp5 is one of the four core subunits (Wdr5, Rbbp5, Ash2l, and Dpy30, referred to as WRAD) of the SET1 family of histone methyltransferases (HMTs) complexes catalyzing H3K4 methylation [26]. Active and repressive chromatin marks display mutual antagonism interplay. H3K4me2/3 inhibits PRC2 activity and prevents H3K27me3 deposition. Removal of the active marks increases H3K27me3 modification and reduces gene expression [27]. Based on the epigenetic changes detected in Tcf15 KD ESCs, we proposed that Tcf15 might associate with HMTs and Tet2, and recruit them onto rRNA genes to regulate epigenetic modifications.

To test this hypothesis, we first verified the interaction between Tcf15 and both Tet2 and Rbbp5. Co‑immunoprecipitation (co‑IP) from whole‑cell lysates showed that Tcf15 pulled down Tet2 and Rbbp5, and reciprocal co‑IP confirmed that both proteins could precipitate Tcf15. Notably, no interaction was detected between Rbbp5 and Tet2 (S4C Fig). These results indicate that Tcf15 independently associates with Tet2 and Rbbp5, rather than forming a ternary complex. To investigate protein interactions within the nucleolus, we first isolated nucleoli and performed co‑IP using nucleolar extracts, which yielded results consistent with those from whole‑cell lysates (Fig 3B). In addition, immunofluorescence staining revealed co‑localization of Tcf15 with Tet2 and Rbbp5 in the nucleolus (Fig 3C and 3D). Collectively, these data indicate that Tcf15 independently associates with Tet2 and Rbbp5 in the nucleolus, forming two separate complexes rather than a single ternary assembly. To test whether Tcf15 directly recruits Tet2 and Rbbp5 to rDNA, we performed ChIP‑qPCR with anti‑Tet2 and anti‑Rbbp5 antibodies in control and Tcf15‑KD ESCs (two independent knockdown lines). Binding of both factors to the 18S and 28S rDNA regions was significantly reduced following Tcf15 knockdown relative to controls (Fig 3E and 3F). These results support a model in which Tcf15 acts as an essential upstream scaffold that recruits both epigenetic regulators to rDNA.

Tet2 is well known for its classical role in DNA demethylation outside the nucleolus [28]. To our knowledge, its involvement in the regulation of the hypomethylation state of rDNA in ESCs has never been reported. In ESCs, the hypomethylation status of the rRNA gene promoter is ensured by the blockage of IGS-rRNA processing and, subsequently, the lack of mature pRNA-NoRC-mediated DNA methylation. However, it seemed that DNA methylation remained active in the rRNA gene promoter and coding region. Thus, Tet2-mediated DNA demethylation is necessary to establish the hypomethylation state in rDNA of ESCs. Tet2 can also function in a non-canonical manner to sustain the H3K4me3 level by interacting with O-GlcNAc transferase (OGT) and increasing its enzymatic activity. OGT catalyzes the GlcNAcylation of H3K4 methyltransferase Setd1A to stimulate its activity [24,25].

To verify that Tet2 and Rbbp5 regulate the epigenetic modifications of rRNA genes, we knocked down Tet2 and Rbbp5 in ESCs (S4D and S4E Fig). Initial analyses by immunofluorescence staining and immunoblotting showed that the KD of Tet2 or Rbbp5 increased H3K27me3 and decreased H3K4me3 (S4F-S4I Fig). We then examined the effects on epigenetic modifications of rDNA loci. Tet2 KD resulted in increased DNA methylation and H3K27me3, as well as a concomitant decrease in H3K4me3, in the nucleolus (Fig 3G and 3H). Similarly, increase of H3K27me3 and decrease of H3K4me3 were detected in the nucleoli of Rbbp5 KD ESCs (Fig 3I). Collectively, these findings establish that Tcf15 independently recruits Tet2 and Rbbp5 to rDNA as two separate complexes, which then execute epigenetic programs to maintain the active chromatin state in the nucleoli of ESCs.

### The Tcf15-Rbbp5 complex safeguards active rRNA transcription, ribosome biogenesis, and nascent protein synthesis

The nucleolus has emerged as a multifunctional cellular compartment, wherein an open chromatin configuration underlies not only the canonical process of ribosome biogenesis but also non‑canonical roles, particularly the preservation of three‑dimensional genome architecture [3]. This functional versatility implies that distinct open chromatin domains within the nucleolus are likely partitioned for separate tasks. Given that Tcf15 independently recruits Tet2 and Rbbp5 to rDNA, we postulated that these two complexes define separate open chromatin domains with distinct functions.

**Fig 4:**
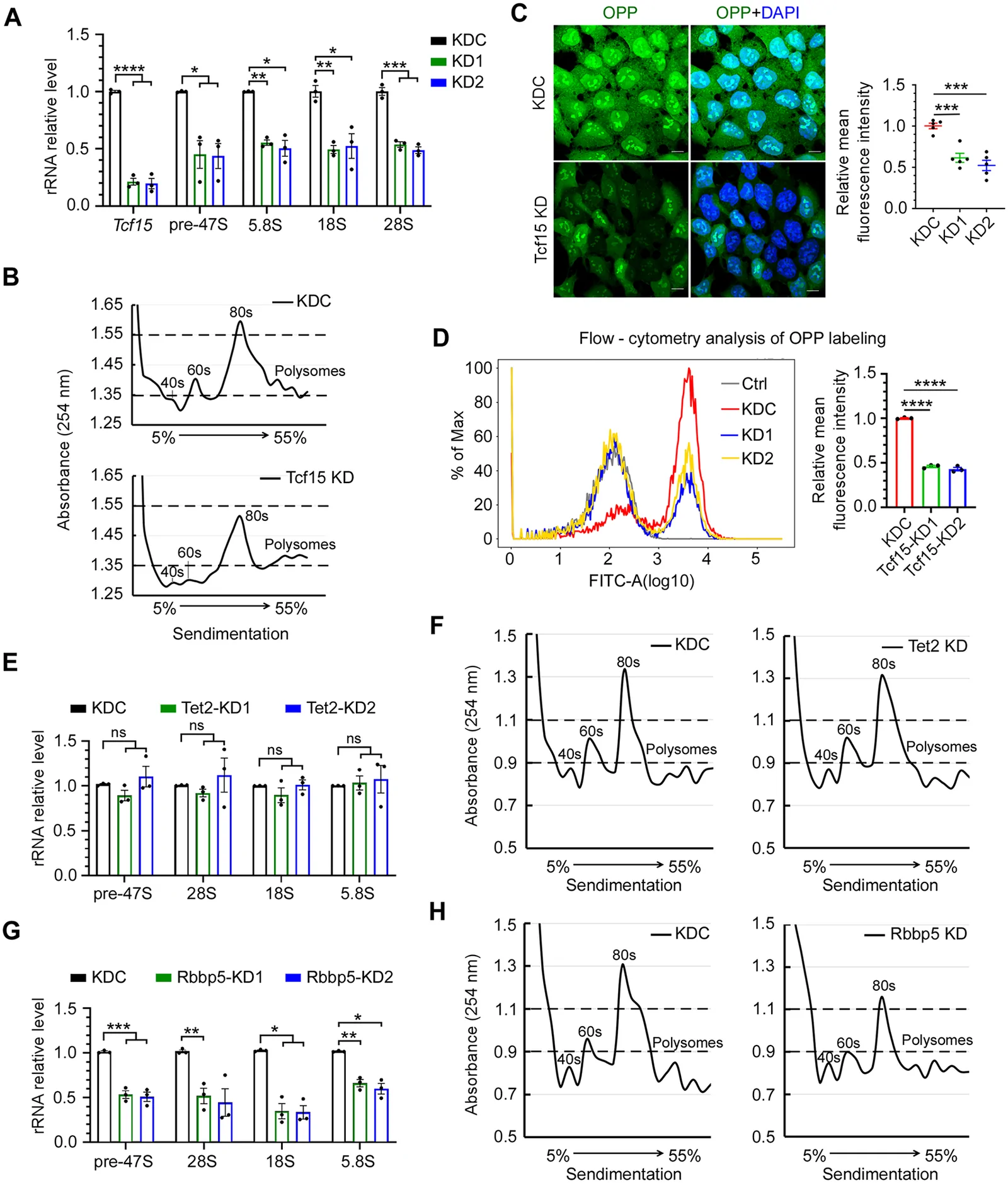
The Tcf15-Rbbp5 complex maintains active rRNA transcription, ribosome biogenesis, and nascent protein synthesis. (**A**) qRT-PCR showed the relative rRNA levels of pre-47S, mature 28S, 18S, and 5.8S rRNA species in Tcf15 KDC and KD ESCs. *Gapdh* was used as an internal control. (**B**) Sucrose gradient fractionation followed by the A_254_nm absorption analysis revealed the reduced amounts of ribosome subunits (40S and 60S), monosome (80S) and polysomes in Tcf15 KD ESCs compared to KDC. (**C**) Green fluorescence label of O-Propargyl-Puromycin (OPP) in Tcf15 KDC and KD ESCs. The relative fluorescence intensities were quantified in the right panel. (**D**) Flow cytometry analysis of OPP green fluorescence labeling in Tcf15 KDC and KD ESCs. Histograms are shown on a logarithmic scale with y-axis normalized to %Max. The relative fluorescence intensities are shown in the right panel. Equal starting cell numbers were loaded for each group during flow cytometry acquisition. The reduced cell events in Tcf15 KD samples reflect the decreased proportion of OPP-positive cells, consistent with the reduced nascent protein synthesis rate observed in (**C**). (**E**) qRT-PCR showed the relative levels of pre-47S, mature 28S, 18S, and 5.8S rRNA species in Tet2 KD and KDC ESCs. *Gapdh* was used as an internal control. (**F**) Sucrose gradient fractionation followed by A_254_nm absorption analysis revealed no significant change in the abundance of ribosome subunits (40S and 60S), monosome (80S) and polysomes in Tet2 KD ESCs versus KDC. (**G**) qRT-PCR showed the relative levels of pre-47S, mature 28S, 18S, and 5.8S rRNA species in Rbbp5 KD and KDC ESCs. *Gapdh* was used as an internal control. (**H**) Sucrose gradient fractionation followed by the A_254_nm absorption analysis revealed a significant change in the abundance of ribosome subunits (40S and 60S), monosome (80S) and polysomes in Rbbp5 KD ESCs versus KDC. Experiments were repeated three times from independent biological samples, and similar results were obtained. Data were shown as mean ± SEM. Two-tailed Student *t* test, *P < 0.05, **P < 0.01, ***P < 0.001, ****P < 0.0001, ns, not significant. The data underlying this figure can be found in S10 Table.

We began by characterizing the global consequences of Tcf15 loss on rRNA transcription and ribosome biogenesis, and quantified 47S pre‑rRNA levels via qRT‑PCR. KD of Tcf15 reduced the level of 47S pre-rRNA by approximately 50%. Concordantly, the transcripts of mature rRNAs including 5.8S, 18S and 28S were decreased in Tcf15 KD cells (Fig 4A). We next explored the effect of Tcf15 KD on ribosome biogenesis. The ribosome subunits (40S and 60S), monosome (80S) and polysomes were fractionated by sucrose gradient and the relative amounts were measured according to the absorbance reading at 254 nm. Concordant with the reduced rRNA transcription, the relative amounts of ribosome subunits (40S and 60S), monosome (80S) and polysomes were lower in Tcf15 KD ESCs than in KDC cells (Fig 4B). The compromised ribosome biogenesis in Tcf15 KD ESCs suggested that protein translation could be affected. Using the O-propargyl-puromycin (OPP) protein synthesis assay, which measures nascent protein translation rate [29], we found a significant decrease in overall nascent protein synthesis in Tcf15 KD ESCs (Fig 4C and 4D). Notably, using the highly sensitive OPP assay, we also detected nascent protein synthesis in the nucleolus. Although protein translation is conventionally thought to occur exclusively in the cytoplasm, accumulating evidence supports that translation can also occur within the nucleus and nucleolus [30]. This localized translation is proposed to enable the rapid, on-demand synthesis of proteins essential for nucleolar functions, allowing for immediate responses to nucleolar stress or fluctuating functional demands. Our detection of nucleolar translation in ESCs is consistent with this emerging model, reflecting the high metabolic and synthetic activity characteristic of the nucleolus in pluripotent stem cells.

To delineate the distinct roles of Tet2 and Rbbp5 in rRNA biogenesis, we performed individual knockdowns and assessed pre‑rRNA transcription and ribosome assembly. This approach revealed a marked functional asymmetry. Loss of Tet2 did not appreciably affect 47S pre‑rRNA abundance (Fig 4E) or overall ribosome biogenesis (Fig 4F). In contrast, Rbbp5 deficiency led to substantial reductions in both pre‑rRNA transcription (Fig 4G) and ribosome production (Fig 4H), mirroring the phenotype observed upon Tcf15 depletion. These observations indicate that Tcf15-Tet2 and Tcf15-Rbbp5 complexes govern distinct nucleolar chromatin states with non‑overlapping functions: the Rbbp5‑associated complex supports canonical rRNA transcription and ribosome biogenesis, whereas the Tet2‑associated complex appears dispensable for these processes. Whether Tet2‑directed rDNA demethylation contributes to chromatin architecture beyond rRNA synthesis warrants further investigation. Collectively, our findings establish that Tcf15 serves as a platform for two independent complexes that sustain functionally divergent nucleolar chromatin landscapes.

### Tcf15 deficiency selectively represses translation of a subset of ESC‑prevalent proteins

**Fig 5:**
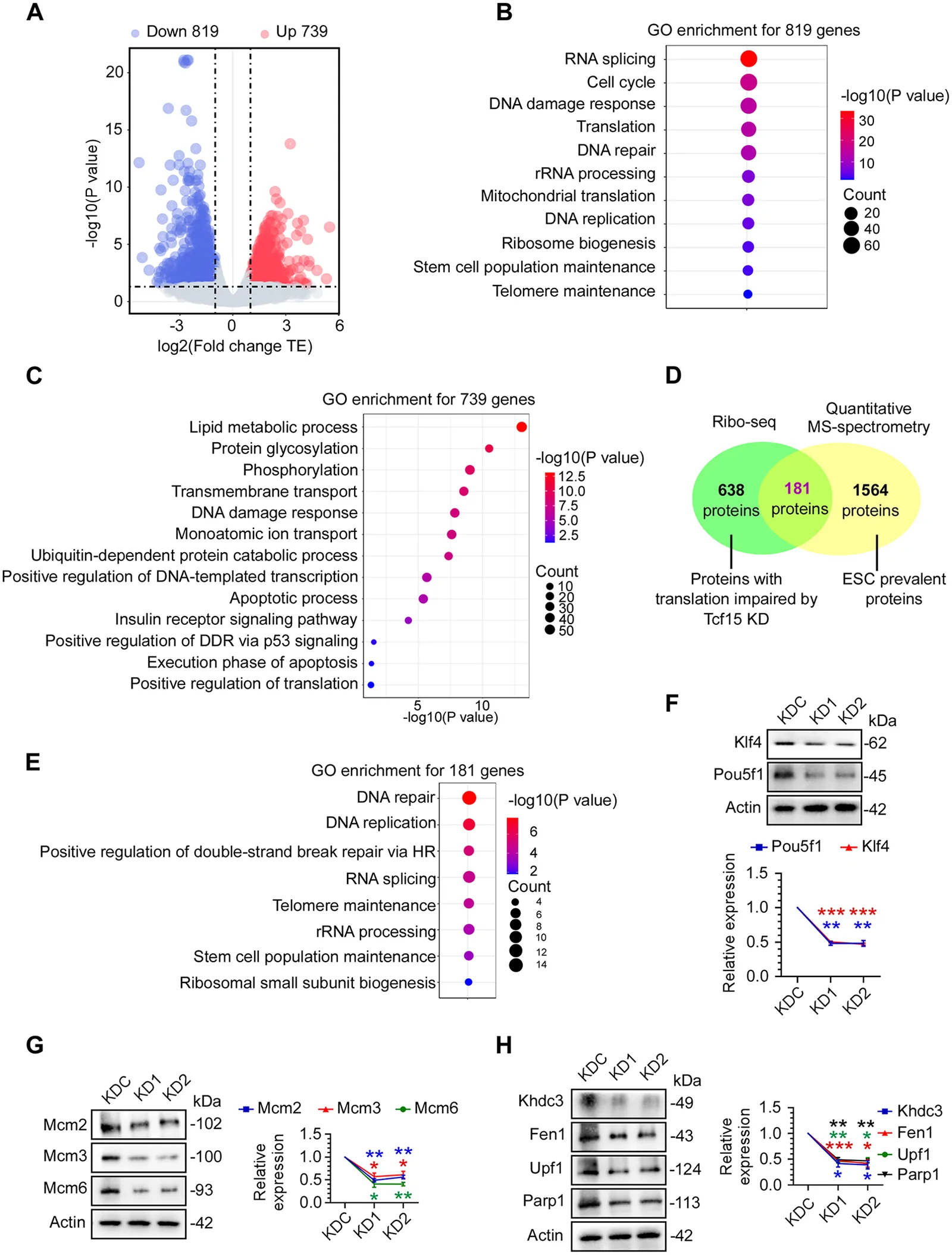
Loss of Tcf15 impairs translation of a subset of ESC‑prevalent proteins. (**A**) Volcano plot analysis of genes with translation affected by Tcf15 KD in ESCs. Blue dots represented genes showing decreased translation efficiency in Tcf15 KD ESCs, whereas red dots represented genes showing increased translation efficiency in Tcf15 KD cells. (**B**) Gene ontology (GO) enrichment analysis revealed the top biological processes enriched from genes with decreased translation efficiency in Tcf15 KD ESCs. (**C**) Gene ontology (GO) enrichment analysis revealed the biological processes enriched from genes with increased translation efficiency in Tcf15 KD ESCs. (**D**) Venn diagram showing overlap between the ESC-prevalent proteins and the proteins with translation decreased in Tcf15 KD ESCs. (**E**) GO enrichment of the overlapped 181 proteins in (**D**). (**F-H**) Western blot validated the decreased expression of several example proteins in Tcf15 KD ESCs when compared to KDC. Actin was used as a loading control, and the relative protein levels were shown in the lower panel. Experiments in (**F**), (**G**), (**H**) and MS-spectrometry were repeated three times and similar results were obtained. For Ribo-seq experiments, two biological replicates were performed. Data were shown as mean ± SEM. Two-tailed Student *t* test, *P < 0.05, **P < 0.01, ***P < 0.001. The data underlying this figure can be found in S10 Table.

RiBi perturbation often affects mRNA translation in a tissue‑ and cell‑specific manner [2]. To uncover translationally altered transcripts following Tcf15 KD, we performed paired RNA‑seq and ribosome footprinting (Ribo-seq) (S5A Fig). Surprisingly, transcriptional changes were minimal, with only 38 DEGs detected (S5B Fig and S3 Table). In contrast, integrative analysis identified 1,558 genes with unchanged mRNA levels but markedly altered translational output (|fold change| > 2): 819 were translationally down‑regulated and 739 up‑regulated (Fig 5A and S4 Table). Translationally repressed genes in KD cells are functionally enriched in RNA splicing, rRNA processing, cell cycle, translation, DNA replication, DNA repair, mitochondrial complex I assembly, and ATP synthesis (Fig 5B and S4 Table). By contrast, translationally activated transcripts are strongly enriched in p53 signaling and apoptotic pathways (Fig 5C and S5 Table), a typical ribosomal stress response. Indeed, defective ribosome biogenesis is known to elicit stress‑dependent translational reprogramming, favoring selective translation of stress‑response transcripts over global suppression [31,32].

To assess whether Tcf15 KD compromises the synthesis of ESC‑enriched proteins and perturbs stem cell traits, we quantified the ESC versus mouse embryonic fibroblasts (MEFs) proteome by mass spectrometry. We identified 1,745 proteins preferentially expressed in ESCs (fold change > 2, adjusted *p* < 0.05) (S5C Fig and S6 Table), which are enriched in pluripotency networks (e.g., Pou5f1, Sox2, Klf4, Sall4, Utf1, Esrrb), rRNA processing, DNA replication, DNA repair, and chromatin regulation (S5D Fig and S6 Table). Strikingly, 181 of the 819 translationally impaired proteins in Tcf15 KD cells are ESC‑enriched (Fig 5D), spanning DNA repair/genome stability (e.g., Fen1, Lig1, Parp1, Apex1, Khdc3, Sirt6, Xrcc6, Ung, Ddx18), DNA replication/cell proliferation (e.g., Pola2, Rfc3, Mcm3, Mcm6, Mcm2, Eras), rRNA processing/RiBi (e.g., Exosc5, Pes1, Wdr18, Nop2, Gar1, Rrp36, Wbp11, Ak6, Rrp15, Ncl), and pluripotency (e.g., Klf4, Pou5f1) (Fig 5E and S6 Table). Immunoblotting validated the reduced expression of selected proteins governing pluripotency (Klf4, Pou5f1) (Fig 5F), DNA replication (Mcm2, Mcm3, Mcm6) (Fig 5G), and DNA repair (Khdc3, Fen1, Upf1, Parp1) (Fig 5H) upon Tcf15 KD. Taken together, these data show that Tcf15 depletion selectively impairs translation of a subset of ESC‑prevalent proteins.

### Tcf15 KD compromises DNA replication and genome stability in ESCs

**Fig 6:**
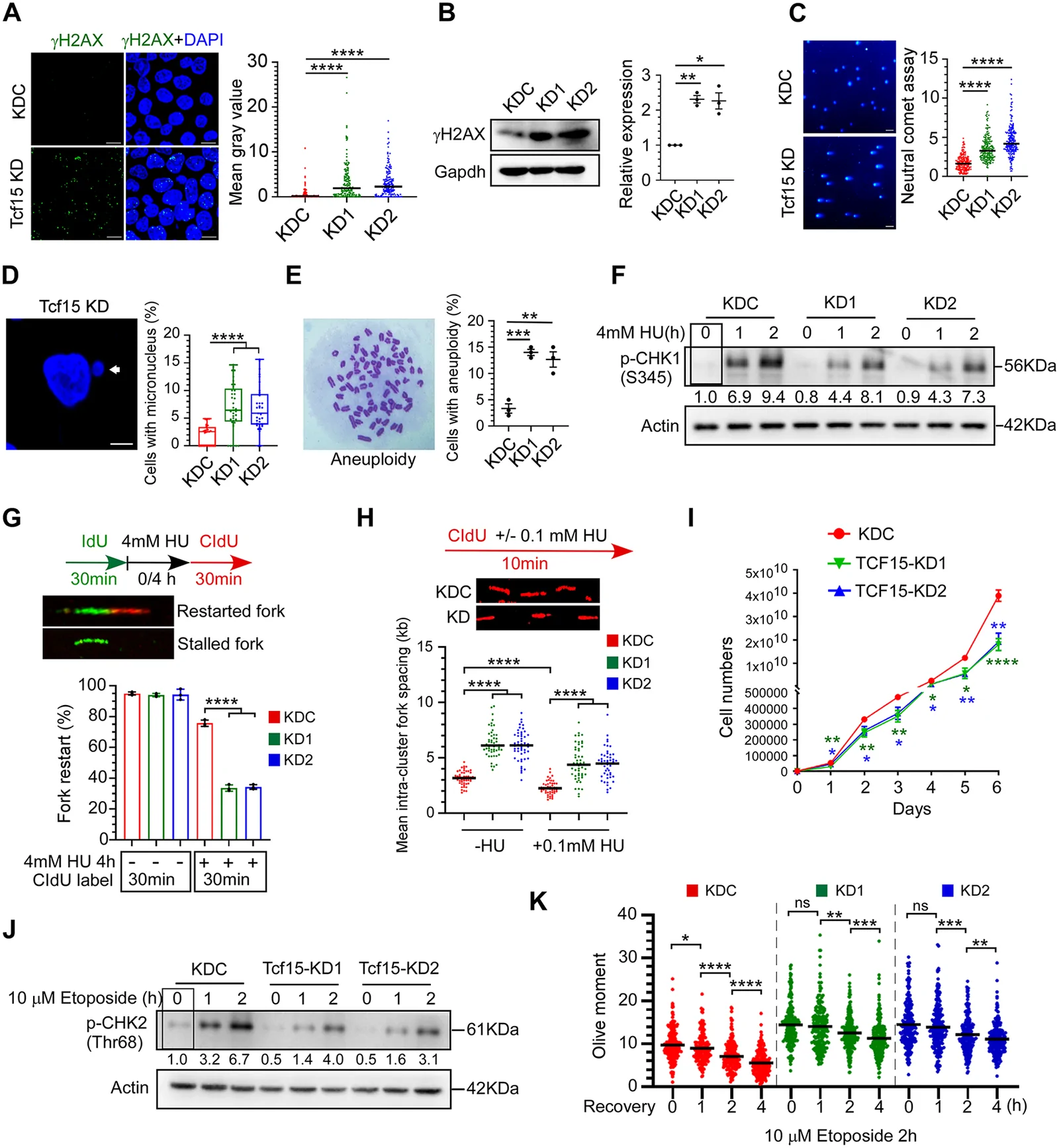
Tcf15 KD compromised DNA replication and genome stability in ESCs. (**A, B**) Immunofluorescence staining (A) and western blot (B) revealed that Tcf15 KD increased γH2AX levels. (**C**) Neutral comet assay showed the elevated DSBs in Tcf15 KD ESCs. (**D**) Analysis of cells with micronuclei in Tcf15 KDC and KD ESCs. (**E**) Analysis of aneuploidy in Tcf15 KDC and KD ESCs. (**F**) Western blot analysis of Chk1 activation (p-Chk1^S345^) in Tcf15 KDC and KD ESCs. Actin was used as a loading control, and the relative p-Chk1 levels were shown. (**G**) DNA fiber assay revealed that stalled replication fork restart was drastically compromised in Tcf15-KD ESCs under HU treatment. Representative images (top) and quantification (bottom) are shown. At least 200 fibers in each group were analyzed. (**H**) Replication fork initiation in KDC and Tcf15-KD ESCs was measured by DNA fiber assay. ESCs were treated with mild replication stress (0.1 mM HU treatment). At least 200 fibers in each group were analyzed. (**I**) Cell number counts in Tcf15 KD and KDC ESCs during continuous culture for 6 days. (**J**) Western blot analysis of Chk2 activation (p-Chk2^Thr68^) in Tcf15 KDC and KD ESCs. Actin was used as a loading control, and the relative p-Chk2 levels were shown. (**K**) ESCs were treated with 10 μM etoposide for 2 h and were recovered for 1, 2 and 4 h. Neutral comet assay showed the repair efficiency was compromised in Tcf15 KD ESCs. Experiments were repeated three times using independent biological samples, and similar results were obtained. Data were shown as mean ± SEM. Two-tailed Student *t* test, *P < 0.05, **P < 0.01, ***P < 0.001, ****P < 0.0001, ns, not significant. The data underlying this figure can be found in S10 Table.

Our findings establish that the Tcf15–Rbbp5 complex supports active rRNA transcription and ribosome biogenesis. Ribo‑seq further showed that Tcf15 knockdown selectively compromises translation of proteins governing DNA replication and repair (Fig 5H), including Parp1, a versatile DNA repair factor [33], and Khdc3 (also called Filia), an ESC‑specific regulator of replication stress and damage repair [34–36]. This translational defect in genome maintenance factors prompted us to examine whether Tcf15 loss triggers genomic instability. Indeed, Tcf15‑depleted ESCs displayed robust γH2AX accumulation (Fig 6A and 6B)—a hallmark of DSBs—and neutral comet assays confirmed elevated DSB levels relative to controls (Fig 6C). Moreover, Tcf15 KD cells showed increased micronuclei (Fig 6D) and aneuploidy (Fig 6E), further substantiating genomic instability.

DNA replication stress is a major endogenous source of DSBs [37]. We therefore examined whether Tcf15 KD impairs replication stress responses. Upon hydroxyurea (HU)‑induced replication stress, ATR‑Chk1 signaling—monitored by phosphorylation of Chk1 at Ser345—was compromised in Tcf15 KD ESCs (Fig 6F). Accordingly, DNA fiber assays revealed that recovery of stalled replication forks was severely impaired upon Tcf15 KD (Fig 6G). Notably, the mean fork spacing was significantly increased in Tcf15 KD ESCs under both unperturbed and stressed conditions (Fig 6H), indicating a reduced density of replication origins. This phenotype coincided with decreased expression of MCM proteins (Mcm2, Mcm3, Mcm6) [38], which form the core of the replicative helicase complex essential for replication origin licensing and fork progression. Reduced MCM levels result in a diminished pool of licensed origins, thereby decreasing origin density (Fig 6H). This origin scarcity impairs the cell’s ability to efficiently complete DNA replication, particularly under replication stress, leading to fork stalling, ATR-Chk1 signaling defects (Fig 6F), and ultimately DNA damage [39]. Concordantly, Tcf15 KD ESCs displayed a slower proliferation rate (Fig 6I). We also examined the ATM-Chk2 pathway, which is central to DSB repair. ATM-Chk2 activation (Fig 6J) and overall DSB repair capacity (Fig 6K) were both impaired in Tcf15 KD ESCs. Taken together, these data demonstrate that Tcf15‑dependent RiBi—driven by the Tcf15-Rbbp5 axis—is essential for maintaining genomic stability in ESCs through translational control of DNA replication and repair factors.

To further test this model, we asked whether Rbbp5 depletion—which disrupts RiBi—alone suffices to elicit DNA damage. Indeed, Rbbp5 KD increased γH2AX levels and DSBs, as detected by western blot, immunofluorescence and neutral comet assays (S6A-S6C Fig). These results reinforce the notion that the Tcf15‑Rbbp5 axis safeguards genome stability via RiBi‑dependent translational regulation.

Given that Tcf15 KD partially reduced Oct4 and Klf4 protein levels (Fig 5F), we asked whether pluripotency itself was affected. Tcf15 KD did not affect alkaline phosphatase (AP) staining (S6D Fig), expression of core pluripotency markers (S6E Fig), or embryoid body (EB) formation efficiency (S6F Fig), although Tcf15‑KD EBs were smaller (S6G Fig), consistent with the proliferation defect caused by impaired RiBi. Thus, the defects we observed arise directly from the role of Tcf15 in rDNA chromatin regulation and rRNA transcription, rather than from compromised pluripotency. Thus, Tcf15 depletion did not cause compromised pluripotency.

## Discussion

**Fig 7:**
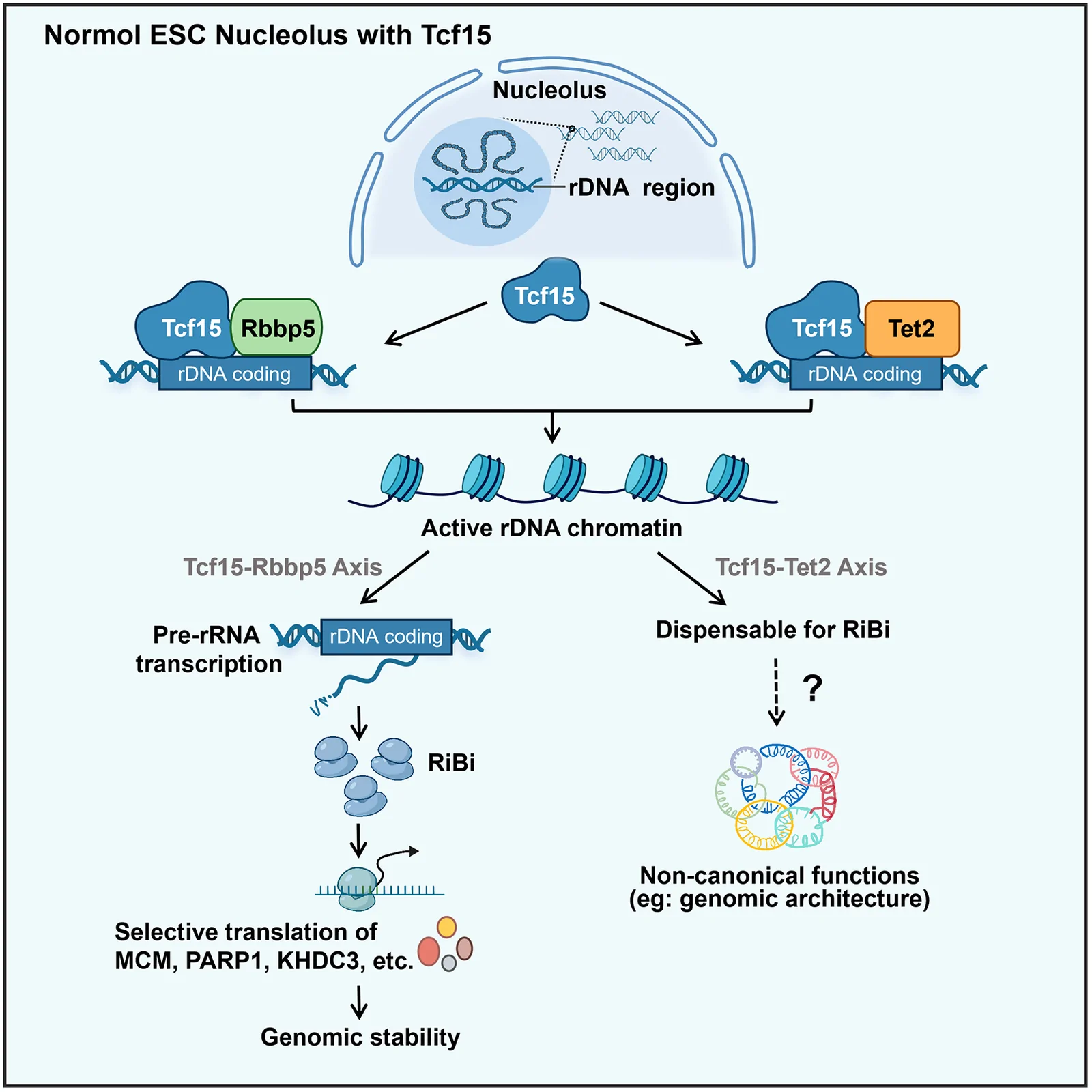
Schematic summary illustrating the proposed working model of Tcf15. In wild-type mouse embryonic stem cells, Tcf15 recruits Tet2 or Rbbp5 to activate rDNA chromatin. The Tcf15‑Rbbp5 and Tcf15‑Tet2 complexes exert distinct biological functions. Along the Tcf15‑Rbbp5 axis, Tcf15 facilitates rRNA transcription and ribosome biogenesis, as well as the selective translation of genes involved in DNA replication and DNA damage repair, thereby safeguarding genome stability. By contrast, the Tcf15‑Tet2 axis is not required for rRNA transcription and ribosome biogenesis but may participate in the organization of genome architecture.

Maintaining an active chromatin state at rDNA loci is essential for sustaining ESC identity. In this study, we identify Tcf15 as a novel ESC‑specific regulator of nucleolar chromatin structure. Tcf15 localizes to rDNA coding regions and recruits the epigenetic factors Tet2 and Rbbp5. Although Tet2 and Rbbp5 interact with Tcf15, both do not co‑immunoprecipitate with each other, suggesting that Tcf15 assembles them into physically distinct complexes: one containing Rbbp5 (a core subunit of the H3K4 methyltransferase complex) and the other containing Tet2 (a DNA dioxygenase). Through these parallel pathways, Tcf15 establishes a hyperactive chromatin state in the nucleolus. Functional dissection further reveals that the Tcf15‑Rbbp5 complex‑mediated active chromatin structure plays a canonical role in ensuring rRNA transcription and RiBi. In contrast, the Tcf15‑Tet2 complex‑mediated rDNA hypomethylation is dispensable for rRNA transcription and RiBi; whether it participates in non‑canonical functions, such as genome structure organization, warrants further investigation. Notably, we also found that Tet2 depletion induces DNA damage without altering rRNA transcription, indicating Tet2 preserves genome integrity via ribosome biogenesis-independent mechanisms. Finally, we show that the decline in RiBi resulting from depletion of the Tcf15‑Rbbp5 complex reduces the protein translation of a subset of ESC‑prevalent factors, impairing DNA replication and damage repair and ultimately leading to genomic instability (Fig 7).

An intriguing observation from our study is that Tcf15 binds preferentially to the coding regions (18S and 28S) rather than the promoter of rRNA genes. This unusual binding pattern raises the possibility that Tcf15 may facilitate transcription elongation or maintain an open chromatin configuration across the entire rDNA coding region, rather than merely initiating transcription. Supporting this notion, our ChIP-seq and CUT&Tag data showed that Tcf15 occupancy correlates with H3K4me3 enrichment and reduced H3K27me3 deposition across the coding regions. Whether Tcf15 directly associates with the elongating RNA polymerase I complex or functions to preserve the open chromatin state during active transcription represents an open question for future investigation.

While the absence of DNA methylation on rRNA genes in ESCs was previously attributed to blocked methylation processes [10], our findings reveal that active DNA demethylation mediated by Tcf15‑Tet2 is equally essential, thereby adding a new regulatory layer. We propose that the balance between DNA methylation and demethylation on rRNA genes governs the chromatin state at rDNA loci. In ESCs, the highly expressed DEAD‑box helicase DDX18 prevents the deposition of repressive H3K27me3 marks on rRNA genes by binding and sequestering PRC2 in the outer nucleolar layer [11]. Here, we demonstrate that Tcf15 promotes H3K4me3 deposition to counteract PRC2‑mediated H3K27me3 modification; loss of Tcf15 leads to aberrant PRC2 recruitment to rDNA. Thus, an active nucleolar chromatin state in stem cells must be safeguarded by multiple regulatory pathways, and future efforts should aim to identify additional mechanisms. Notably, Tcf15 is also expressed in the most primitive subset of multipotent hematopoietic stem cells (HSCs), where it is both necessary and sufficient to drive long‑term self‑renewal [14]. Whether Tcf15 employs similar mechanisms to promote HSC self‑renewal merits future investigation.

Disruption of RiBi in ESCs can lead to diverse outcomes depending on the specific regulator affected. For example, loss of DDX10 [9], METTL3/METTL14 [40], or FBL [6] results in pluripotency exit, reduced proliferation, apoptosis, or impaired differentiation. In this study, we identify genomic instability as a novel phenotype associated with impaired RiBi in ESCs, occurring without disruption of pluripotency (S6 Fig). Our integrated RNA‑seq and Ribo‑seq analyses show that Tcf15 KD-induced RiBi collapse does not cause a uniform decline in protein synthesis; rather, it rewires translational profiles. Specifically, many transcripts encoding DNA replication and repair factors—including Parp1, which plays multifaceted roles in DNA damage response and repair [41], and Khdc3 (also known as Filia, an ESC‑specific genome guardian) [34]—are disproportionately downregulated at the translational level, even though their mRNA levels remain unchanged upon Tcf15 depletion. This translational deficit coincides with decreased MCM protein abundance and reduced replication origin density, providing a direct mechanistic link between RiBi dysfunction and the DNA replication stress and genomic instability observed in Tcf15‑depleted ESCs. The concomitant upregulation of stress‑responsive factors further suggests that this represents an active, adaptive translational response to nucleolar stress rather than a passive consequence of ribosome scarcity. Notably, although the protein levels of Oct4 and Klf4 are partially reduced in Tcf15‑KD cells owing to impaired translation, this does not lead to collapse of the pluripotency regulatory network (S6D–S6G Fig).

Our study has several limitations. First, while Tcf15 coordinates two independent effector complexes—Tcf15‑Rbbp5 and Tcf15‑Tet2—to ensure a hyperactive chromatin state, the functional significance of the Tet2‑mediated hyperactive state remains unclear, as do the determinants of their distinct functional contributions. Second, owing to the repetitive nature of the 200–300 rDNA copies, it is currently not possible to resolve whether these two protein complexes occupy the same or different rDNA repeats. Third, the mechanistic basis for why compromised RiBi selectively influences the translation of specific gene subsets remains an open question in the field.

## Materials and methods

### Cell preparation and culture

Mouse embryonic fibroblasts (MEFs) were isolated as described [42,43] with minor modifications. Briefly, pregnant CD1 mice at embryonic day 13.5 (E13.5) were euthanized, and embryos were dissected. After removing embryonic heads and visceral organs, the remaining tissues were minced into a homogeneous paste and digested with 0.25% trypsin-EDTA (Gibco, Cat. No. 25200−072) at 37°C for 10 min. Digestion was terminated using DMEM medium (Gibco, Cat. No. 11965) supplemented with 10% fetal bovine serum (FBS, Gibco, Cat. No. 10099141C). The cell suspension was transferred to a 50 mL centrifuge tube and allowed to stand at room temperature for 10 min to sediment tissue debris. MEFs in the supernatant were seeded into 100 mm culture dishes and cultured in DMEM medium containing 10% FBS in a humidified incubator at 37°C with 5% CO_2_. All animal care and experimental procedures were approved by the Institutional Animal Care and Use Committee of the Kunming Institute of Zoology, Chinese Academy of Sciences (Approval No. IACUC-RE-2026-07-005).

To prepare feeder cells, MEFs were expanded for three passages, followed by treatment with 5 μg/mL mitomycin C (Sigma, Cat. No. M4287) at 37°C for 4 hours. Inactivated MEFs were aliquoted, cryopreserved in liquid nitrogen, and used as feeders for culture of mouse ESCs upon recovery.

ESCs were routinely cultured on inactivated MEF feeders. The culture medium consisted of DMEM/F12 supplemented with 20% Knockout Serum Replacement (Gibco, Cat. No. 10828028), 2 mM L-glutamine (Sigma-Aldrich, Cat. No. G8540), 1 mM sodium pyruvate (Gibco, Cat. No. 11360070), 0.1 mM β-mercaptoethanol (Sigma-Aldrich, Cat. No. M7522), 1% non-essential amino acids (Gibco, Cat. No. 11140−035), and 1,000 U/mL mouse leukemia inhibitory factor (LIF, Millipore, Cat. No. ESG1107). Cultures were maintained in a humidified incubator at 37°C with 5% CO_2_. For transient knockdown experiments with the doxycycline-inducible system, ESCs were cultured in the presence of 1 μg/mL doxycycline for 48 hours. Cells were cultured at approximately passage 20 and kept in logarithmic growth throughout treatment. Cells were plated at optimized seeding density to achieve 60%–70% confluence at the 48 h endpoint, with no intermediate passaging performed. The short 48-hour induction minimizes long-term cellular adaptive responses. qRT-PCR was used to verify gene knockdown efficiency.

### Lentiviral packaging, transduction and generation of stable cell lines

For gene knockdown, shRNA sequences targeting the gene of interest (S7 Table) were cloned into either the doxycycline-inducible expression pTRIPZ vector or the constitutive expression pLKO.1 vector. For gene overexpression, the coding sequence (CDS) of Tcf15 with a C-terminal Flag tag was subcloned into the pTOMO-IRES-EGFP vector (Addgene #26,291).

Lentiviral particles were produced by co-transfecting these constructs with the packaging plasmids psPAX2 and pMD2.G into 293T cells at 70%–80% confluency, using a DNA ratio of 2:1:1 (target vector: psPAX2: pMD2.G). Specifically, 6 μg of target vector, 3 μg of psPAX2, and 3 μg of pMD2.G were used per 10-cm dish. Viral supernatants were harvested at 48 and 72 hours post-transfection, filtered through a 0.45 μm membrane, and concentrated by ultracentrifugation at 25,000 × g at 4 °C for 2.5 hours. The concentrated lentiviral particles were aliquoted and stored at −80°C until use.

To generate stable knockdown cell lines, after 48 hours post-lentiviral infection, cells were selected with 0.5 μg/mL puromycin for 72 hours. For Tcf15 overexpression, virus-transduced cells were trypsinized and plated at low density (100–200 cells per 10-cm dish). After 3–5 days, GFP-positive colonies were manually picked and expanded.

### Cell fractionation

Fractions of cytoplasm, nuclei, nucleoplasm, and nucleoli were isolated as described [44] with minor modifications. All buffers, including nucleolar standard buffer (NSB) supplemented with 1 × protease inhibitor, and sucrose/MgCl_2_ solutions were prepared using DEPC-treated water. Cells (2 × 10⁷) from three 100-mm dishes were resuspended in NSB containing vanadyl ribonucleoside complex to a final volume of 1.5 mL, incubated on ice for 30 min, lysed with 0.3% NP-40, and homogenized 25 times using a Dounce homogenizer. The cytoplasmic fraction was collected by centrifugation at 1,200 × g for 5 min at 4°C. Nuclear pellets were resuspended in 250 mM sucrose/10 mM MgCl_2_, overlaid onto 880 mM sucrose/5 mM MgCl_2_, and centrifuged at 1,200 × g for 10 min at 4°C to purify nuclei. For nucleoli isolation, isolated nuclei were sonicated on ice (30-s bursts with 30-s intervals, total 15 min), then overlaid onto 880 mM sucrose/5 mM MgCl_2_ and centrifuged at 2,000 × g for 20 min at 4°C. Nucleoli were resuspended in 0.34 M sucrose buffer.

### Real-time quantitative reverse transcription PCR (qRT-PCR)

Total RNA was isolated from cells following the protocol provided with TRNzol reagent (Tiangen). For each sample, 1 μg of total RNA was subjected to reverse transcription using the PrimeScript RT Reagent Kit with gDNA Eraser (Takara, Cat. No. RR037A), strictly according to the manufacturer’s instructions. This kit simultaneously removes genomic DNA contamination during the reverse transcription process.

Quantitative real-time PCR (qRT-PCR) was performed on the CFX96 Real-Time System (Bio-Rad) using TB Green Premix Ex Taq II (Takara, Cat. No. RR820A). *Gapdh* served as the internal reference gene to normalize the data. The sequences of the primers utilized in this experiment are detailed in S8 Table.

### CUT&Tag, ChIP-seq and data process

CUT&Tag libraries were constructed using the Hyperactive Universal CUT&Tag Assay Kit for Illumina Pro (Vazyme, TD903) in accordance with the manufacturer’s protocol.

ChIP was performed as described [45] with modifications. 1 × 10⁷ cells were cross-linked with 1% formaldehyde (10 min), quenched with 0.14 M glycine (30 min), washed with cold PBS, and lysed in 0.5% SDS buffer with protease inhibitors. Lysates were sonicated and incubated with 5–10 μg of antibody overnight at 4°C. Protein A/G beads were added for 3 h, washed with low-salt, high-salt, LiCl, and TE buffers, and eluted at 65°C. DNA was recovered and purified using the MinElute PCR Purification Kit for library prep and qPCR.

Libraries were sequenced on the Illumina NovaSeq 6000 platform. Clean Reads were mapped to the mouse genome mm10 suitable for rDNA mapping [46] using the ‘Bowtie2′ program. PCR duplicates were removed using SAMtools (v.1.19.2) [47] and the bigWig files were transformed using deepTools (v.3.5.4) [48]. Peaks were called using the MACS2 software (v.2.2.5) [49] with a q-value cutoff of 0.01.

### DNA methylation sequencing and data analysis

After expansion, approximately 1 × 10^7^ cells were harvested, digested with trypsin, washed once with PBS, and the cell pellet was snap-frozen in liquid nitrogen. Samples were then sent to Mingma (Shanghai) Biotechnology Co., for standardized DNA extraction, bisulfite conversion, library preparation, and whole-genome bisulfite sequencing (WGBS) on the NovaSeq 6,000 platform. Raw sequencing reads were processed using TrimGalore (v0.6.10) to remove adapter sequences and low-quality reads. The clean reads were aligned to the mouse reference genome (mm10) optimized for ribosomal DNA mapping [46] using Bismark (v0.24.2) [50]. Differentially methylated regions (DMRs) were analyzed using the methylKit R package (v1.6.0) [51], with the screening threshold set at q-value < 0.05.

### Quantification of rDNA CpG methylation by qPCR

rDNA CpG methylation levels were assessed using a restriction enzyme-based quantitative PCR approach, adapted from previously described methods [10,52]. Briefly, 2 μg of genomic DNA was subjected to digestion with the restriction endonuclease HpaII (New England Biolabs). Quantitative PCR was performed using two distinct primer pairs: (1) a methylation-sensitive pair (−165/-145 forward and −20/-1 reverse) that flanks the CCGG restriction motif at position −142 in the rDNA promoter, and (2) a reference pair (+1/ + 20 forward and +111/ + 130 reverse) targeting a neighboring sequence devoid of HpaII recognition sites. Relative methylation status was determined by analyzing ΔCt differences between experimental and control cohorts, where the abundance of target amplicons (from −165 to −1) normalized to reference amplicons (from +1 to +130) indicates the degree of resistance to HpaII digestion. This resistance phenotype directly correlates with the CpG methylation levels within the rDNA promoter region.

### Immunofluorescence staining

Cells were seeded on coverslips and cultured until reaching 60%–70% confluency. For optional nucleocytoplasmic separation, cells were treated with CSKT solution (CSKT: Cytoskeleton Extraction Buffer, a commonly used buffer for mild cytoskeleton dissociation and preliminary nucleocytoplasmic separation) consisting of 10 mM PIPES (pH 6.8), 100 mM NaCl, 300 mM sucrose, 3 mM MgCl_2_, 0.5% (v/v) Triton X-100, and 1 mM phenylmethylsulfonyl fluoride (PMSF) at 4°C for 10 min, followed by three washes with PBS (10 min each). Subsequently, cells were fixed with 4% paraformaldehyde (PFA) at room temperature for 15 min and washed three times with PBS (10 min each). Permeabilization was performed using PBS containing 0.2% Triton X-100 for 10 min, followed by three PBS washes (10 min each). Cells were then blocked with 5% bovine serum albumin (BSA) in PBS at room temperature for 1 h. After blocking, cells were incubated with diluted primary antibodies at 4°C overnight, washed three times with PBS (10 min each), and then incubated with appropriate secondary antibodies for 2 h at room temperature in the dark. Following another three PBS washes (10 min each), nuclei were counterstained with DAPI. Images were acquired using an Olympus FV1000 confocal microscope.

For 5-methylcytosine (5mC) immunofluorescence staining, cells were fixed with pre-chilled 70% ethanol for 5 min at room temperature, followed by three 10-minute PBS washes. DNA denaturation was performed using 1.5 N HCl for 30 min at room temperature, and residual HCl was removed via three 10-minute PBS washes. Cells were then permeabilized with 0.3% Triton X-100 in PBS for 30 min at room temperature, neutralized with 0.1 M sodium borate (Na_2_B_4_O_7_) for 30 min at room temperature. Following blocking with 5% BSA for 1 hour at room temperature, cells were incubated overnight at 4°C with rabbit anti-5mC primary antibody diluted 1:500 in PBS containing 1% BSA and 0.3% Triton X-100 (single staining condition). After three 10-minute washes with PBS, cells were incubated with species-appropriate secondary antibodies for 1 hour at room temperature under light-protected conditions. Following another series of three 10-minute PBS washes, nuclear counterstaining was performed using DAPI for 10 min at room temperature. Finally, after a final set of three 10-minute PBS washes, coverslips were mounted for microscopic observation.

### Immunoblotting

Total protein was extracted from cells using RIPA lysis buffer (Beyotime, P0013J). Protein extracts were resolved by SDS-PAGE (6%–12% gels) and transferred onto polyvinylidene fluoride (PVDF) membranes (Roche, 03010040001). Membranes were blocked with 5% non-fat milk or 5% BSA for 1 h at room temperature, followed by an overnight incubation with primary antibodies at 4°C. After three washes with TBST (10 min each), membranes were incubated with appropriate HRP-conjugated secondary antibodies for 1 h at room temperature. Antigen-antibody complexes were detected by enhanced chemiluminescence (ECL) using Beyotime (P0018FS) reagents, and images were acquired with a ProteinSimple FluorChem system (FM0561). Antibody information was listed in S9 Table.

### Immunoprecipitation

A total of 1 × 10^7^ cells were lysed using RIPA lysis buffer (Beyotime, Cat. No. P0013J) supplemented with 1 × protease inhibitor (Beyotime, Cat. No. P1006). For each immunoprecipitation, 40 μL of Protein G magnetic beads (Thermo, Cat. No. 88847) were first conjugated with 2.5 μg of the target antibody (or 2.5 μg of isotype control IgG, Sigma, Cat. No. I5006) in PBS at 4°C for 2 hours. The antibody-bound beads were then washed and added to the cell lysate, followed by incubation at 4°C overnight. After incubation, the beads were collected and washed three times with IP washing buffer. Bound proteins were eluted by adding 30 μL of 2 × SDS loading buffer and boiling at 100°C for 10 min. The eluted proteins were then subjected to mass spectrometry analysis or Western blotting.

### Dot-blot assay

Genomic DNA (gDNA) was extracted by incubating cell samples with 5 mL of lysis buffer (50 mmol/L Tris, 50 mmol/L EDTA, 1% SDS, pH 8.0) and 30 μL of 20 mg/mL Proteinase K (Beyotime, Cat. No. ST535−100 mg) at 55 °C overnight. After adding 30 μL of 10 mg/mL RNase A, the mixture was inverted 25 times and incubated at 37 °C for 30 min to eliminate RNA contamination. Subsequently, gDNA was precipitated with 5 mL of 100% isopropanol, centrifuged at ≥4,000 × g for 10 min, the supernatant was discarded, and the pellet was washed with 5 mL of freshly prepared 70% ethanol. The pellet was then transferred to a clean centrifuge tube, resuspended in 300–500 μL of ultrapure water, incubated at 65 °C for 1 h, and left at room temperature overnight for complete dissolution.

For dot-blot analysis, a nitrocellulose (NC) membrane was marked with grids using a pencil, an appropriate volume of gDNA was spotted at the center of each grid (diameter controlled at 2–4 mm) and air-dried at room temperature to immobilize DNA. The membrane was blocked with TBS-T buffer containing 5% non-fat milk at room temperature for 30–60 min, followed by sequential incubation with primary and secondary antibodies for immunoblotting. After incubation with enhanced chemiluminescence (ECL) reagent, images were captured by Protein Simple, and quantitative analysis was performed using ImageJ software.

### Identification of Tcf15 interaction proteins

To identify Tcf15 interaction proteins, we fused 3×HA-tagged Tcf15 to TurboID biotin ligase using Gibson Assembly. Then, the Tcf15–3HA-TurboID fragment was cloned into the PiggyBac expression vector pPB-CAG-IRES-Puro (kindly provided by Professor Jie Na at Tsinghua University). ESCs were transfected with hyperPB mRNA and the pPB-CAG-IRES-Puro vector carrying the Tcf15–3HA-TurboID fragment using Lipofectamine 2000 for 48 h and selected with 0.5 μg/mL puromycin for 72 h. Expression of Tcf15–3HA-TurboID was verified by western blot using anti-HA (1:5000, Proteintech) antibody. For proximity labeling, cells were incubated with 100 μM biotin for 30 min at 37°C. Reactions were quenched with 3–5 washes of ice-cold PBS containing 1 mM PMSF. Cells were lysed in RIPA buffer (50 mM Tris-HCl pH 7.4, 150 mM NaCl, 1% NP-40, 0.5% sodium deoxycholate, 0.1% SDS) supplemented with protease inhibitors. Biotinylated proteins were enriched using streptavidin magnetic beads (Thermo Fisher Scientific) according to the manufacturer’s protocol. The enriched proteins were subjected to mass spectrometry to identify Tcf15-proximal proteins.

### Karyotyping

Logarithmically growing cells were incubated for 2 hours in fresh medium supplemented with colchicine (final concentration 0.05 μg/mL). Following routine trypsinization, cells were harvested and centrifuged at 1,000 rpm for 5 min. The cell pellet was loosened by gently flicking the tube, and then resuspended in 1 mL of pre-prepared hypotonic solution (10 mM Tris-HCl [pH 7.4], 40 mM glycerol, 20 mM NaCl, 1.0 mM CaCl_2_, 0.5 mM MgCl_2_) and incubated at room temperature for 15 min. After hypotonic treatment, cells were centrifuged again; the supernatant was discarded, and 3 mL of freshly prepared methanol-glacial acetic acid fixative (3:1, v/v) was added dropwise with gentle pipetting to mix. Fixation was repeated once, and the pellet was finally resuspended in an appropriate volume of fixative adjusted to cell density. Aliquots of the cell suspension were dropped onto pre-cleaned glass slides and air-dried at room temperature. Chromosome spreads were stained with Giemsa solution (Invitrogen; cat. #10,092−013) for 10 min at room temperature, rinsed with water, and subjected to chromosome counting. At least 40 metaphases were examined per replicate, and three independent replicates were conducted.

### Micronuclei assay

ESCs were cultured under standard conditions until reaching 70%–80% confluence. Cells were fixed with 4% (w/v) paraformaldehyde on ice for 20 min and stained with DAPI to visualize nuclear morphology. For each sample, micronucleus formation was evaluated by counting at least 1,000 cells from multiple random fields using an Olympus FV1000 confocal microscope. The experiment was independently repeated three times. The micronuclei frequency was expressed as the number of micronuclei per total cells examined.

### Neutral comet assay

The neutral comet assay was performed according to a previously described protocol [34] with minor modifications. Briefly, glass slides were precoated by immersion in 0.8% normal melting point agarose for 5 seconds and air-dried. Following treatment, ESCs were digested into a single-cell suspension and adjusted to a density of 1 × 10⁶ cells/mL. Ten μL of cell suspension was mixed with 70 μL of 0.8% low melting point agarose maintained at 37°C, and the mixture was immediately spread evenly onto the precoated slide. After covering with a coverslip, the slide was solidified at 4°C for 10 min. The coverslip was then gently removed, and the slide was immersed in neutral lysis buffer (10 mM Tris, 2.5 M NaCl, 1% sodium sarcosinate, 100 mM Na_2_EDTA, 1% Triton X-100, pH 8.3) for 1 h at room temperature. After rinsing with distilled water, the slide was subjected to unwinding in neutral electrophoresis buffer (300 mM sodium acetate, 100 mM Tris, pH 8.3) for 20 min, followed by electrophoresis at 35 V and 80 mA for 30 min. Subsequently, the slide was dehydrated in anhydrous ethanol and air-dried. DNA was stained with 1 μg/mL DAPI (Invitrogen, D1306) for 30 min, followed by three washes with distilled water. Images were randomly acquired using a fluorescence microscope, and at least 200 cells per sample were analyzed using CASP software. The experiment was independently repeated three times.

### OPP nascent protein synthesis assay

To visualize protein synthesis within single mouse embryonic stem cells, the Click-iT Plus OPP Alexa Fluor 488 Protein Synthesis Assay Kit (Molecular Probes, C10456) was employed according to the manufacturer’s protocol. Briefly, O-propargyl-puromycin (OPP) was added to cultured cells to allow its incorporation into nascent polypeptides during active translation. Subsequently, a chemoselective ligation—or “click” reaction—was performed using Alexa Fluor 488 picolyl azide and Click-iT reaction cocktail to conjugate the fluorescent dye to OPP-labeled proteins, enabling sensitive detection via fluorescence imaging or flow cytometry analysis.

### RNA-seq and data analyses

Total RNA was extracted using TRNzol reagent (Tiangen) according to the manufacturer’s instructions. RNA-seq libraries were prepared using the NEBNext Ultra II RNA Library Prep Kit for Illumina. Libraries were sequenced on an Illumina NovaSeq 6,000 platform. For data analysis, clean reads were aligned to mm10 using HISAT2 (v2.2.1) [53]. FPKM (Fragments Per Kilobase of transcript per Million mapped reads) values were calculated using Cufflinks software. Differentially expressed genes (DEGs) were identified using Cuffdiff2 (q < 0.05 and fold change > 2). Heatmaps were generated using the gplots package (v3.2.0).

### Polysome profiling

Polysome profiling assay was performed as described [54]. Cells were treated with cycloheximide (CHX) at a final concentration of 0.1 mg/mL and incubated at 37°C for 1 min. Subsequently, the cells were washed three times with ice-cold PBS containing 100 µg/mL cycloheximide. A total of 2 × 10^7^ cells were collected and lysed in 500 µL of lysis buffer (25 mM Tris-HCl pH 7.4, 5 mM MgCl_2_, 100 mM NaCl, 1% NP-40, 1% sodium deoxycholate, 40 U/mL RNase inhibitor, 1 × protease inhibitor cocktail, 100 µg/mL CHX, and 1 mM DTT) on ice for 30 min. The lysate was centrifuged at 1,300 × g for 10 min at 4°C. The supernatant was loaded onto a 10%–50% sucrose gradient and subjected to ultracentrifugation for 3 hours at 35,000 rpm using an SW41 rotor. Fractions (1 mL each) were continuously collected by puncturing the bottom of the ultracentrifuge tube with a needle. Absorbance at 254 nm was monitored using a UV detector to generate the polysome profile.

### Ribosome profiling sequencing and data analysis

Mouse ESCs were cultured in 15 cm diameter dishes. Cells were washed with fresh medium containing 0.1 mg/mL cycloheximide (CHX), incubated at room temperature for 1 min, and immediately placed on ice. They were then gently rinsed once with 10 mL pre-chilled PBS supplemented with 0.1 mg/mL CHX, and the liquid was aspirated. Subsequently, 1–2 mL pre-chilled PBS containing 0.1 mg/mL CHX was added, and cells were collected via unidirectional scraping using a disposable cell scraper. The cell suspension was transferred to pre-chilled 1.5 mL centrifuge tubes (remaining cells were collected after centrifugation and supernatant removal if transfer was incomplete). Cell pellets were obtained by centrifugation at 500 × g for 5 min at 4°C in a pre-cooled centrifuge, with the supernatant discarded. Finally, cell pellets were snap-frozen in liquid nitrogen and shipped on dry ice to Novogene Co., for subsequent library preparation and sequencing. To remove adapters and low-quality reads from Ribo-seq data, raw reads were trimmed using TrimGalore (v0.6.10) with the following parameters: -j 3 -q 25 --phred33 --length 25 -e 0.1 --stringency 4. Reads aligning to ribosomal RNAs (rRNAs: 18S, 28S, 5S, and 5.8S) were subsequently filtered out using Bowtie2 (v2.5.2). Clean reads were mapped to the mouse reference genome (mm10) using HISAT2 (v2.2.1) [53]. Sequencing quality was assessed using the R package RiboseQC (v0.99.0) [55]. Raw counts of genes were generated with HTSeq-count (v2.0.5) [56]. Finally, translation efficiency was calculated and compared between the KDC and KD groups (using corresponding RNA-seq input data) with the R package Xtail (v1.2.0) [57]. Genes with a fold change > 2 and false discovery rate (FDR) < 0.05 were defined as differentially translated.

### DNA fiber assay

DNA fiber assay was performed as previously described [35]. For the fork restart assay, cells were pulse-labeled with 50 μM 5-iodo-2′-deoxyuridine (IdU, Sigma, I7125) for 30 min, washed three times with warm PBS, and then treated with or without 4 mM hydroxyurea (HU, Sigma, H8627) for 4 h. After washing, cells were labeled with 50 μM 5-chloro-2′-deoxyuridine (CIdU, Sigma, C6891) for 30 min. For the newly initiated fork assay, 0.1 mM HU was concurrently added during IdU labeling. Cells were then harvested, washed, and resuspended at a concentration of 10³/μL. 2.5 μL of cell suspension was deposited onto one end of a glass slide, followed by overlay with 7.5 μL of lysis buffer (50 mM EDTA, 0.5% SDS, 200 mM Tris–HCl, pH 7.5) and incubation for 8 min at room temperature. Slides were tilted to allow gradual stream flow for DNA fiber spreading, air-dried, fixed in methanol/acetic acid (3:1, v/v) for 10 min, and then denatured in 2.5 M HCl at 4 °C overnight. Immunofluorescence staining was carried out using anti-IdU and anti-CIdU antibodies. DNA fibers were visualized and analyzed using an Olympus FV1000 confocal microscope. Statistical analysis was performed based on measurements from at least 200 DNA fibers per sample, and each experiment was independently repeated three times.

### Gene Ontology analysis

Gene Ontology (GO) terms were enriched using the DAVID web tool [58].

### Statistical analysis

The statistical tests with the corresponding sample size were listed in the text and the legends of relevant figures. Statistical analyses were conducted using GraphPad Prism 8.0 (GraphPad Software, La Jolla, CA, United States) by two-tailed Student *t* test. *P* < 0.05 was defined as statistically significant. All experimental data were presented as the mean ± SEM.

## Supporting information

S1 Fig Tcf15 is localized in the nucleolus of ESCs. (**A**) Western blot confirmed the overexpression of Flag-Tcf15 in wild-type (WT) ESCs with a C57BL/6J genetic background. (**B**) qRT-PCR validated that *Tcf15* was efficiently knocked down via two independent short hairpin RNAs in ESCs. (**C**) Western blot validated the expression of Flag-Tcf15 in KD-rescue ESCs. (**D**) Bright-field images of KDC, Tcf15 KD and KD-rescue ESCs. Scale bar, 100 μm. (**E**) qRT-PCR showed that WT and KD-rescue ESCs had comparable levels of *Tcf15* mRNAs. (**F-G**) Immunofluorescence staining showed Flag-Tcf15 in KD-rescue ESCs was consistently localized in the nucleolus marked by NPM1, no matter whether cultured in KSR/LIF medium (**F**) or 2i/LIF condition (**G**). (**H**) Flag-Tcf15 was also detected in the nucleolus in R1 mouse ESCs. Scale bar, 10 μm in (**F**, **G**, **H**). (**I**) Genomic distribution of non‑rDNA Tcf15 binding peaks identified by ChIP‑seq in Flag‑Tcf15 overexpressing ESCs. The mRNA levels of Tcf15 in (**B**) and (**E**) were normalized by *Gapdh*. Experiments were repeated three times using independent biological samples, and similar results were obtained. Data were presented as mean ± SEM. Two-tailed Student *t* test, ****P < 0.0001; ns, not significant. The data underlying this figure can be found in S10 Table.
(TIF)

S2 Fig Tcf15 suppresses repressive epigenetic modifications in ESCs. (**A-C**) Immunofluorescence staining showed that Tcf15 KD reduced the levels of H3K4me3 (**A**), but increased the levels of DNA 5mC methylation (**B**). These epigenetic defects could be reversed in KD-rescue ESCs. However, H3K9me3 modification was not affected by Tcf15 KD (**C**). (**D**) Immunoblotting of whole cell lysates confirmed the epigenetic changes of H3K4me3 and H3K27me3 in Tcf15 KD ESCs. Gapdh served as a loading control, and the relative levels of H3K4me3 and H3K27me3 were shown in the right panel. (**E**) Dot blot analysis of the nuclear DNA showed that Tcf15 KD increased DNA m5C methylation levels. dsDNA was used as a loading control. The fold changes of methylation level were quantified in the right panel. Experiments were repeated three times using independent biological samples, and similar results were obtained. Data were presented as mean ± SEM. Two-tailed Student *t* test, **P < 0.01, ***P < 0.001, ****P < 0.0001; ns, not significant. The data underlying this figure can be found in S10 Table.
(TIF)

S3 Fig Transient knockdown of Tcf15 increased H3K27me3 modification and DNA methylation in nucleoli. (**A**) qRT-PCR validated the decreased mRNA expression of *Tcf15* after doxycycline (Dox)-induced transient KD for 48 h. *Gapdh* served as an internal control. (**B**) Immunofluorescence staining of H3K27me3 and NPM1 in transient Tcf15 KD ESCs. Note that H3K27me3 level was increased in the nucleolus. Scale bar, 10 μm. (**C**) qPCR validated the increased level of CpG methylation in the nucleolus after transient KD of Tcf15 for 48 h. Experiments were repeated three times using independent biological samples, and similar results were obtained. Data were presented as mean ± SEM. Two-tailed Student *t* test, **P < 0.01. The data underlying this figure can be found in S10 Table.
(TIF)

S4 Fig Tcf15 interacts with either Tet2 or Rbbp5, which mediate the functions of Tcf15. (**A**) Streptavidin blotting of cell lysates in the presence or absence of biotin. Ligase expression was detected by anti-HA blotting. (**B**) Silver staining of streptavidin-enriched eluates obtained from cells with or without biotin treatment. (**C**) Using whole cell lysates, reciprocal immunoprecipitation followed by immunoblotting validated the interaction of Tcf15 with Tet2 or Rbbp5. No physical association was detected between Tet2 and Rbbp5. (**D-E**) Western blot validated the efficient knock down of Tet2 (**D**) or Rbbp5 (**E**) in ESCs. Gapdh was used as a loading control. (**F-G**) Immunofluorescence staining showed that KD of Tet2 or Rbbp5 increased the H3K27me3 level (**F**), whilst decreased the H3K4me3 modification (**G**). (**H-I**) Western blot analysis of whole cell lysates indicated that KD of Tet2 (**H**) or Rbbp5 (**I**) increased H3K27me3 level, but decreased H3K4me3 modification in ESCs. Gapdh was used as a loading control. Experiments were repeated three times using independent biological samples, and similar results were obtained. Data were presented as mean ± SEM. Two-tailed Student *t* test, *P < 0.05, **P < 0.01, ***P < 0.001, ****P < 0.0001. The data underlying this figure can be found in S10 Table.
(TIF)

S5 Fig Loss of Tcf15 impairs the translation of a specific subset of genes. (**A**) Quality control of Ribo-seq. Frame analysis was used to verify that the sequences obtained by Ribo-seq correspond to authentically translated open reading frames (ORFs). Frame 0 (red) corresponds to the first reading frame immediately following the start codon. Frame 1 (blue) and Frame 2 (green) correspond to the reading frames shifted by 1 base and 2 bases, respectively, relative to the start codon. (**B**) Heatmap showing the differentially expressed genes in Tcf15 KD versus KDC ESCs. (**C**) Volcano plot analysis of quantitative mass spectrometry data identified the proteins which were prevalently expressed in mouse embryonic fibroblasts (MEFs, blue, 1947 proteins) or in ESCs (red, 1745 proteins). (**D**) Gene Ontology (GO) enrichment analysis identified the top biological processes enriched for the ESC-prevalent proteins. The data underlying this figure can be found in S10 Table.
(TIF)

S6 Fig Tcf15-Rbbp5 axis maintains genome stability independent of pluripotency. (**A**) Immunoblotting and quantification of γH2AX protein levels in KDC, Rbbp5-KD1, Rbbp5-KD2 cells, with Tubulin as the loading control. (**B**) Representative immunofluorescence staining for γH2AX (green) and DAPI (blue) in Rbbp5-knockdown cells, with corresponding quantitative analysis of the mean fluorescence intensity. Scale bar, 10 μm. (**C**) Representative images and quantitative tail moment analysis from the neutral comet assay, showing increased DNA double-strand breaks in Rbbp5-KD ESCs. (**D**) Alkaline phosphatase (AP) staining of KDC, Tcf15-KD1 and Tcf15-KD2 ESCs. Scale bar, 0.2 cm. (**E**) qRT-PCR quantification of core pluripotency marker genes (Oct4, Nanog, Klf4, Sox2) in KDC, Tcf15-KD1 and Tcf15-KD2 cells. (**F**) Quantification of EB formation efficiency. (**G**) Representative bright-field images of embryoid bodies (EBs) derived from KDC, Tcf15-KD1 and Tcf15-KD2 cells at day 5. Scale bar, 50 μm. The size of EBs was quantified in the right panel. Distribution of day 5 EB diameters across groups; red lines indicate median values. Experiments were repeated three times using independent biological samples, and similar results were obtained. At least 40 EBs per group were analyzed. Data were presented as mean ± SEM. Two-tailed Student *t* test, *P < 0.05, **P < 0.01, ***P < 0.001, ****P < 0.0001. ns, not significant. The data underlying this figure can be found in S10 Table.
(TIF)

S1 Table Tcf15 binding sites annotation. Excel table. Columns list the genomic coordinate information and functional annotation of each Tcf15 binding site, including chromosome, peak start position, peak end position, regional genomic annotation, target gene ID, and distance from peak to gene transcription start site (TSS).
(XLSX)

S2 Table Tcf15 interaction proteins. Excel table. First column gives gene names. Second column gives edgeR estimated log ratios. Remaining columns indicate edgeR estimated *p*-values and adjust *p*-values.
(XLSX)

S3 Table Differentially expressed genes between Tcf15 knockdown and control upon Tcf15 knockdown. Excel table. First column gives gene names of Differentially expressed genes. Remaining columns give FPKM of Tcf15 knockdown control (KDC) repeat1, FPKM of KDC repeat2, FPKM of Tcf15 shRNA1 knockdown (KD1) repeat1, FPKM of KD1 repeat2, FPKM of Tcf15 shRNA2 knockdown (KD2) repeat1, and FPKM of KD2 repeat2.
(XLSX)

S4 Table Genes with differential translation efficiency in Tcf15 knockdown ESCs. Genes with upregulated translation efficiency (TE) in Tcf15-knockdown ESCs are listed in Sheet 1. Conversely, genes with downregulated TE in Tcf15-knockdown ESCs are listed in Sheet 2, and the relevant GO enrichment results are presented in Sheet 3.
(XLSX)

S5 Table GO enrichment of genes with elevated translation efficiency upon Tcf15 knockdown. Excel table. Columns list GO biological process terms, the number of enriched genes (Count), percentage of background genes (%), raw P-value, corresponding enriched gene symbols separated by commas, and adjusted FDR value.
(XLSX)

S6 Table ESC-prevalent proteins overlap with proteins exhibiting decreased translation efficiency in Tcf15 knockdown ESCs. Proteins upregulated in mouse embryonic stem cells (mESCs) relative to mouse embryonic fibroblasts (MEFs) are listed in Sheet 1, with their corresponding GO enrichment results provided in Sheet 2. Additionally, proteins highly expressed in ESCs and whose translation efficiency is reduced upon Tcf15 knockdown are listed in Sheet 3, and the GO enrichment analysis results for these proteins are detailed in Sheet 4.
(XLSX)

S7 Table Short hairpin RNA sequences used for gene knock down. (DOCX)

S8 Table Primers for PCR cloning and RT-PCR. (DOCX)

S9 Table Antibody information. (DOCX)

S10 Table Raw data for all figures including all values used in the experiments. (XLSX)

S1 Raw Images Original gels with no adjustments. (PDF)

S1 Code Custom code used for data processing and figure generation. (ZIP)
