# Supplementary material for: Tcf15 promotes an open nucleolar chromatin state to safeguard ribosome biogenesis and genome stability in mouse embryonic stem cells
Source: PLoS Biol. 2026 Aug 25;24(8):e3003954. doi: 10.1371/journal.pbio.3003954 (PMC13505954; doi:10.1371/journal.pbio.3003954)

Figure 1B

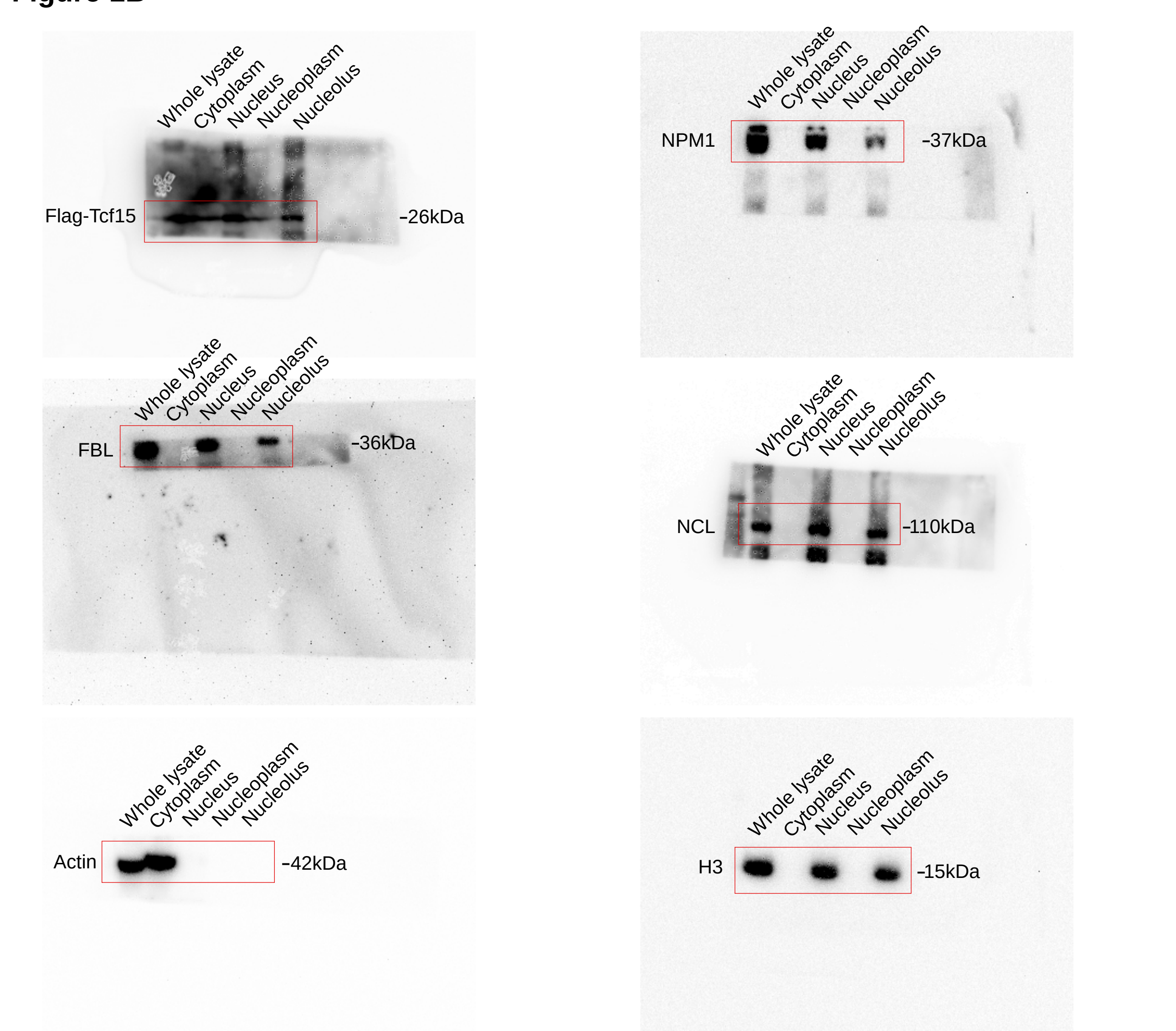

Figure 2B

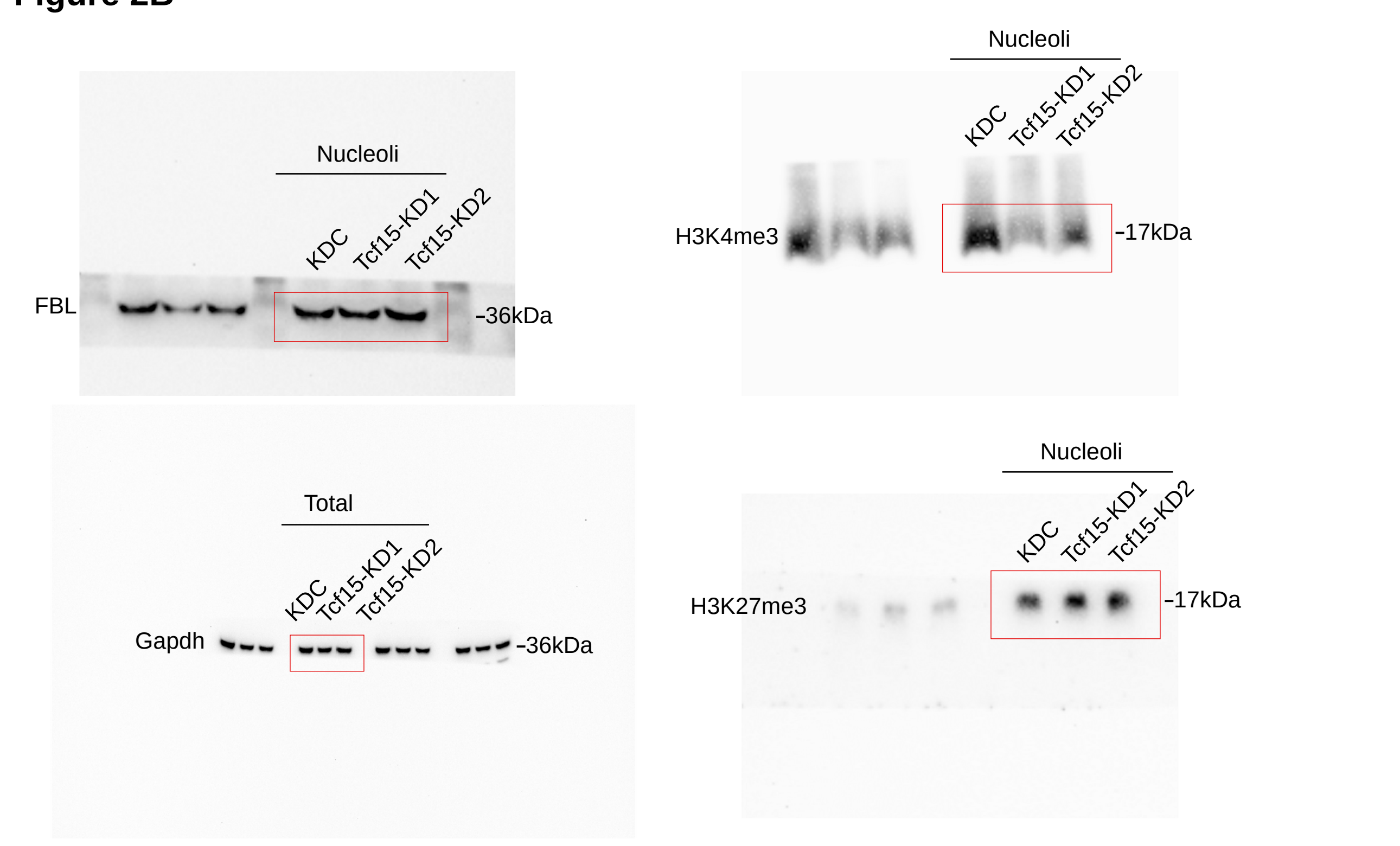

Figure 2C

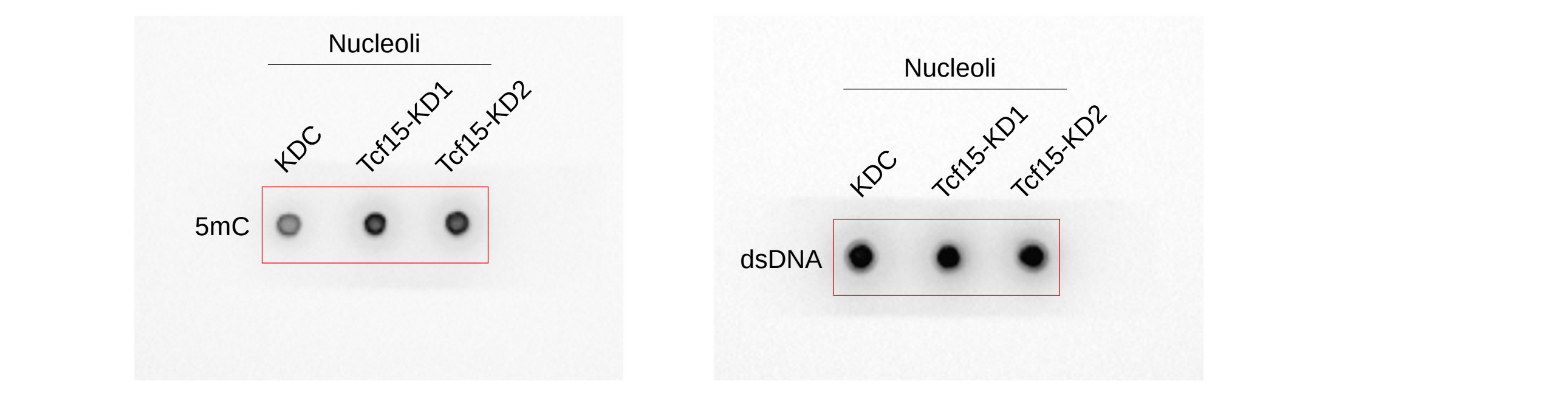

Figure 3B

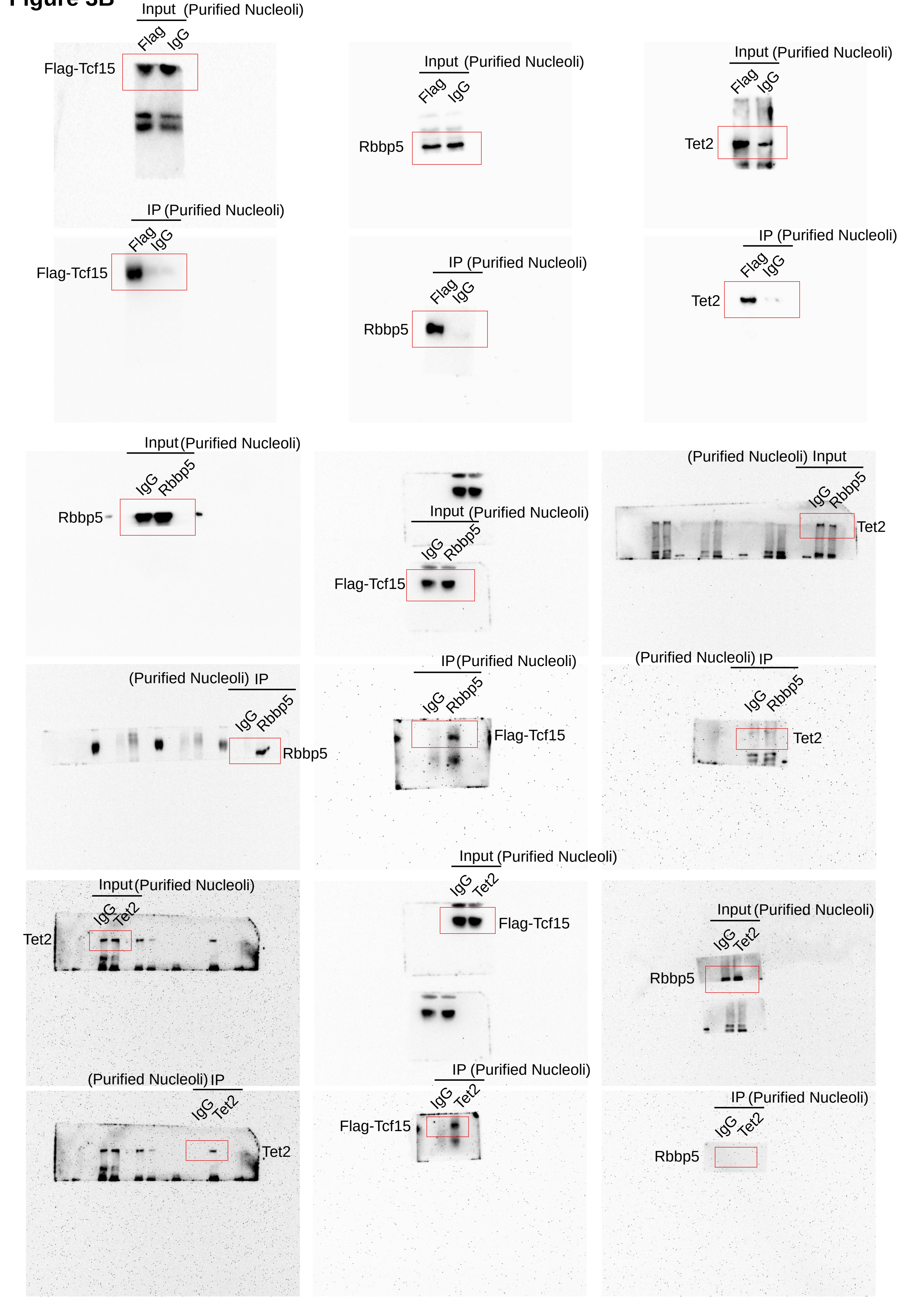

Figure 3G

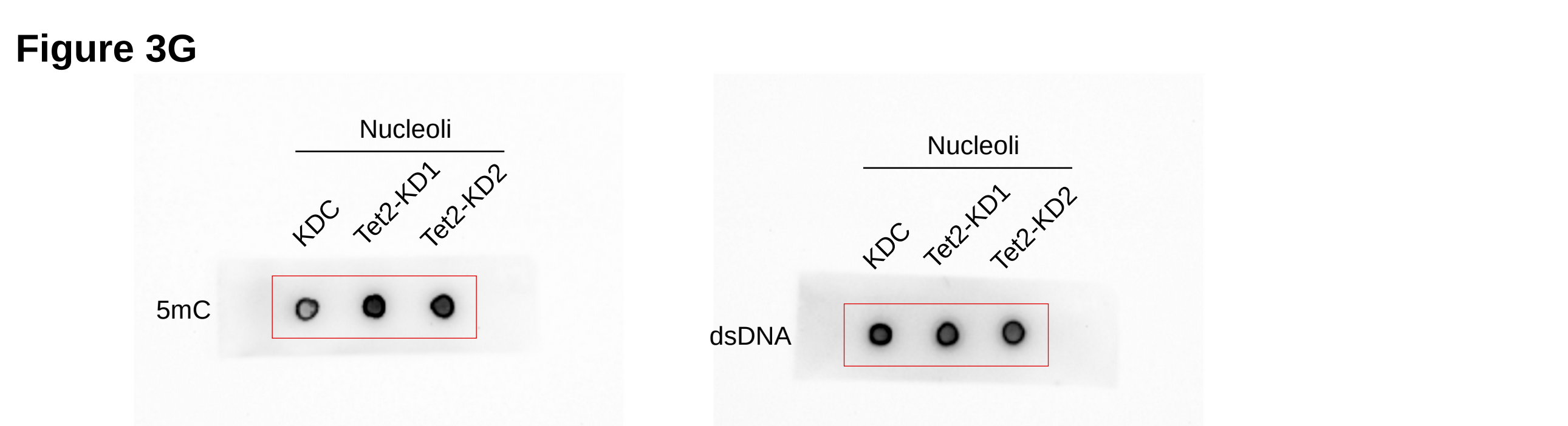

Figure 3H

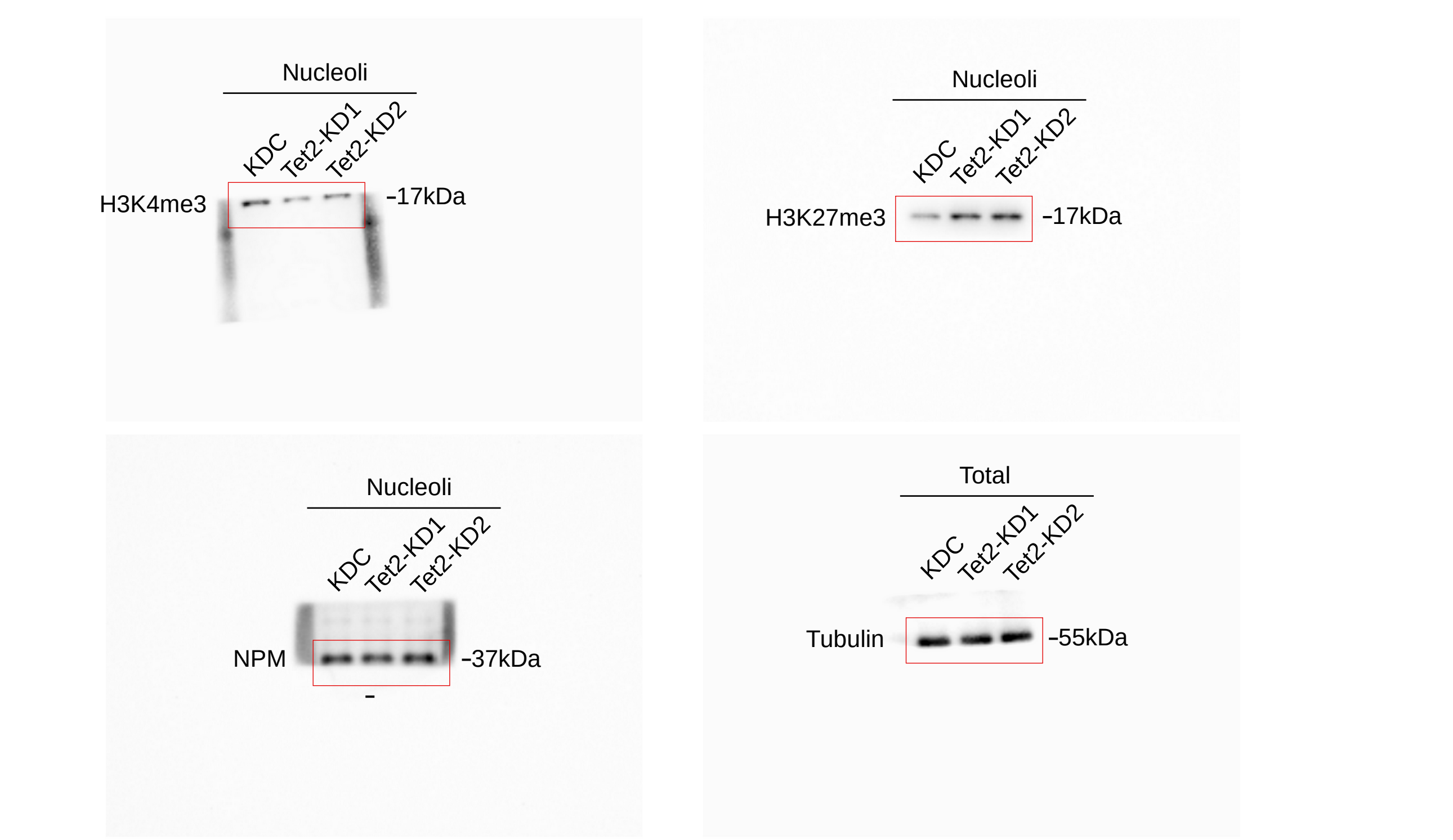

Figure 3I

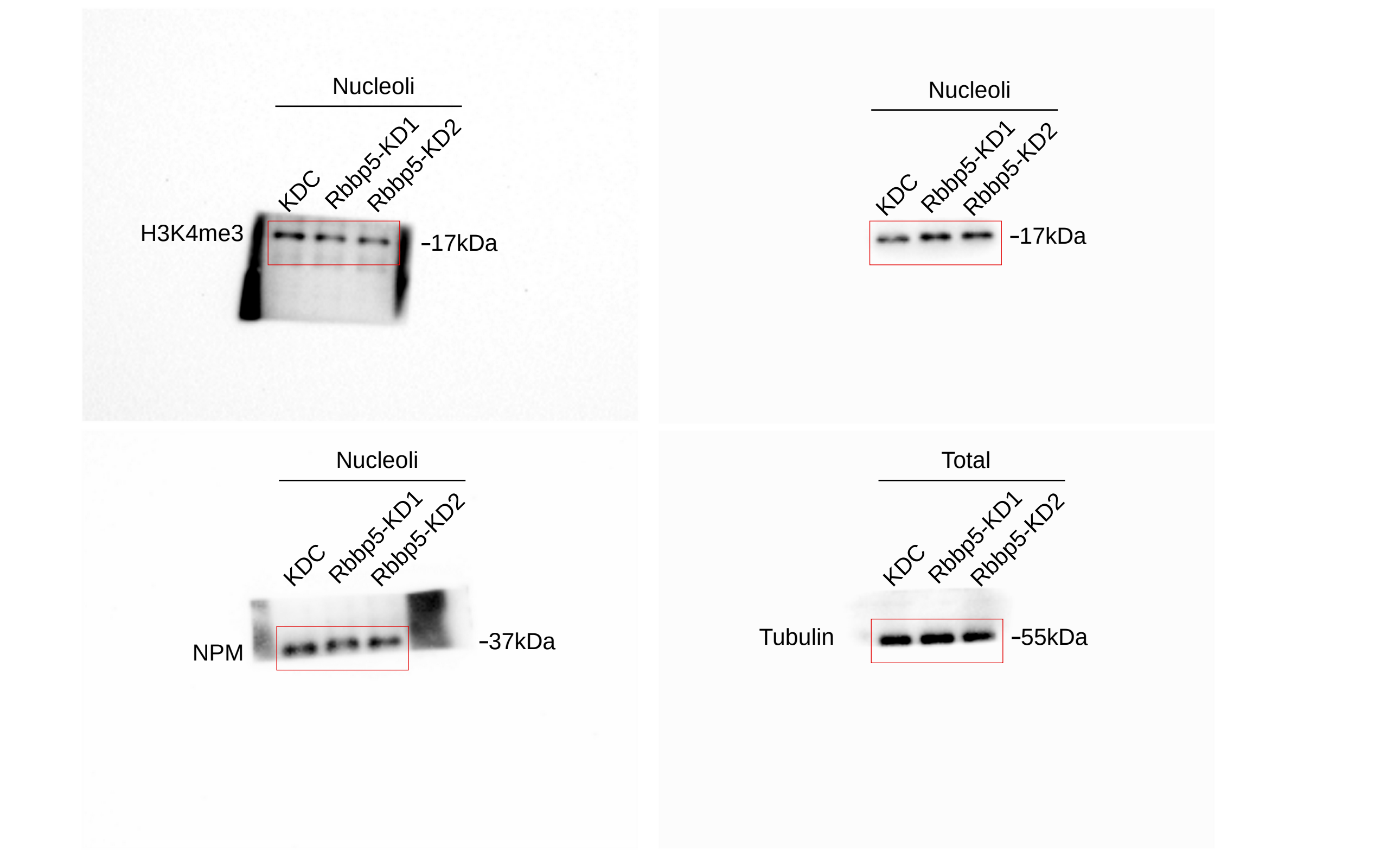

Figure 5F-H

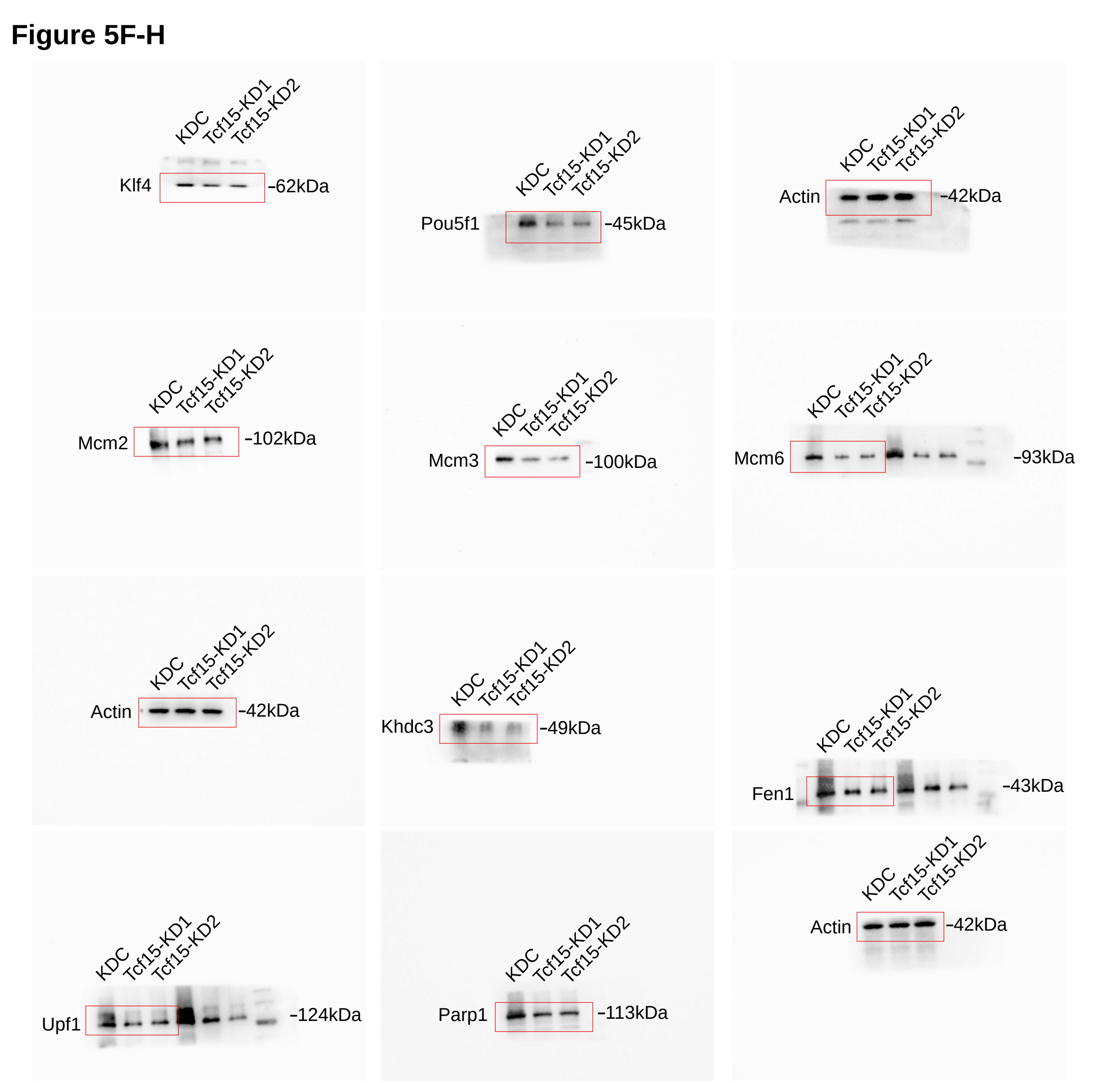

Figure 6B

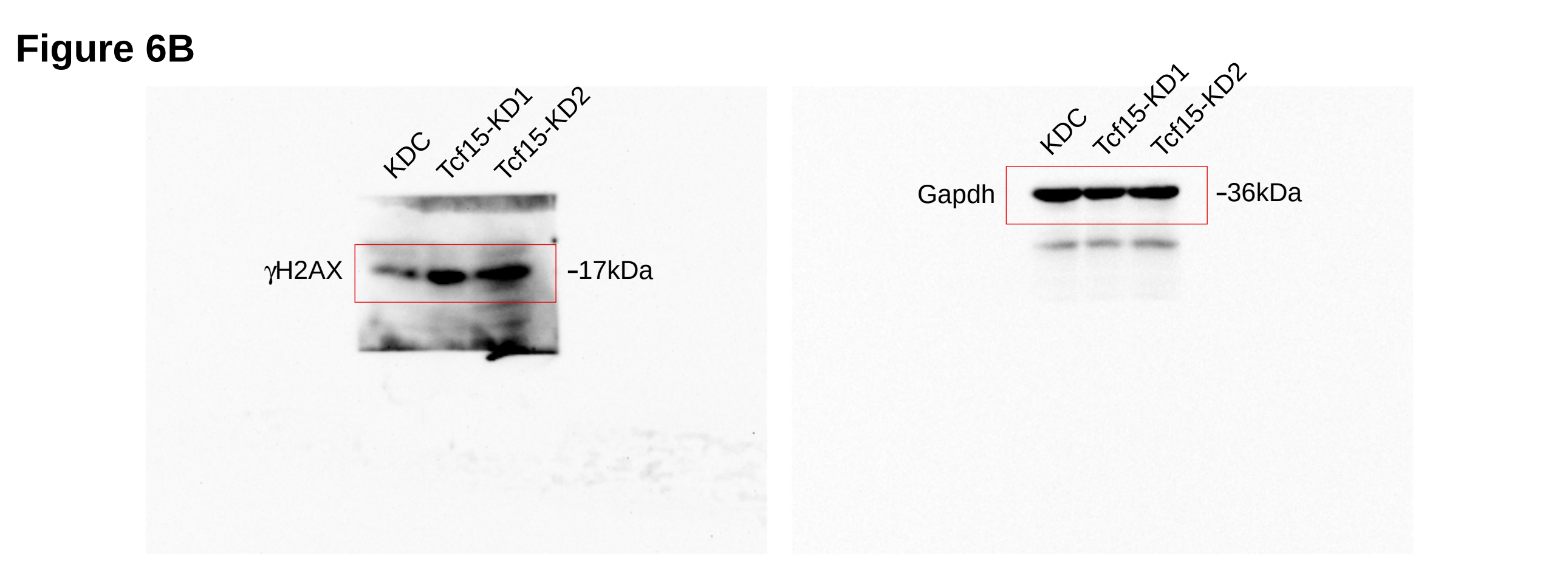

Figure 6F

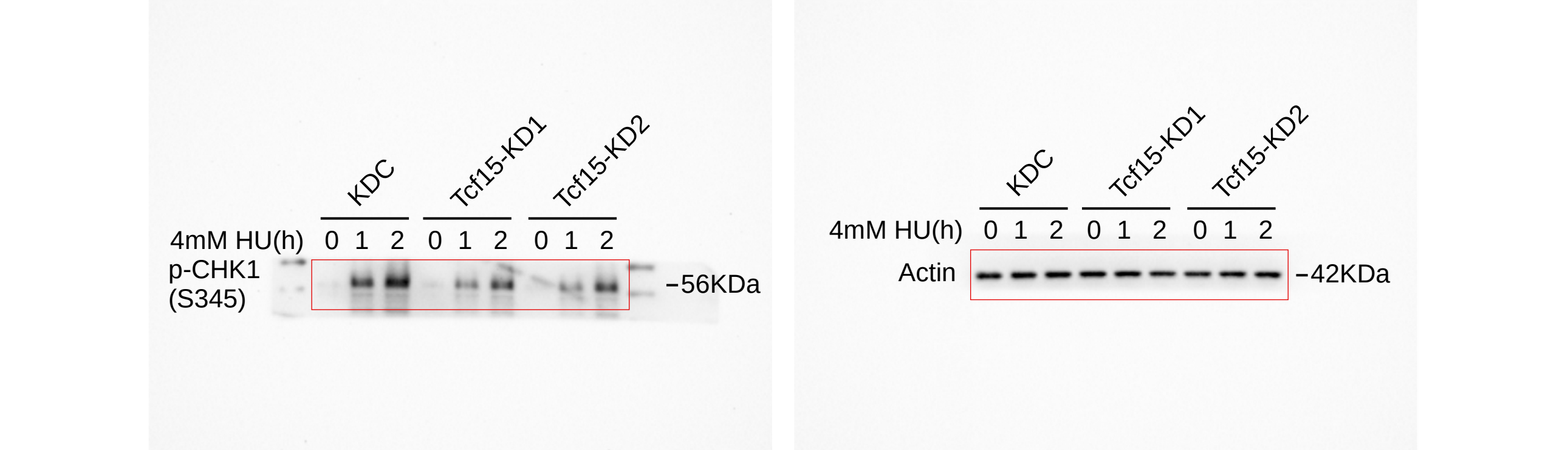

Figure 6J

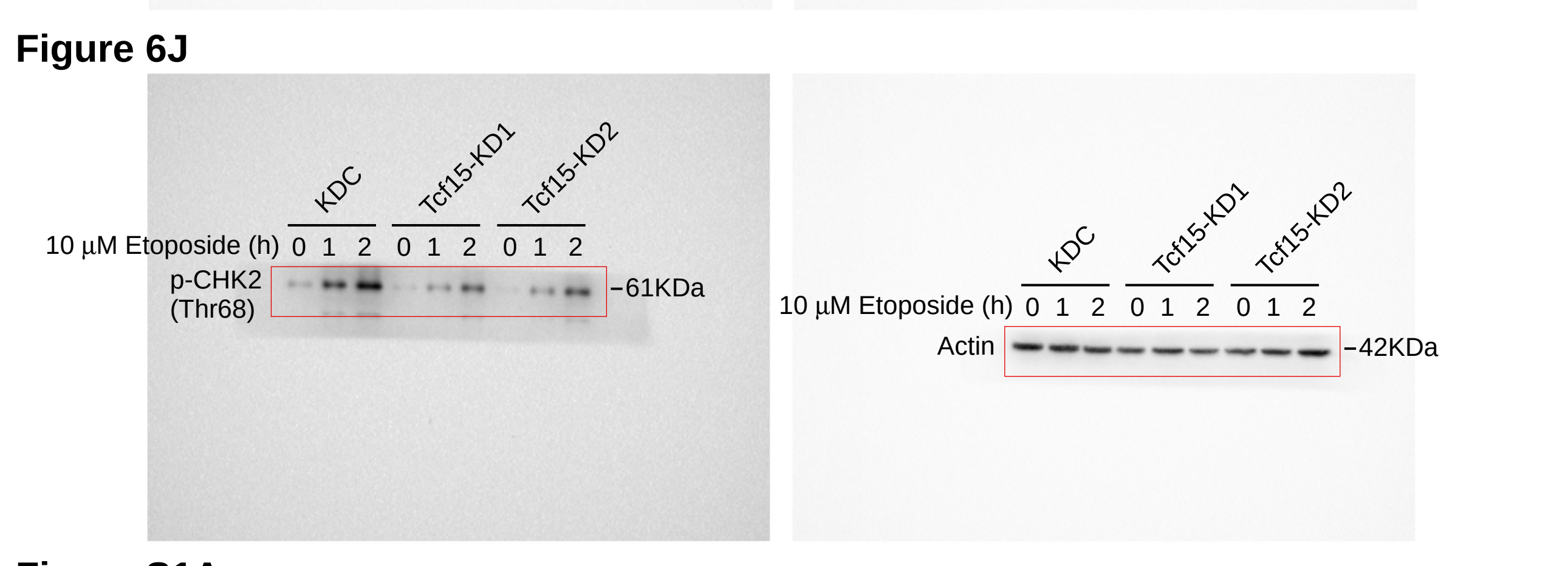

Figure S1A

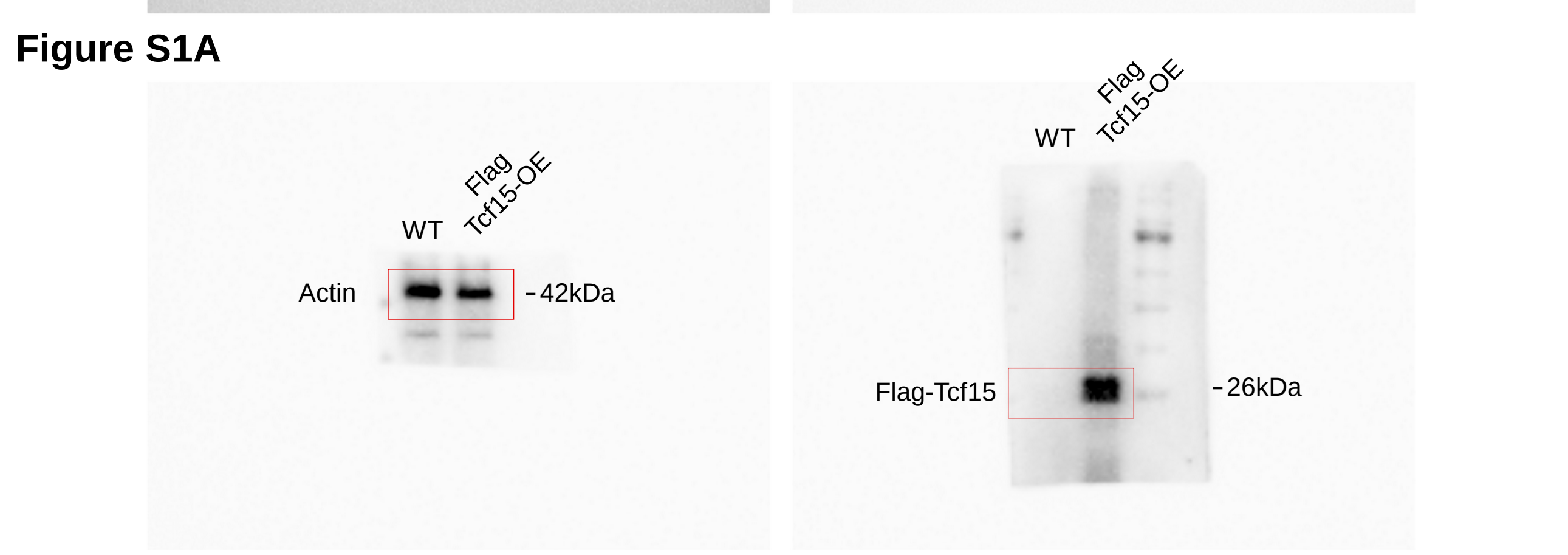

Figure S1C

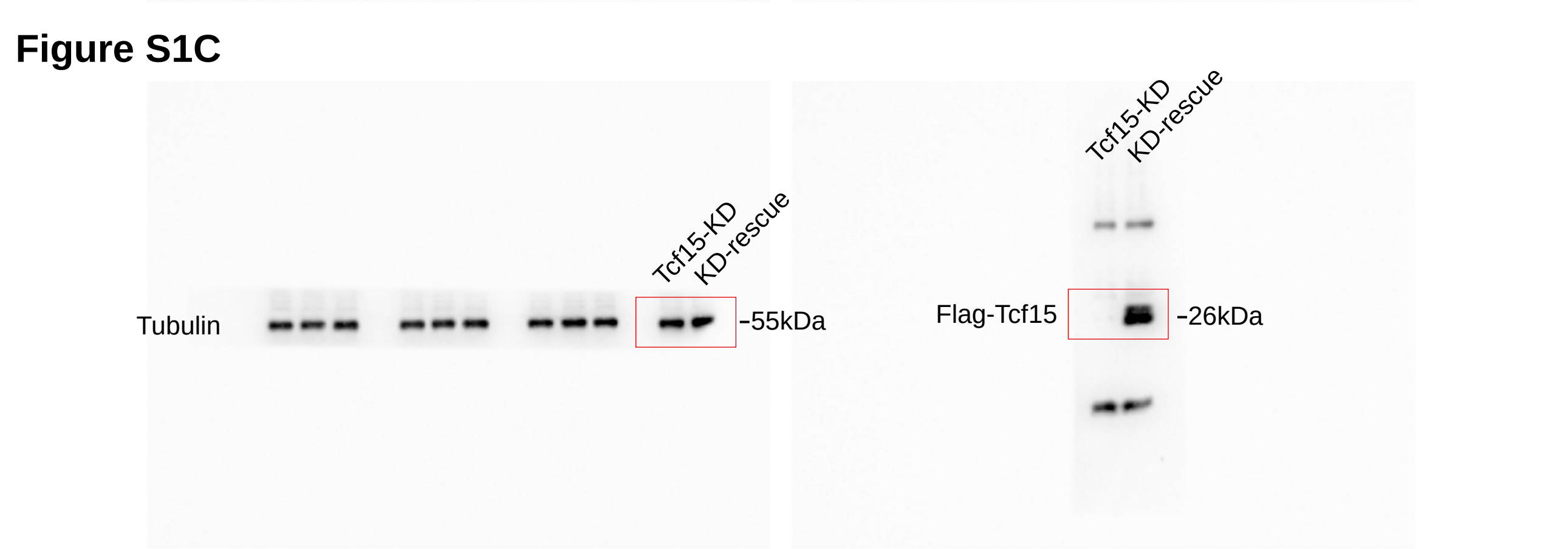

Figure S2D

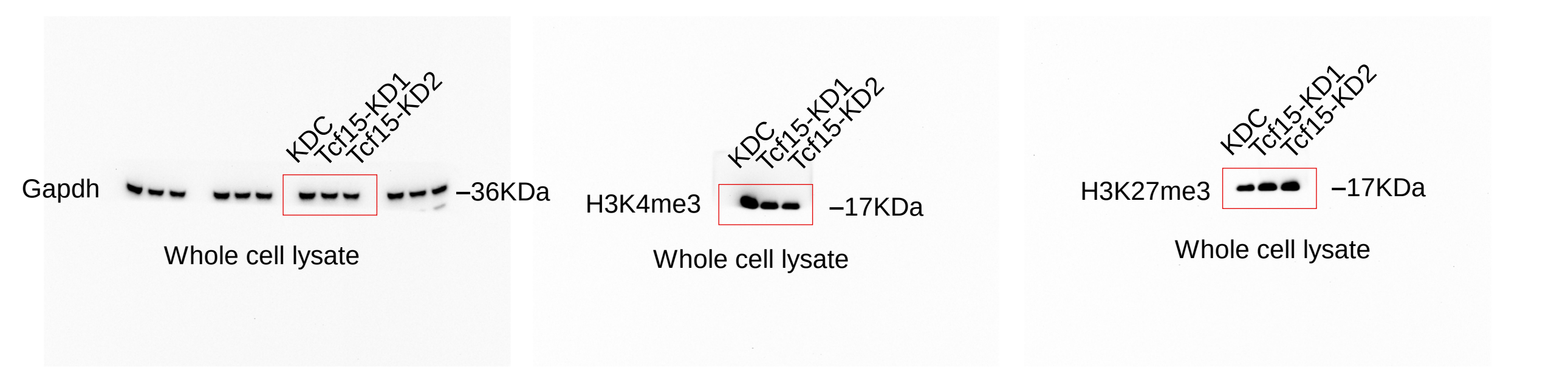

Figure S2E

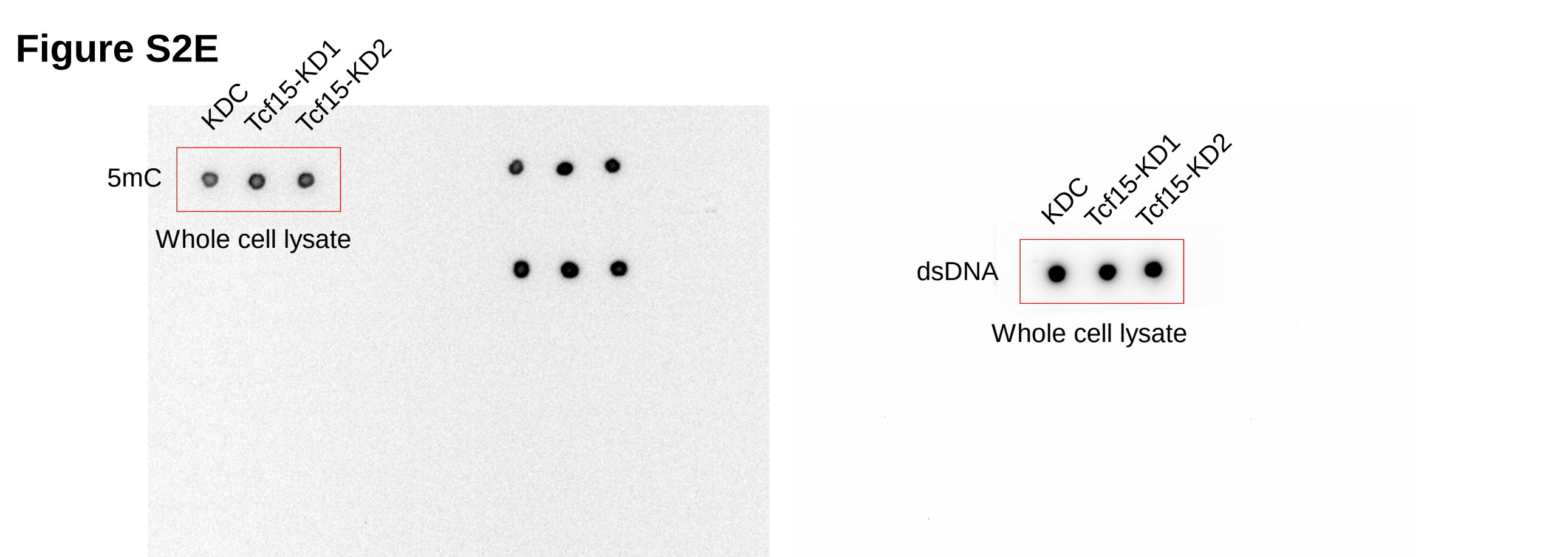

Figure S4A

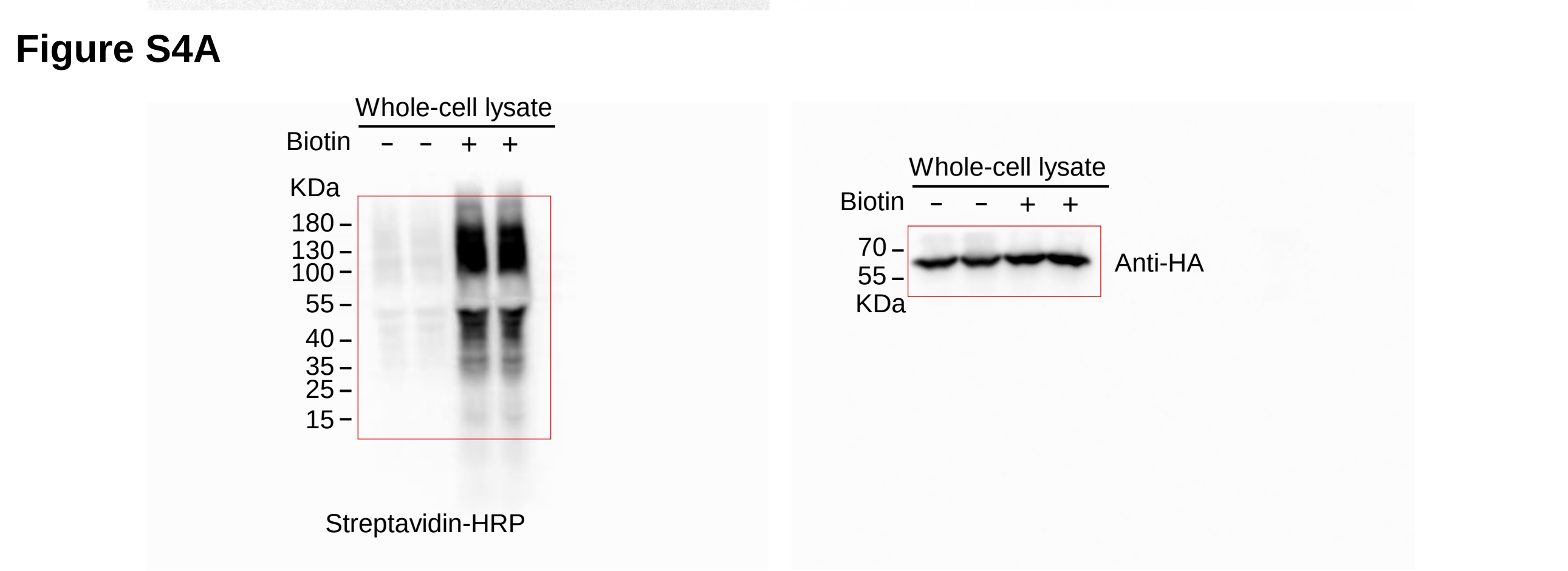

Figure S4C

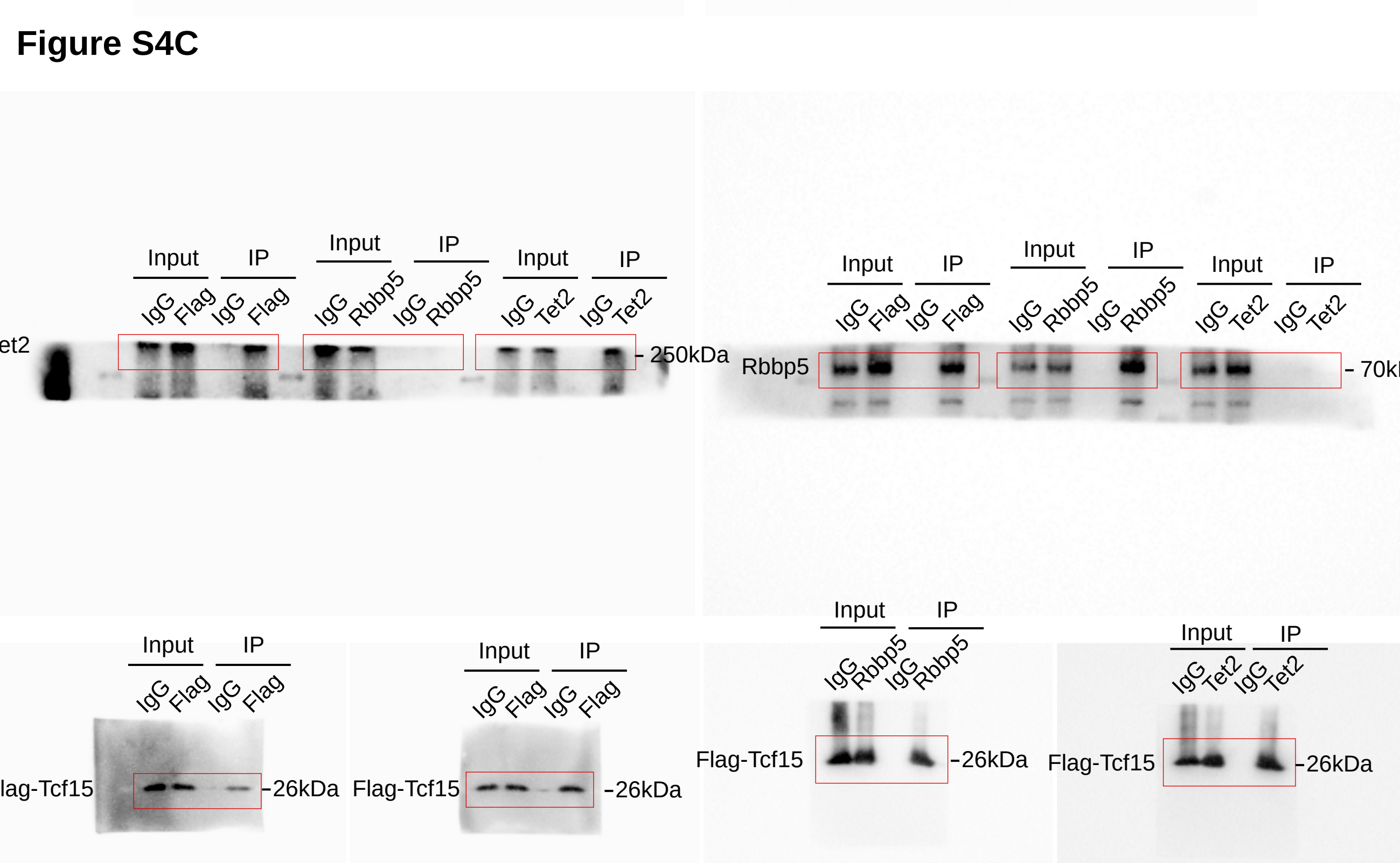

Figure S4D

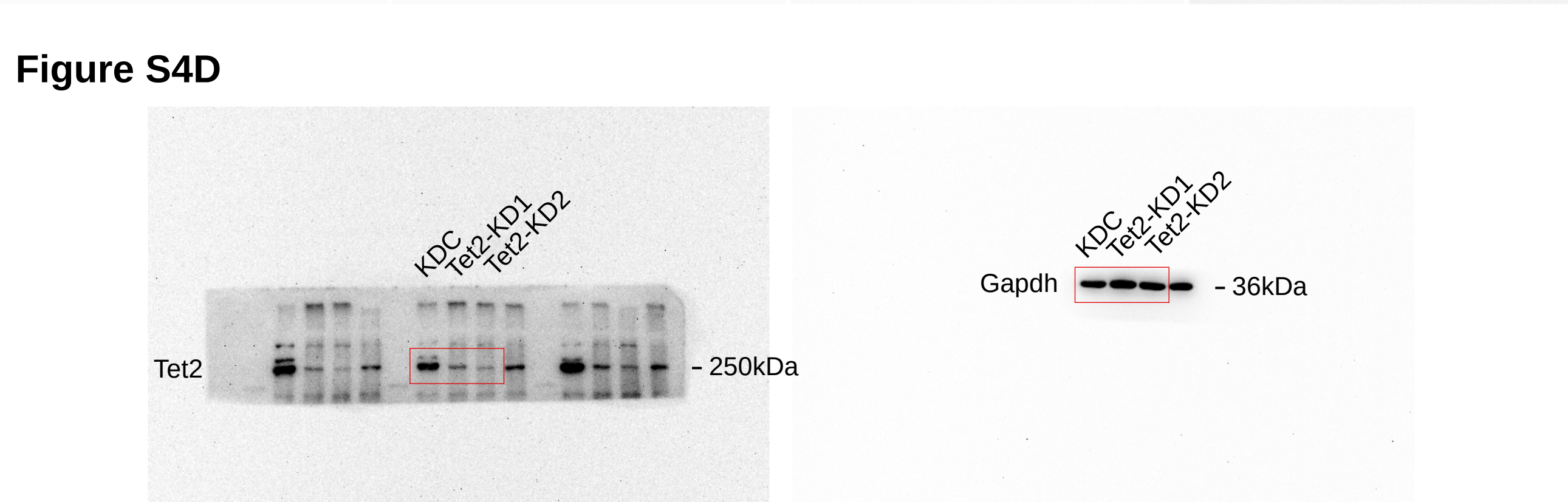

Figure S4E

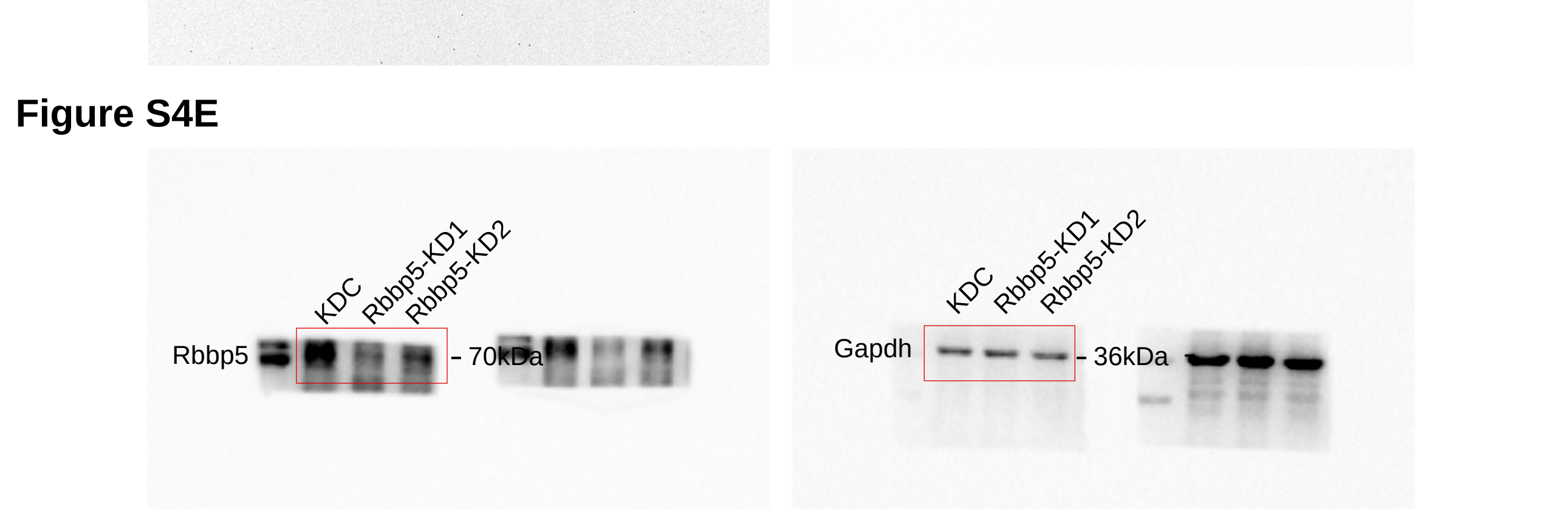

Figure S4(H-I)

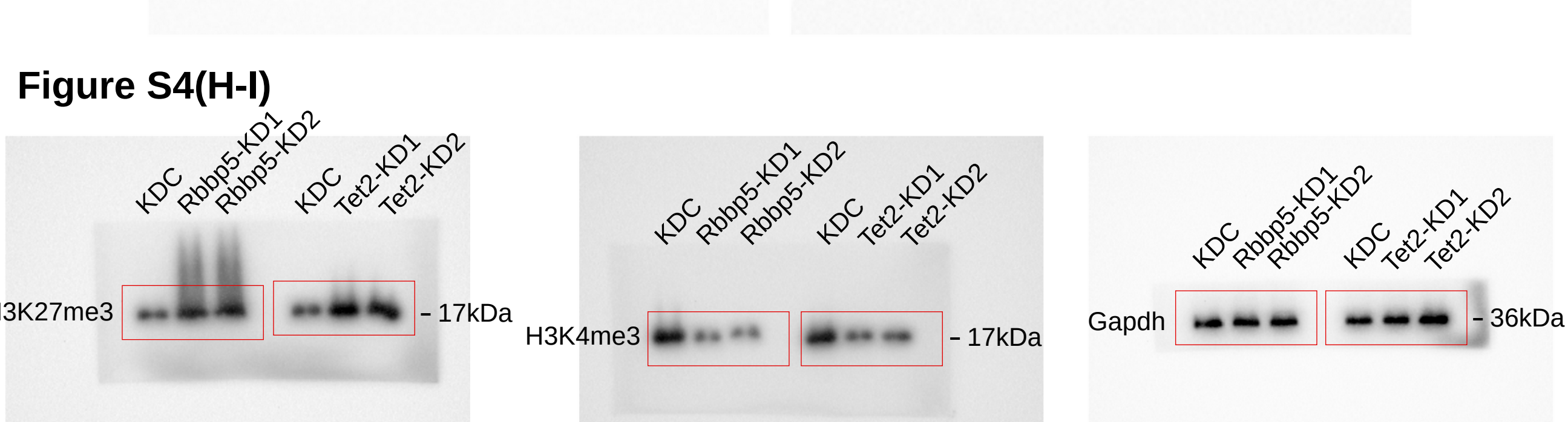

Figure S6A

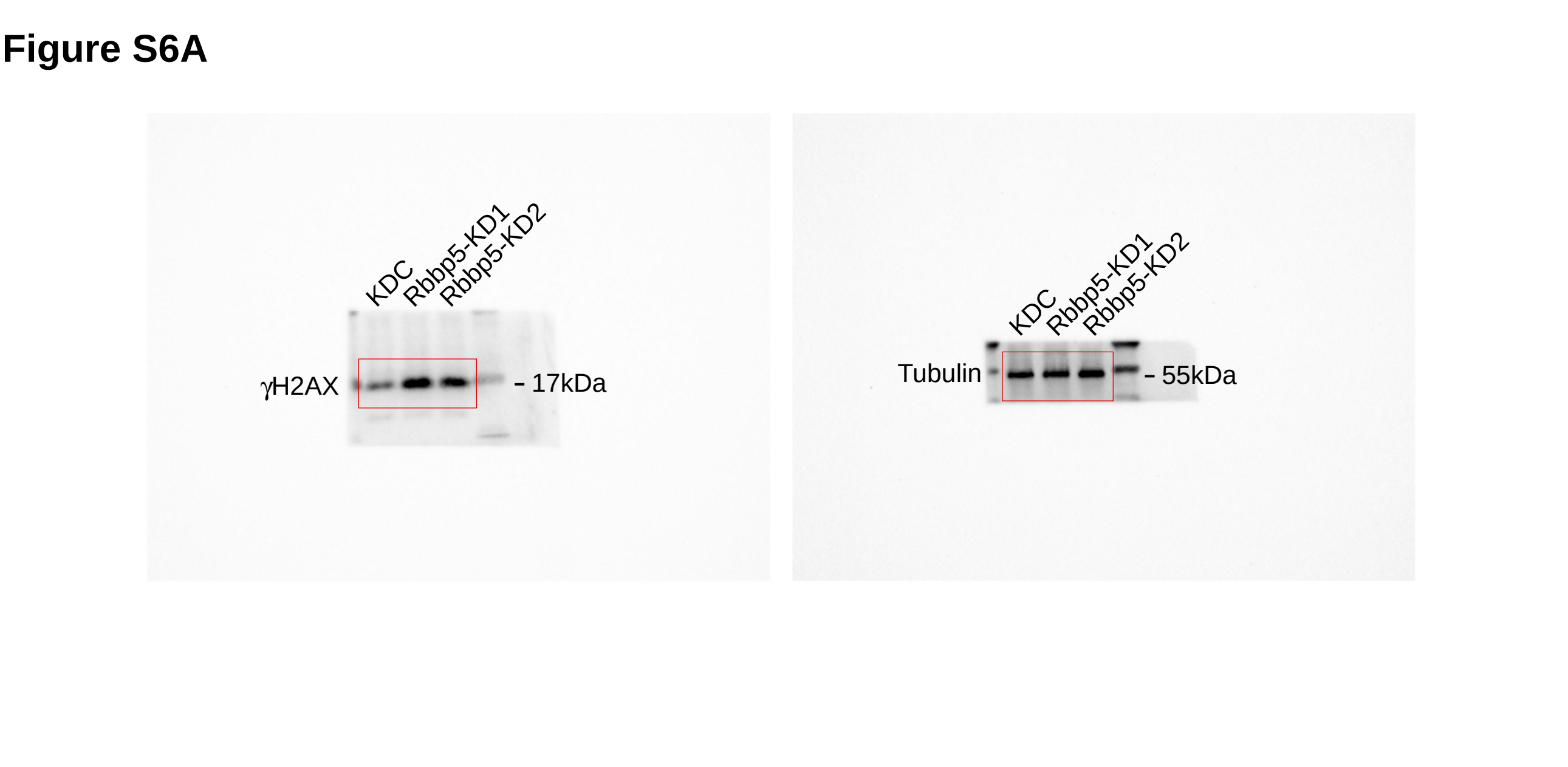

Supplement: Supplementary file 1 [file pbio.3003954.s017.pdf]
