# Supplementary material for: Tcf15 promotes an open nucleolar chromatin state to safeguard ribosome biogenesis and genome stability in mouse embryonic stem cells
Source: PLoS Biol. 2026 Aug 25;24(8):e3003954. doi: 10.1371/journal.pbio.3003954 (PMC13505954; doi:10.1371/journal.pbio.3003954)
Supplement: Supplementary file 2 [file pbio.3003954.s013.docx]

**S7 Table. Short hairpin RNA sequences used for gene knock down.**

| Gene | | | shRNA |
| --- | --- | --- | --- |
| shRNA sequences for pLKO.1 vector construction | | | |
| Negative  control  shRNA | Forward | 5'-CCGGTAAGGCTATGAAGAGATACCTCGAGGTATCTCTTCATAGCCTTATTTTTG-3' | |
|  | Reverse | 5'-AATTCAAAAATAAGGCTATGAAGAGATACCTCGAGGTATCTCTTCATAGCCTTA-3' | |
| Tcf15 shRNA1 | Forward | 5'-CCGGCGTGGACCGAAAGCTGTCTAACTCGAGTTAGACAGCTTTCGGTCCACGTTTTTG-3' | |
|  | Reverse | 5'-AATTCAAAAACGTGGACCGAAAGCTGTCTAACTCGAGTTAGACAGCTTTCGGTCCACG-3' | |
| Tcf15 shRNA2 | Forward | 5'-CCGGAGCTGGACAGAGGAGAAGATTCTCGAGAATCTTCTCCTCTGTCCAGCTTTTTTG-3' | |
|  | Reverse | 5'-AATTCAAAAAAGCTGGACAGAGGAGAAGATTCTCGAGAATCTTCTCCTCTGTCCAGCT-3' | |
| Tet2 shRNA1 | Forward | 5'-CCGGCCAACTCATGGGTCAATTCTTCTCGAGAAGAATTGACCCATGAGTTGGTTTTTTG-3' | |
|  | Reverse | 5'-AATTCAAAAACCAACTCATGGGTCAATTCTTCTCGAGAAGAATTGACCCATGAGTTGG-3' | |
| Tet2 shRNA2 | Forward | 5'-CCGGCGCTGGACATTTGTCTTGAAACTCGAGTTTCAAGACAAATGTCCAGCGTTTTTTG-3' | |
|  | Reverse | 5'-AATTCAAAAACGCTGGACATTTGTCTTGAAACTCGAGTTTCAAGACAAATGTCCAGCG-3' | |
| Rbbp5 shRNA1 | Forward | 5'-CCGGGCCTTCCTTTCAAGCAGAAGTCTCGAGACTTCTGCTTGAAAGGAAGGCTTTTTG -3' | |
|  | Reverse | 5'-AATTCAAAAAGCCTTCCTTTCAAGCAGAAGTCTCGAGACTTCTGCTTGAAAGGAAGGC -3' | |
| Rbbp5 shRNA2 | Forward | 5'-CCGGGCTCCATATTGTGTATAAAGGCTCGAGCCTTTATACACAATATGGAGCTTTTTG-3' | |
|  | Reverse | 5'-AATTCAAAAAGCTCCATATTGTGTATAAAGGCTCGAGCCTTTATACACAATATGGAGC-3' | |
| shRNA sequences for pTRIPZ vector construction | | | |
| Tcf15 KD-1 | 5'-TCTGTTGACAGTGAGCGAACGTGGACCGAAAGCTGTCTAAGTGAAGCCACAGATGTTAGACAGCTTTCGGTCCACGTCTGCCTACTGCCTCGGA-3' | | |
