# Supplementary material for: Tcf15 promotes an open nucleolar chromatin state to safeguard ribosome biogenesis and genome stability in mouse embryonic stem cells
Source: PLoS Biol. 2026 Aug 25;24(8):e3003954. doi: 10.1371/journal.pbio.3003954 (PMC13505954; doi:10.1371/journal.pbio.3003954)
Supplement: Supplementary file 3 [file pbio.3003954.s014.docx]

**S8 Table. Primers for PCR cloning and RT-PCR.**

| Gene | Oligonucleotides | |
| --- | --- | --- |
| RT-PCR primers | | |
| Tcf15 | Forward | 5'-GCCCGTGGACCGAAAGCTGT-3' |
|  | Reverse | 5'-CCCCCTTGCGCTGGTTGCTG-3' |
| Pre-47s | Forward | 5'-GTCCCGAACCTCCGCTCTTTT-3' |
|  | Reverse | 5'-ACAACGAGCGACTGCCACAAAA-3' |
| 5.8s | Forward | 5'-GACTCTTAGCGGTGGATCACT-3' |
|  | Reverse | 5'-CGATGATCAATGTGTCCTGCAA-3' |
| 18s | Forward | 5'-TGCGGGTCATAAGCTTGCGT-3' |
|  | Reverse | 5'-GGCGGGGCCGATCCGAG-3' |
| 28s | Forward | 5'-GTGTTGACGCGATGTGATTTCT-3' |
|  | Reverse | 5'-ATCCATTCATGCGCGTCACTA-3' |
| Gapdh | Forward | 5'-GTGATGGGTGTGAACCACGA-3' |
|  | Reverse | 5'-CATGAGCCCTTCCACAATGC-3' |
| Primers for ChIP-qPCR validation of Tcf15/Tet2/Rbbp5/EZH2/SUZ12 positive binding loci | | |
| 18s(14977-15061) | Forward | 5'-AGCTTGCGTTGATTAAGTCCC-3' |
|  | Reverse | 5'-CGATCCGAGGGCCTCACT-3' |
| 28s(21411-21607) | Forward | 5'-GCCTCACGATCCTTCTGACCTTT-3' |
|  | Reverse | 5'-CAGCTCACGTTCCCTATTAGTGG-3' |
| 28s(19692-19979) | Forward | 5'-CGAAAGGGAGTCGGGTTCAGAT-3' |
|  | Reverse | 5'-TGCTGCGGATATGGGTACGGC-3' |
| IGS(2394-2648) | Forward | 5'-CTGCCATCCACATGTCACCT-3' |
|  | Reverse | 5'-GGGCACCAGAACCTCGAATTA-3' |
| Negative control | Forward | 5'-GTGGTGCCAAATTTGTCTGTTA-3' |
|  | Reverse | 5'-ACATGAGACAACCTGCAGTAC-3' |
| RT-PCR Primers for rDNA CpG Methylation Validated | | |
| rDNA CpG | Forward | 5'-GACCAGTTGTTCCTTTGAGG-3' |
|  | Reverse | 5'-ACCTATCTCCAGGTCCAATAG-3' |
| rDNA CpG control | Forward | 5'-ACTGACACGCTGTCCTTTCC-3' |
|  | Reverse | 5'-GACAGCTTCAGGCACCGCGA-3' |
| Primers for 3xFlag-Tcf15 fragment insertion in the pTOMO-IRES-GFP vector | | |
| 3Flag-Tcf15 CDS | Forward | 5'-GCTCTAGAGCCACCATGGACTACAAGGACGACGATGACAAGGATTACAAAGATGACGACGATAAGGACTATAAGGACGATGATGATAAAGCTAGCGCGTTCGCGCTGCTGCG-3' |
|  | Reverse | 5'-CGCGGATCCTCATCGCCGAGGCCCTC-3' |
| Primers for construction of the pPB-CAG-Tcf15-3xHA-TurboID vector | | |
| Tcf15 CDS | Forward | 5'-ATTTTGGCAAAGAATTCGCGGCCGCGCCACCATGGCGTTCGCG-3' |
|  | Reverse | 5'-GAACATCATACGGGTACGCTCGCCGAGGCCCTCGGAGC-3' |
| 3xHA-TurboID | Forward | 5'-GCTCCGAGGGCCTCGGCGAGCGTACCCGTATGATGTTC-3' |
|  | Reverse | 5'-TCAAGCTTATCGAGCGGCCACGCGTTCAGTCGGCCCTGCTGAATT-3' |
