# Supplementary material for: Tcf15 promotes an open nucleolar chromatin state to safeguard ribosome biogenesis and genome stability in mouse embryonic stem cells
Source: PLoS Biol. 2026 Aug 25;24(8):e3003954. doi: 10.1371/journal.pbio.3003954 (PMC13505954; doi:10.1371/journal.pbio.3003954)
Supplement: Supplementary file 4 [file pbio.3003954.s015.docx]

S9 Table. Antibody information.

| Antibodies | Source | Dilution |
| --- | --- | --- |
| Flag Mouse monoclonal antibody | Proteintech; Cat#66008-4-Ig | 1:5000 (IB)  1:1000(IF)  1:100(ChIP) |
| Flag Rabbit monoclonal antibody | Cell Signaling Technology;  Cat#14793 | 1:1000(IF)  1:100(IP) |
| TET2 Rabbit polyclonal antibody | Proteintech; Cat#21207-1-AP | 1:500 (IB)  1:500(IF)  1:100(IP) |
| RBBP5 Rabbit polyclonal antibody | Novus Biologicals;  Cat#NB600-252 | 1:10000(IB) |
| Phospho-Histone H2A.X(Ser139) Mouse  mAb | Cell Signaling Technology;  Cat#80312 | 1:1000 (IB)  1:1000(IF) |
| P-CHK1(Ser345) Rabbit monoclonal  antibody | Cell Signaling Technology;  Cat#2348S | 1:1000 (IB) |
| P-CHK2(T68) Rabbit monoclonal antibody | Huabio; Cat#HA721633 | 1:2000 (IB) |
| β-Actin Mouse monoclonal antibody | Cell Signaling Technology;  Cat#3700 | 1:1000 (IB) |
| GAPDH Mouse monoclonal antibody | Proteintech; Cat#60004-1-Ig | 1:10000(IB) |
| alpha Tubulin Mouse monoclonal antibody | Invitrogen; Cat#62204 | 1:1000(IB) |
| Histone H3 Rabbit polyclonal antibody | Cell Signaling Technology;  Cat#9715 | 1:1000 (IB) |
| HA tag Rabbit polyclonal antibody | Proteintech; Cat#51064-2-AP | 1:5000 (IB) |
| NPM1 Mouse monoclonal antibody | sigma-Aldrich; Cat#B0556 | 1:250(IF)  1:1000 (IB) |
| FBL Mouse monoclonal antibody | Abcam; Cat#ab4566 | 1:1000(IB)  1:200(IF) |
| NCL Rabbit polyclonal antibody | Proteintech; Cat#10556-1-AP | 1:1000 (IB) |
| H3K27me3 Rabbit monoclonal antibody | Cell Signaling Technology;  Cat#9733 | 1:1000(IB)  1:1000(IF)  1:50(C&T) |
| H3K4me3 Rabbit monoclonal antibody | Cell Signaling Technology;  Cat#9751 | 1:1000(IB)  1:1000(IF) |
| Histone H3K9me3 Rabbit Polyclonal antibody | Thermo Fisher Scientific;  Cat#39062 | 1:1000(IF) |
| 5-mC Rabbit Monoclonal antibody | Cell Signaling Technology;  Cat#28692 | 1:1000(IF)  1:1000(DB) |
| dsDNA Mouse monoclonal antibody | Santa Cruz Biotechnology;  Cat#sc-58749 | 1:500 (DB) |
| Rabbit KLF4 antibody | Bioss; Cat#bsm-52850R | 1:1000(IB) |
| Rabbit POU5F1 antibody | Invitrogen; Cat#701756 | 1:500(IB) |
| MCM2 Rabbit Monoclonal antibody | Bioss; Cat#bsm-63311R | 1:1000(IB) |
| MCM3 Rabbit Monoclonal antibody | Abways; Cat#CY8637 | 1:1000(IB) |
| MCM6 Rabbit Monoclonal antibody | Abways; Cat#CY7363 | 1:1000(IB) |
| Rabbit KHDC3 in-house antibody | Made by Abmart | 1:500(IB) |
| FEN1 Rabbit Monoclonal antibody | Abways; Cat#CY6956 | 1:1000(IB) |
| UPF1 Rabbit Polyclonal antibody | Bioss; Cat#bs-8565R | 1:1000(IB) |
| PARP1 Mouse monoclonal antibody | Proteintech; Cat#66520-1-Ig | 1:1000(IB) |
| EZH2 Polyclonal antibody | Proteintech; Cat#21800-1-AP | 1:100(ChIP) |
| SUZ12 Rabbit Monoclonal Antibody | Cell Signaling Technology;  Cat#3737 | 1:100(ChIP) |
| Rat monoclonal anti-BrdU, also used for immunostaining of CIdU | Novus; Cat#NB500-169 | 1:1000 (IF) |
| Mouse monoclonal anti-BrdU, also used for immunostaining of IdU | BD Biosciences; Cat#347580 | 1:500 (IF) |
| Goat anti-Rabbit IgG (H+L) Secondary Antibody, Alexa Fluor 488 | Thermo Fisher Scientific; Cat#A-32731 | 1:500 (IF) |
| Goat anti-Mouse IgG (H+L) Secondary Antibody, Alexa Fluor 488 | Thermo Fisher Scientific; Cat#A-11029 | 1:500 (IF) |
| Goat anti-Rat IgG Secondary Antibody, Alexa Fluor Cy3 | Thermo Fisher Scientific; Cat#A-10522 | 1:500 (IF) |
| Goat anti-Mouse IgG (H+L) Secondary Antibody, Alexa Fluor 594 | Thermo Fisher Scientific; Cat#A-11032 | 1:500 (IF) |
| Goat anti-Rabbit IgG (H+L) Secondary Antibody, Alexa Fluor 594 | Thermo Fisher Scientific; Cat#A-11012 | 1:500 (IF) |
| Goat anti-Mouse IgG (H+L) Secondary Antibody, HRP | Thermo Fisher Scientific; Cat#31430 | 1:5000 (IB) |
| Goat anti-Rabbit IgG (H+L) Secondary Antibody, HRP | Thermo Fisher Scientific; Cat#31460 | 1:5000 (IB) |
