# Supplementary material for: Tcf15 promotes an open nucleolar chromatin state to safeguard ribosome biogenesis and genome stability in mouse embryonic stem cells
Source: PLoS Biol. 2026 Aug 25;24(8):e3003954. doi: 10.1371/journal.pbio.3003954 (PMC13505954; doi:10.1371/journal.pbio.3003954)
Supplement: Supplementary file 5 [file pbio.3003954.s021.docx]

**Point-by-point response to reviewers**

**Reviewer #1** (Lei Lei, identifies herself):

This manuscript by Dong et al. investigates the role of the transcription factor Tcf15 in maintaining an open nucleolar chromatin state in embryonic stem cells (ESCs). The authors report that Tcf15 localizes to the nucleolus, binds to the coding regions of rRNA genes, and recruits the epigenetic regulators Tet2 and Rbbp5. Through these interactions, Tcf15 suppresses DNA methylation and H3K27me3 modification while promoting H3K4me3 modification at rDNA loci. Loss of Tcf15 impairs ribosome biogenesis (RiBi), selectively compromises the translation of genome-maintenance factors, and leads to marked genome instability. Overall, this study identifies an underexplored function of Tcf15: sustaining a hyperactive chromatin state at rDNA loci to support robust ribosome biogenesis in ESCs. These findings are of significant value for advancing our understanding of nucleolar function and rRNA gene regulation in embryonic stem cells.

However, several aspects related to experimental design, data interpretation, and manuscript structure warrant improvement. Specific comments are as follows:

Major:

**1. Verification of Tcf15 binding to rDNA (Result 1)**

The authors employed immunofluorescence and nucleocytoplasmic separation to determine the nucleolar localization of Tcf15, and provided ChIP-seq and ChIP-qPCR data to demonstrate Tcf15 binding to rDNA. However, it is recommended to supplement with co-localization experiments using rDNA-FISH combined with Tcf15 immunofluorescence to more intuitively and comprehensively demonstrate the spatial binding relationship between Tcf15 and rDNA in the nucleolus, thereby strengthening the persuasiveness of the conclusions.

**Response**: We appreciate the reviewer’s suggestion. We agree that combining rDNA-FISH with Tcf15 immunofluorescence would provide additional evidence to support the conclusion that Tcf15 binds to rDNA. However, given that our ChIP-seq and ChIP-qPCR data (see Figs 1C and 1D) already sufficiently demonstrate Tcf15 binding to rDNA (Results section, page 8-9, lines 159–161), we believe that rDNA-FISH would primarily offer confirmatory rather than essential value, and we therefore elected not to pursue this experiment.

**2. Verification of rRNA stability (Result 4A)**

Result 4A shows decreased rRNA levels, but this phenotype may arise from two mechanisms: first, reduced rRNA transcription; second, increased degradation of 47S pre-rRNA. To exclude the influence of the latter mechanism, it is recommended to perform RNA half-life experiments using Actinomycin D (ActD) to clarify whether the decrease in rRNA is due to transcriptional inhibition, thereby interpreting the data more accurately.

**Response**: We thank the reviewer for the insightful comment. Consistent with the academic editor’s guidance, we did not carry out ActD-based RNA stability assays in this revision, with the following justifications:

Our multiple ChIP-seq and ChIP-qPCR data demonstrate that Tcf15 loss disrupts open chromatin architecture at rDNA loci, reduces activating histone marks H3K4me3 and elevates repressive modifications (DNA methylation, H3K27me3) on rDNA coding regions (Fig 2A–2E, 2H, and 2I; Fig S2A, S2B, S2D, and S2E; Fig S3B and S3C; Results section, pages 10–13, lines 187–252). These epigenetic changes directly point to suppressed rDNA transcription as the primary driver of lowered rRNA levels.

**3. Questions regarding nascent protein localization (Result 4C)**

Protein synthesis typically occurs primarily in the cytoplasm; however, Result 4C shows that nascent proteins are more enriched at the nucleolus. This phenomenon differs from classical understanding, and the authors are advised to explain this: Does this reflect specific translational regulatory mechanisms within the nucleolus? Or is it due to the particularity of experimental conditions? Adding relevant discussion will help readers understand the biological significance of this result.

**Response:** We thank the reviewer for this important and insightful comment. We agree that the observed enrichment of nascent proteins at the nucleolus warrants clarification. We have added a discussion of this point in the revised manuscript (page 17-18, line 349-357):

*(For your convenience, the revised content is provided below).*

“Notably, using the highly sensitive OPP assay, we also detected nascent protein synthesis in the nucleolus. Although protein translation is conventionally thought to occur exclusively in the cytoplasm, accumulating evidences supports that translation can also occur within the nucleus and nucleolus (PMID: 25110029). This localized translation is proposed to enable the rapid, on-demand synthesis of proteins essential for nucleolar functions, allowing for immediate responses to nucleolar stress or fluctuating functional demands. Our detection of nucleolar translation in ESCs is consistent with this emerging model, reflecting the high metabolic and synthetic activity characteristic of the nucleolus in pluripotent stem cells.”

**4. Supplementary experiments on the impact of Tcf15 on ESC pluripotency.**

This study focuses on the molecular function of Tcf15 in the nucleolus, revealing its effects on rRNA gene epigenetic regulation and ribosome biogenesis. Considering that ribosome biogenesis and nucleolar function are closely related to embryonic stem cell self-renewal and differentiation potential, the authors are recommended to supplement experiments on the effects of Tcf15 knockdown on mouse embryonic stem cell pluripotency maintenance and differentiation capacity, such as AP staining, pluripotential markers expression and EB formation, etc.

**Response:** Thank you for your valuable suggestion to link Tcf15-mediated ribosome biogenesis control with ESC pluripotency. We have performed the proposed experiments and added corresponding data to revised supplementary figures.

Tcf15 KD did not affect alkaline phosphatase (AP) staining (revised S6D Fig.) or the expression of core pluripotency markers (revised S6E Fig.), indicating that Tcf15 is dispensable for steady-state pluripotency maintenance under standard LIF culture conditions.

In embryoid body (EB) differentiation assays, Tcf15-knockdown cells retained the capacity to initiate EB formation at comparable efficiency to controls (revised S6F Fig.); however, the resulting EBs were markedly smaller (revised S6G Fig.), consistent with a proliferation defect driven by impaired ribosome biogenesis rather than a failure to exit pluripotency. We have integrated these findings into the Results section (page 23, lines 480-487) of the revised manuscript:

*(Figure S6D-G in revised manuscript. For your convenience, the revised figures were shown below).*

D

E

F

G

Revised S6 Fig. Tcf15-Rbbp5 axis maintains genome stability independent of pluripotency.

(D) Alkaline phosphatase (AP) staining of KDC, Tcf15-KD1 and Tcf15-KD2 ESCs. Scale bar, 0.2 cm.

(E) qRT-PCR quantification of core pluripotency marker genes (Oct4, Nanog, Klf4, Sox2) in KDC, Tcf15-KD1 and Tcf15-KD2 cells.

(F) Quantification of EB formation efficiency.

(G) Representative bright-field images of embryoid bodies (EBs) derived from KDC, Tcf15-KD1 and Tcf15-KD2 cells at day 5, Scale bar, 50 μm. Distribution of day 5 EB diameters across groups; red lines indicate median values. At least 40 EBs per group were analyzed.

**5. Supplementary verification of the Tcf15-Tet2/Rbbp5 signaling pathway.**

This manuscript describes the interaction between Tcf15 and Tet2/Rbbp5 and their effects on rRNA transcription and ribosome biogenesis. To further complete the interpretation of this signaling pathway, the following experiments are recommended:

**1) Detect the effects of manipulating Tet2 or Rbbp5 (such as knockdown/overexpression) on rRNA transcription and ribosome biogenesis.**

**Response:** We appreciate the reviewers' constructive suggestions. The nucleolus is a multifunctional cellular compartment in which an open chromatin configuration supports both canonical ribosome biogenesis and non-canonical roles, particularly the maintenance of three-dimensional genome architecture (PMID: 32976768). To functionally dissect the two Tcf15-associated axes, we independently knocked down Tet2 and Rbbp5 and examined their respective impacts on rRNA transcription and ribosome biogenesis. Depletion of Tet2 did not significantly affect steady-state pre-rRNA levels (revised Fig 4E) or global ribosome biogenesis (revised Fig 4F). By contrast, Rbbp5 knockdown recapitulated the reduction in pre-rRNA levels and the impairment of ribosome biogenesis observed in Tcf15-deficient cells (revised Figs 4G–H). Collectively, these results demonstrate distinct roles for the Tcf15-mediated open nucleolar chromatin configuration: the Tcf15-Rbbp5 complex is essential for rRNA transcription and ribosome production, whereas the Tcf15-Tet2 complex is dispensable for these processes. We have added these findings to the Results section (page 17-18, lines 358–363) and further discussed the mechanistic basis and functional implications of this divergence in the Discussion section (page 24-25, lines 513-522) of our revised manuscript.

*(Figure 4E-H in revised manuscript. For your convenience, please also see the figure below).*

F

E

G

H

Revised Fig 4. The Tcf15-Rbbp5 complex maintains active rRNA transcription, ribosome biogenesis, and nascent protein synthesis.

(E) qRT-PCR showed the relative levels of pre-47S, mature 28S, 18S, and 5.8S rRNA species in Tet2 KD and KDC ESCs. Gapdh was used as an internal control.

(F) Sucrose gradient fractionation followed by the A₂₅₄nm absorption analysis revealed no significant change in the abundance of ribosome subunits (40S and 60S), monosome (80S) and polysomes in Tet2 KD ESCs versus KDC.

(G) qRT-PCR showed the relative levels of pre-47S, mature 28S, 18S, and 5.8S rRNA species in Rbbp5 KD and KDC ESCs. Gapdh was used as an internal control.

(H) Sucrose gradient fractionation followed by the A₂₅₄nm absorption analysis revealed significant change in the abundance of ribosome subunits (40S and 60S), monosome (80S) and polysomes in Rbbp5 KD ESCs versus KDC.

*(For your convenience, the revised content is provided below).*

page 17-18, lines 358–363:

“To delineate the distinct roles of Tet2 and Rbbp5 in rRNA biogenesis, we performed individual knockdowns and assessed pre‑rRNA transcription and ribosome assembly. This approach revealed a marked functional asymmetry. Loss of Tet2 did not appreciably affect 47S pre‑rRNA abundance (Fig 4E) or overall ribosome biogenesis (Fig 4F). In contrast, Rbbp5 deficiency led to substantial reductions in both pre‑rRNA transcription (Fig 4G) and ribosome production (Fig 4H), mirroring the phenotype observed upon Tcf15 depletion.”

page 24-25, lines 513-522:

“Although Tet2 and Rbbp5 interact with Tcf15, both do not co‑immunoprecipitate with each other, suggesting that Tcf15 assembles them into physically distinct complexes: one containing Rbbp5 (a core subunit of the H3K4 methyltransferase complex) and the other containing Tet2 (a DNA dioxygenase). Through these parallel pathways, Tcf15 establishes a hyperactive chromatin state in the nucleolus. Functional dissection further reveals that the Tcf15‑Rbbp5 complex‑mediated active chromatin structure plays a canonical role in ensuring rRNA transcription and RiBi. In contrast, the Tcf15‑Tet2 complex‑mediated rDNA hypomethylation is dispensable for rRNA transcription and RiBi; whether it participates in non‑canonical functions, such as genome structure organization, warrants further investigation.”

**2) Compare whether the phenotypes after manipulating Tet2/Rbbp5 are consistent with those after manipulating Tcf15.**

If the results are consistent, it would more strongly support the molecular mechanism that "Tcf15 regulates nucleolar function through Tet2/Rbbp5," enhancing the logical completeness of the manuscript.

**Response：**Thanks for the suggestions. To distinguish the functional contributions of the two Tcf15-associated axes, we independently knocked down Rbbp5 and Tet2 and systematically compared their phenotypes with those of Tcf15 depletion. Knockdown of Rbbp5 recapitulated the key defects observed in Tcf15-deficient cells, including impaired pre-rRNA transcription, reduced ribosome biogenesis (revised Figs 4G–H), and increased DNA damage (revised S6A-C Figs.). These findings support a model in which the Tcf15–Rbbp5 axis serves as the primary pathway through which Tcf15 regulates rRNA synthesis, ribosome biogenesis, and genome stability. In contrast, Tet2 knockdown did not affect steady-state rRNA transcription or ribosome biogenesis (revised Figs 4E–F), clearly distinguishing its downstream effects from those of Rbbp5. We also observed elevated DNA damage in Tet2-knockdown ESCs(R1-R3 Figs, see below). However, we chose not to include these data in the revised manuscript, because the DNA damage phenotype in Tet2-depleted cells cannot be attributed to the same nucleolar ribosome biogenesis‑to‑genome stability cascade that we established for Tcf15 and Rbbp5, given that Tet2 depletion does not impair rRNA transcription or ribosome biogenesis. Moreover, Tet2 is broadly distributed throughout the nucleus, and achieving nucleolus‑restricted depletion of Tet2 remains technically challenging with current tools. We have further discussed the functional significance of Tet2 at rDNA in the Discussion section of our revised manuscript. All corresponding modifications are indicated in revised 4E–4H Figs and revised S6A–S6C Figs (Results section, page 18, lines 360–363, page 23, lines 475–478; Discussion section, page 25, lines 519-522).

*(Figs 4G-H and S6A-C Figs in revised manuscript. For your convenience, please also see the figure below).*

Revised Fig 4. The Tcf15-Rbbp5 complex maintains active rRNA transcription, ribosome biogenesis, and nascent protein synthesis.

(G) qRT-PCR showed the relative levels of pre-47S, mature 28S, 18S, and 5.8S rRNA species in Rbbp5 KD and KDC ESCs. Gapdh was used as an internal control.

(H) Sucrose gradient fractionation followed by the A₂₅₄nm absorption analysis revealed significant change in the abundance of ribosome subunits (40S and 60S), monosome (80S) and polysomes in Rbbp5 KD ESCs versus KDC.

Revised S6 Fig. Tcf15-Rbbp5 axis maintains genome stability independent of pluripotency.

(A) Immunoblotting and quantification of γH2AX protein levels in KDC, Rbbp5-KD1, Rbbp5-KD2 cells, with Tubulin as the loading control.

(B) Representative immunofluorescence staining for γH2AX (green) and DAPI (blue) in Rbbp5-knockdown cells, with corresponding quantitative analysis of mean fluorescence intensity. Scale bar, 10 μm.

(C) Representative images and quantitative tail moment analysis from neutral comet assay, showing increased DNA double-strand breaks in Rbbp5-KD ESCs. Scale bar, 50 μm.

R1-3 Figs for reviewers:

R2

R1

R3

(R1) Immunoblotting and quantification of γH2AX protein levels in KDC, Tet2-KD1, Tet2-KD2 cells, with Tubulin as the loading control.

(R2) Representative immunofluorescence staining for γH2AX (green) and DAPI (blue) in Tet2-knockdown cells, with corresponding quantitative analysis of mean fluorescence intensity. Scale bar, 10 μm.

(R3) Representative images and quantitative tail moment analysis from neutral comet assay, showing increased DNA double-strand breaks in Tet2-KD ESCs.

*(For your convenience, the revised contents are provided below).*

page 18, lines 360–363.

Loss of Tet2 did not appreciably affect 47S pre‑rRNA abundance (Fig 4E) or overall ribosome biogenesis (Fig 4F). In contrast, Rbbp5 deficiency led to substantial reductions in both pre‑rRNA transcription (Fig 4G) and ribosome production (Fig 4H), mirroring the phenotype observed upon Tcf15 depletion.

page 23, lines 475–478.

To further test this model, we asked whether Rbbp5 depletion—which disrupts RiBi—alone suffices to elicit DNA damage. Indeed, Rbbp5 KD increased γH2AX levels and DSBs, as detected by Western blot, immunofluorescence and neutral comet assays (S6A, B, C Figs.).

page 25, lines 519-522.

In contrast, the Tcf15‑Tet2 complex‑mediated rDNA hypomethylation is dispensable for rRNA transcription and RiBi; whether it participates in non‑canonical functions, such as genome structure organization, warrants further investigation.

**6. Optimization of the transition between Result 6 and preceding sections.**

The connection between Result 6 and the preceding sections is not sufficiently smooth. The preceding sections mainly describe Tcf15's effects on rDNA epigenetic regulation, rRNA transcription, and ribosome biogenesis—these factors all significantly influence nucleolar activity, while the nucleolus is a key factor in maintaining genome stability.

It is recommended that the authors add transitional content before Result 6: explicitly stating that Tcf15 may affect genome stability by regulating nucleolar structure and rRNA production, before then elaborating on the specific results of Tcf15's effects on genome stability (such as DNA damage, chromosomal instability, etc.). This would make the manuscript logic smoother and help readers understand the inferential chain from nucleolar function to genome stability.

**Response:** We thank the reviewer for this valuable suggestion. Following the recommendation, we have added a transitional paragraph at the beginning of Results section 6 (page 22, lines 451–457):

*(For your convenience, the revised content is provided below).*

“Our findings establish that the Tcf15–Rbbp5 complex supports active rRNA transcription and ribosome biogenesis. Ribo‑seq further showed that Tcf15 knockdown selectively compromises translation of proteins governing DNA replication and repair (Fig 5H), including Parp1, a versatile DNA repair factor [37], and Khdc3 (also called Filia), an ESC‑specific regulator of replication stress and damage repair [32-34]. This translational defect in genome maintenance factors prompted us to examine whether Tcf15 loss triggers genomic instability.”

**Minor:**

1. In Figure 3, the authors only assess co-localization between Tet2 and Tcf15. To strengthen the proposed recruitment model and maintain internal consistency, the authors should also perform immunofluorescence analyses to examine co-localization between Rbbp5 and Tcf15.

**Response:** We thank the reviewer for this suggestion. We have performed immunofluorescence co‑staining of Rbbp5 and FLAG‑Tcf15 and incorporated the corresponding imaging data into revised Fig 3D. The new IF results reveal partial nucleolar co‑localization between Rbbp5 and Tcf15, which is consistent with our recruitment model (page 14, lines 273–275).

*(Figure 3D in revised manuscript. For your convenience, please also see the figure below).*

D

Revised Fig 3. Tcf15 exerts its regulatory effects on nucleolar chromatin architecture via its physical interactions with Tet2 and Rbbp5 within the nucleolus.

(D) Immunofluorescence staining of Rbbp5 (red) and Tcf15 (green) revealed partial co-localization. Scale bar,10 μm.

*(For your convenience, the revised content is provided below).*

“In addition, immunofluorescence staining revealed co‑localization of Tcf15 with Tet2 and Rbbp5 in the nucleolus (Fig 3C and 3D).”

2. Statistical reporting is generally sound, but the number of biological replicates should be stated consistently across all figures.

**Response:** We thank the reviewer for the careful review of our statistical reporting. We have systematically re-examined all main and supplementary figures and have uniformly added the exact number of biological replicates to each figure legend. In addition, all descriptions of replicate numbers in the main text have been revised to ensure full consistency with the figure legends.

3. Terminology and Abbreviation Standardization Issues

1) ESCs was not defined as "Embryonic Stem Cells" upon first appearance. It is recommended to add the full name.

**Response:** We thank the reviewer for this reminder. We have now introduced the full term “embryonic stem cells” with the abbreviation (ESCs) at its first occurrence in the manuscript, and have standardized all abbreviations throughout the text for consistency (page 3, lines 33).

2) The abbreviations RiBi (ribosome biogenesis) and rDNA (ribosomal DNA) appear multiple times throughout the manuscript.

**Response:** Thank you for your reminding. We have revised the manuscript to define both ribosomal DNA (rDNA) and ribosome biogenesis (RiBi) with their full names at their first respective appearances, and unified all abbreviations in the full text (page 3, lines 33-34).

3) Several abbreviations appear at inappropriate positions for their first usage. For example, rRNA abbreviation first appears in the Abstract rather than the Introduction section. The first appearance of pre-rRNAs abbreviation is also inappropriate. A thorough self-check of the entire manuscript is recommended.

**Response:** Thank you for your reminding. We have conducted a full text-wide inspection of all abbreviations. We redefined rRNA, pre-rRNAs and all other abbreviations with their complete full names at their proper first occurrence within the main text, and standardized abbreviation usage across the abstract, introduction and all result sections.

It is suggested to adjust the first appearance positions of these abbreviations to conform to academic standards, providing the full expression when first used in the Introduction.

**Response:** Thank you for your reminding. We have adjusted all abbreviations such as rRNA and pre-rRNAs to define their full names at their first occurrence within the Introduction section in compliance with academic formatting standards.

**Reviewer #2:**

This study proposes that Tcf15 is an ESC-specific regulator of rDNA chromatin state that maintains ribosome biogenesis (RiBi) and genome stability. The authors report that Tcf15 localizes to the nucleolus, recruits Tet2 and Rbbp5 to rRNA genes, prevents repressive chromatin formation, sustains rRNA transcription, and thereby preserves translation of DNA replication/repair factors and genome integrity. The topic is interesting because regulation of rDNA chromatin in pluripotent stem cells is important and incompletely understood. The experiments are extensive and technically diverse (ChIP-seq, CUT&Tag, WGBS, Ribo-seq, ribosome profiling, and DNA damage assays). However, the central mechanistic claim is currently not convincingly supported by causality, and several conclusions substantially overreach the presented data. In its current form, the manuscript reads more as a correlation chain: from Tcf15 KD to rDNA chromatin changes, reduced rRNA, translation changes,

and to genome instability. But the authors have not demonstrated that these events are mechanistically connected rather than parallel consequences of Tcf15 perturbation. Major conceptual and technical clarifications are required before publication in PLOS Biology.

**1**. The main claim is that genome instability results from defective ribosome biogenesis mediated by Tcf15. However, the evidence only shows association. But no causal rescue experiment connects these steps.

At present, Tcf15 could independently regulate nucleolar chromatin, replication programs, stress responses rather than acting through ribosome biogenesis.

**Response：**We thank the reviewer for this rigorous critique regarding the causal relationship between ribosome biogenesis defects and genome instability. We provide multiple lines of evidence to dissect the multifaceted mechanisms driving genomic lesions upon Tcf15 depletion:

First, integrative analysis of our ChIP‑seq and RNA‑seq datasets demonstrates that Tcf15 does not directly occupy or transcriptionally regulate genes involved in DNA replication and repair. This rules out a model in which Tcf15 maintains genome stability through direct transcriptional control of these programs.

Second, our ribosome profiling data establish a linear translational cascade downstream of Tcf15 loss: Tcf15 knockdown represses pre‑rRNA transcription and impairs ribosome biogenesis, which specifically reduces the translational efficiency of mRNAs encoding core DNA replication and repair factors. The insufficient supply of these critical proteins consequently compromises the cell’s genome maintenance capacity.

Third, we provide genetic validation from an independent perspective: Rbbp5 knockdown recapitulates both the ribosome biogenesis defect (revised Figs 4G-H) and the DNA damage phenotype observed in Tcf15‑deficient cells (revised S6A-C Figs.). This demonstrates that disruption of the Tcf15–Rbbp5 axis alone is sufficient to reproduce the genome instability seen upon Tcf15 loss. Collectively, the selective translational suppression of genome maintenance factors, the observed replication stress and repair defects, together with the genetic validation via Rbbp5 knockdown, provide convergent support for a mechanistic link between Tcf15‑dependent ribosome biogenesis and genome stability.

We fully agree that a direct rescue experiment—restoring rRNA transcription in Tcf15‑deficient cells and assessing whether genome instability is alleviated—would further strengthen the causal connection. We made sincere efforts toward this goal using two independent strategies. Unfortunately, neither approach succeeded. One approach involved supplementation with exogenous angiogenin (ANG), a reported activator of rRNA transcription(PMID: 24122807; PMID: 4888354 ; PMID: 15735021). We tested a broad concentration range (10–1000 ng/mL). At the tested concentrations, ANG did not produce a meaningful recovery of pre‑47S rRNA levels in Tcf15‑KD cells. At higher concentrations >250 ng/mL), we observed a paradoxical suppression of rRNA transcription. While the mechanistic basis of this suppression remains unclear, it is consistent with the possibility that elevated ANG levels may induce cellular stress rather than specifically activate rDNA transcription in this context. These results indicate that ANG supplementation alone is insufficient to overcome the transcriptional deficit caused by Tcf15 loss under our experimental conditions. A second strategy employed CRISPR activation (CRISPRa) to directly drive rDNA transcription. We designed three distinct sgRNAs targeting the 47S rRNA promoter, cloned them into the CORP‑GFP vector, and successfully produced lentiviral particles. However, we were unable to establish a stable mESC line with activated rDNA transcription. We suspect that the highly repetitive nature of the ~200–300 rDNA copies poses a considerable technical challenge, as CRISPRa‑mediated activation across this repetitive locus may not be feasible without disrupting nucleolar homeostasis or compromising cell viability.

We also acknowledge that ribosomes are highly heterogeneous. In mouse and human genomes, ~ 200-300 copies of tandem rRNA genes from up to six chromosomes are clustered. Accumulating evidence indicates that ribosomes assembled from distinct rDNA repeats exhibit marked heterogeneity and exert preferential translational regulation on specific mRNA subsets (PMID: 40045785). Technically, it remains extremely challenging to restore physiological levels of rRNA transcription and ribosome biogenesis specifically from the same pool of endogenous rDNA repeats. For this reason, simple global activation of rRNA loci may fail to reverse the genome instability phenotype observed in Tcf15-knockdown embryonic stem cells. We hope that you can appreciate this limitation.

*(Figs 4G-H and S6A-C Figs in revised manuscript. For your convenience, please also see the figure below).*

Revised Fig 4. The Tcf15-Rbbp5 complex maintains active rRNA transcription, ribosome biogenesis, and nascent protein synthesis.

(G) qRT-PCR showed the relative levels of pre-47S, mature 28S, 18S, and 5.8S rRNA species in Rbbp5 KD and KDC ESCs. Gapdh was used as an internal control.

(H) Sucrose gradient fractionation followed by the A₂₅₄nm absorption analysis revealed significant change in the abundance of ribosome subunits (40S and 60S), monosome (80S) and polysomes in Rbbp5 KD ESCs versus KDC.

Revised S6 Fig. Tcf15-Rbbp5 axis maintains genome stability independent of pluripotency.

(A) Immunoblotting and quantification of γH2AX protein levels in KDC, Rbbp5-KD1, Rbbp5-KD2 cells, with Tubulin as the loading control.

(B) Representative immunofluorescence staining for γH2AX (green) and DAPI (blue) in Rbbp5-knockdown cells, with corresponding quantitative analysis of mean fluorescence intensity. Scale bar, 10 μm.

(C) Representative images and quantitative tail moment analysis from neutral comet assay, showing increased DNA double-strand breaks in Rbbp5-KD ESCs.

**2.** The manuscript emphasizes a nucleolus-specific function of TCF15, but the data do not exclude broader chromatin roles. My concerns are

1) ChIP-seq binding outside rDNA is not analyzed.2) Tet2 and Rbbp5 are global chromatin regulators. 3) Only rDNA effects are investigated.

The reviewer therefore cannot conclude Tcf15 is an rDNA-specific regulator rather than a general transcription or chromatin factor with a visible nucleolar pool.

**Response:** Thank you for your valuable suggestions. Upon further analysis, we identified merely 68 binding peaks outside rDNA regions (revised S1 Table), which predominantly localize to the distal regulatory regions of genes (revised S1I Fig.), and none of these binding sites overlapped with genes differentially expressed upon Tcf15 knockdown (see S5B Fig). Notably, these target genes show no functional relevance to the maintenance of genomic stability (see revised S1 Table). Collectively, our results indicate that Tcf15 is unlikely to function as a canonical transcription factor that drives target gene transcription; instead, Tcf15 primarily exerts its biological roles within the nucleus. We have added this analysis to the Results section (page 9, lines 161–165).

*(S1I Fig, S5B Fig and S1 Table in revised manuscript. For your convenience, please also see the figure below).*

I

Revised S1 Fig. Tcf15 is localized in the nucleolus of ESCs.

(**I**) Genomic distribution of non‑rDNA Tcf15 binding peaks identified by ChIP‑seq in Flag‑Tcf15 overexpressing ESCs.

Revised S1 Table. Tcf15 binding sites annotation.

Excel table. Columns list the genomic coordinate information and functional annotation of each Tcf15 binding site, including chromosome, peak start position, peak end position, regional genomic annotation, target gene ID, and distance from peak to gene transcription start site (TSS).

B

S5 Fig. Loss of Tcf15 impairs the translation of a specific subset of genes.

(B) Heatmap showing the differentially expressed genes in Tcf15 KD versus KDC ESCs.

*(For your convenience, the revised content is provided below).*

“To systematically explore Tcf15 binding beyond rDNA loci, we annotated all identified ChIP-seq peaks across the genome. Genomic distribution analysis demonstrated that only 68 additional binding peaks were detected in genomic regions outside of rDNA (S1 Table). These peaks were predominantly enriched at the distal regulatory regions of protein-coding genes (S1I Fig and S1 Table).”

3. Recruitment of Tet2 and Rbbp5 is not demonstrated (interaction ≠ recruitment)

The authors propose Tcf15 recruits Tet2 and Rbbp5 to rRNA genes by showing co-IP interaction and nucleolar co-localization (Tet2 only). This is a critical mechanistic gap. Without showing loss of Tet2/Rbbp5 binding at rDNA, the recruitment model is speculative.

**Response:** We thank the reviewer for the valuable suggestions. We performed ChIP‑qPCR assays in Tcf15‑knockdown cells, and the results revealed significantly reduced occupancy of both Tet2 and Rbbp5 at rDNA loci, supporting the model that Tcf15 recruits these proteins to rRNA genes (revised Figs 3E–F). We have added the following description to the Results section (page 14, lines 277–282):

“To test whether Tcf15 directly recruits Tet2 and Rbbp5 to rDNA, we performed ChIP‑qPCR with anti‑Tet2 and anti‑Rbbp5 antibodies in control and Tcf15‑KD ESCs (two independent knockdown lines). Binding of both factors to the 18S and 28S rDNA regions was significantly reduced following Tcf15 knockdown relative to controls (Figs 3E and 3F). These results support a model in which Tcf15 acts as an essential upstream scaffold that recruits both epigenetic regulators to rDNA.”

*(Figs 3E-F in revised manuscript. For your convenience, please also see the figure below).*

Revised Fig 3. Tcf15 exerts its regulatory effects on nucleolar chromatin architecture via its physical interactions with Tet2 and Rbbp5 within the nucleolus.

(E and F) ChIP‑qPCR analysis revealed that Tcf15 knockdown significantly reduced the binding of Tet2 and Rbbp5 to the 18S and 28S rDNA regions compared with control (KDC) cells.

4. The H3K27me3 interpretation is incomplete

A striking phenotype is increased H3K27me3 at rDNA. The authors interpret this as a consequence of reduced H3K4me3 antagonism. Crucially, the authors should determine whether known H3K27me3 regulators (e.g., nucleolar PRC2 localization mechanisms) are altered. Without this, the chromatin model is incomplete.

**Response:** Thank you for your valuable suggestions. To address the concern regarding altered PRC2 regulators at rDNA, we performed additional ChIP-qPCR assays targeting core PRC2 subunits EZH2 and SUZ12 in Tcf15-knockdown mESCs. Our new data demonstrate that depletion of Tcf15 leads to substantially elevated occupancy of EZH2 and SUZ12 across rDNA loci. We have integrated these PRC2 ChIP-qPCR into revised Figs 2F-G, and expanded the Results sections to build a complete two-branch chromatin model explaining the upregulation of repressive H3K27me3 marks (page 10-11, lines 203–209):

*(For your convenience, the revised content is provided below).*

“Consistent with the elevated H3K27me3 levels at rDNA in Tcf15 KD ESCs, we observed PRC2 occupancy, as confirmed by ChIP‑qPCR showing significant enrichment of its core subunits EZH2 and SUZ12 at rDNA loci relative to controls (Fig 2F and 2G). Collectively, these data indicate that Tcf15-mediated H3K4me3 maintenance antagonizes PRC2 recruitment and activity at rDNA, and that Tcf15 loss relieves this inhibition, leading to ectopic PRC2 deposition and subsequent H3K27me3 accumulation.”

We have also revised the Discussion section (page 26, lines 541–545) to incorporate this mechanistic insight:

*(For your convenience, the revised content is provided below).*

“Here, we demonstrate that Tcf15 promotes H3K4me3 deposition to counteract PRC2‑mediated H3K27me3 modification; loss of Tcf15 leads to aberrant PRC2 recruitment to rDNA. Thus, an active nucleolar chromatin state in stem cells must be safeguarded by multiple regulatory pathways, and future efforts should aim to identify additional mechanisms.”

*(Figs 2F-G in revised manuscript. For your convenience, please also see the figure below).*

Revised Fig 2. Tcf15 suppresses H3K27me3 modification and DNA methylation in rRNA genes of ESCs.

(F) ChIP-qPCR analysis showing increased binding of PRC2 core subunit EZH2 at rDNA regions in Tcf15-KD ESCs.

(G) ChIP-qPCR analysis showing increased binding of PRC2 core subunit SUZ12 at rDNA regions in Tcf15-KD ESCs.

5. The translation phenotype is internally inconsistent

The study reports severe reduction in rRNA transcription but nearly equal numbers of translationally up- and down-regulated genes. This contradicts a simple ribosome shortage model. An alternative explanation should be addressed because selective translation changes are a hallmark of stress signaling, not merely reduced ribosome abundance.

**Response：**Thank you for this thoughtful comment pointing out the apparent inconsistency between reduced rRNA synthesis and balanced bidirectional translational changes. We fully agree that this pattern cannot be explained by a simple model of universal translational suppression caused by limited ribosome supply.

Depletion of Tcf15 leads to diminished rRNA production, which blocks ribosome biogenesis at the ribosome assembly stage, rather than directly impairing the catalytic function of mature translational machineries. The resulting ribosomal deficiency does not uniformly repress global translation; instead, it triggers active compensatory and stress-specific translational reprogramming to counteract insufficient ribosome abundance, accompanied by activation of canonical stress cascades including the p53 signaling, apoptosis and senescence pathways (PMID: 35137207; PMID: 36651729). Consistent with this model, our translational profiling data clearly show significant enrichment of transcripts linked to p53 signaling, apoptosis and other compensatory stress response programs among translationally upregulated mRNAs in Tcf15-knockdown cells (revised Fig 5C and S5 Table).

We have revised the relevant Results and Discussion paragraphs to clarify this two-tier regulatory logic: impaired ribosome assembly initiates ribosomal stress, which drives selective translational remodeling instead of a non-specific global translation drop, accounting for the comparable numbers of translationally up- and down-regulated genes observed in our dataset (page 15, lines 411–415):

“By contrast, translationally activated transcripts are strongly enriched in p53 signaling and apoptotic pathways (Fig 5C and S5 Table), a typical ribosomal stress response. Indeed, defective ribosome biogenesis is known to elicit stress‑dependent translational reprogramming, favoring selective translation of stress‑response transcripts over global suppression (PMID: **35137207**; PMID: **36651729** ).”

We have also expanded the Discussion section (page 26-27, lines 553–565) to address this issue:

“Our integrated RNA‑seq and Ribo‑seq analyses show that Tcf15 KD-induced RiBi collapse does not cause a uniform decline in protein synthesis; rather, it rewires translational profiles. Specifically, many transcripts encoding DNA replication and repair factors—including Parp1, which plays multifaceted roles in DNA damage response and repair [42], and Khdc3 (also known as Filia, an ESC‑specific genome guardian) [32]—are disproportionately downregulated at the translational level, even though their mRNA levels remain unchanged upon Tcf15 depletion. This translational deficit coincides with decreased MCM protein abundance and reduced replication origin density, providing a direct mechanistic link between RiBi dysfunction and the DNA replication stress and genomic instability observed in Tcf15‑depleted ESCs. The concomitant upregulation of stress‑responsive factors further suggests that this represents an active, adaptive translational response to nucleolar stress rather than a passive consequence of ribosome scarcity.”

*(Figure 5C in revised manuscript. For your convenience, please also see the figure below).*

C

Revised Fig 5. Loss of Tcf15 impairs translation of a subset of ESC‑prevalent proteins.

(C) Gene ontology (GO) enrichment analysis revealed the top biological processes enriched from genes with increased translation efficiency in Tcf15 KD ESCs.

6. Over-interpretation of genome instability

The manuscript concludes Tcf15 "safeguards genome stability."

However, Tcf15 may directly regulates replication genes rather than through ribosome biogenesis.

Therefore the claim should be softened or mechanistically tested.

**Response：**Thank you for your comments.

To rule out the possibility that Tcf15 directly controls the transcription of replication-related genes, we integrated our genome-wide Tcf15 ChIP-seq and transcriptome RNA-seq datasets for systematic analysis. Our results show that Tcf15 exhibits negligible binding to the promoters of canonical DNA replication genes (please see revised S1 Table), and loss of Tcf15 does not significantly alter the steady-state mRNA levels of these genes at the transcriptional level (please see revised S5B Fig and S3 Table). Instead, our translational profiling data reveal that multiple core replication factors (including MCM3) display markedly reduced translational efficiency upon Tcf15 depletion, while their transcript abundance remains unchanged (please see revised Figs 5A–E, G and S4.2 Table). Collectively, these data demonstrate that Tcf15 does not sustain genome integrity via direct transcriptional regulation of replication machinery. Instead, Tcf15 safeguards genome stability indirectly by maintaining physiological ribosome biogenesis to support efficient translation of genome maintenance and replication proteins. Following the reviewer’s suggestion, we have softened absolute claims throughout the manuscript and clarified that ribosome biogenesis homeostasis serves as the primary functional axis by which Tcf15 protects genome stability.

*(S1 Table, Figs S5B,* *S3 Table, Figs 5A-E and S4.2 Table in revised manuscript. For your convenience, please also see the figure below).*

Revised S1 Table. Tcf15 binding sites annotation.

Excel table. Columns list the genomic coordinate information and functional annotation of each Tcf15 binding site, including chromosome, peak start position, peak end position, regional genomic annotation, target gene ID, and distance from peak to gene transcription start site (TSS).

B

S5 Fig. Loss of Tcf15 impairs the translation of a specific subset of genes.

(B) Heatmap showing the differentially expressed genes in Tcf15 KD versus KDC ESCs.

S3 Table. Differentially expressed genes between Tcf15 knockdown and control upon Tcf15 knockdown.

Excel table. First column gives gene names of Differentially expressed genes. Remaining columns give FPKM of Tcf15 knockdown control (KDC) repeat1, FPKM of KDC repeat2, FPKM of Tcf15 shRNA1 knockdown (KD1) repeat1, FPKM of KD1 repeat2, FPKM of Tcf15 shRNA2 knockdown (KD2) repeat1, and FPKM of KD2 repeat2.

Fig 5. Loss of Tcf15 impairs translation of a subset of ESC‑prevalent proteins.

(A) Volcano plot analysis of genes with translation affected by Tcf15 KD in ESCs. Blue dots represented genes showing decreased translation efficiency in Tcf15 KD ESCs, whereas red dots represented genes showing increased translation efficiency in Tcf15 KD cells.

(B) Gene ontology (GO) enrichment analysis revealed the top biological processes enriched from genes with decreased translation efficiency in Tcf15 KD ESCs.

(C) Gene ontology (GO) enrichment analysis revealed the top biological processes enriched from genes with increased translation efficiency in Tcf15 KD ESCs.

(D) Venn diagram showing overlap between the ESC-prevalent proteins and the proteins with translation decreased in Tcf15 KD ESCs.

(E) GO enrichment of the overlapped 181 proteins in (D).

(G) Western blot validated the decreased expression of several example proteins in Tcf15 KD ESCs when compared to KDC.

S4 Table. Genes with differentially translation efficiency in Tcf15 knockdown ESCs.

Genes with upregulated translation efficiency (TE) in Tcf15-knockdown ESCs are listed in Sheet 1. Conversely, genes with downregulated TE in Tcf15-knockdown ESCs are listed in Sheet 2, and the relevant GO enrichment results are presented in Sheet 3.

Minor concerns

1.Localization of Rbbp5 in the nucleolus is not shown by IF.

**Response：**Thank you for your suggestion. We have performed immunofluorescence staining of Rbbp5 together with TCF15 in 3FLAG-Tcf15 mESCs.

The newly acquired IF images, now presented in revised Fig 3D, clearly show that endogenous Rbbp5 distributes throughout the nucleoplasm, with a distinct subset of Rbbp5 signal overlapping with FLAG-TCF15 specifically within the nucleolar compartment under basal culture conditions (page 14, lines 273–275).

*(Fig 3D in revised manuscript. For your convenience, please also see the figure below).*

D

Revised Fig 3. Tcf15 exerts its regulatory effects on nucleolar chromatin architecture via its physical interactions with Tet2 and Rbbp5 within the nucleolus.

(D) Immunofluorescence staining of Rbbp5 (red) and Tcf15 (green) revealed partial co-localization. Scale bar,10 μm.

*(For your convenience, the revised content is provided below).*

“In addition, immunofluorescence staining revealed co‑localization of Tcf15 with Tet2 and Rbbp5 in the nucleolus (Fig 3C and 3D).”

2. Tet2/Rbbp5 localization after Tcf15 KD is missing.

Response: We thank the reviewer for this suggestion. To determine whether Tcf15 is required for the association of Tet2 and Rbbp5 with rDNA, we performed ChIP-qPCR assays targeting both proteins at rDNA loci in Tcf15-knockdown ESCs. Our results demonstrate that Tcf15 depletion significantly reduces the occupancy of both Tet2 and Rbbp5 at 18S and 28S rDNA regions compared with controls (revised Figs 3E and 3F). These data provide direct evidence that Tcf15 is required for the recruitment of Tet2 and Rbbp5 to rDNA chromatin. Notably, ChIP-qPCR directly measures the functional interaction of these epigenetic regulators with their target chromatin loci, which is the key mechanistic question underlying the reviewer's concern about localization. We have incorporated these findings into the Results section. All corresponding modifications are indicated in revised Figs 3E and 3F (Results section, page 14, lines 279–280).

*(Figs 3E-F in revised manuscript. For your convenience, please also see the figure below).*

Revised Fig 3. Tcf15 exerts its regulatory effects on nucleolar chromatin architecture via its physical interactions with Tet2 and Rbbp5 within the nucleolus.

(E and F) ChIP‑qPCR analysis revealed that Tcf15 knockdown significantly reduced the binding of Tet2 and Rbbp5 to the 18S and 28S rDNA regions compared with control (KDC) cells.

*(For your convenience, the revised content is provided below).*

“Binding of both factors to the 18S and 28S rDNA regions was significantly reduced following Tcf15 knockdown relative to controls (Fig 3E and 3F).”

3. Only knockdown is used; acute degradation would be more convincing.

**Response：**Thank you for the comments. We agree that rapid, acute depletion systems such as AID or dTAG could avoid adaptive compensatory responses triggered by long-term RNAi knockdown.

Unfortunately, multiple attempts to establish an inducible acute degradation system for Tcf15 have failed, largely owing to the unique coding sequence features of the Tcf15 locus that hinder reliable tagging without disrupting endogenous protein stability and function.

To compensate for the lack of an acute degradation model, we have validated all core phenotypes in two distinct doxycycline-inducible Tcf15 shRNA cell lines, which display consistent defects in nucleolar chromatin state（S3A–S3C Figs） as described in the manuscript (page 12, lines 240–248):

“To further confirm the direct role of Tcf15 on epigenetic regulations of rDNA loci, we attempted to insert the degron protein degradation cassette at C-terminus of Tcf15. Again, we failed to achieve precise insertion probably due to the DNA sequence issue. We therefore generated tet-on inducible shRNA KD ESCs. Doxycycline (Dox) treatment for 48 hours (h) caused ~70% decrease in Tcf15 mRNA expression (S3A Fig). Concordantly, Dox treatment for 48 h led to an increase in immunostaining intensity for nucleolar H3K27me3 (S3B Fig). Quantification of rDNA methylation by qPCR following HpaII digestion consistently showed the elevated methylation level in transient KD cells (S3C Fig). Thus, Tcf15 directly regulates the epigenetic modifications of rDNA loci.”

*(Figs S3A-C in revised manuscript. For your convenience, please also see the figure below).*

S3 Fig. Transient knockdown of Tcf15 increased H3K27me3 modification and DNA methylation in nucleoli.

(A) qRT-PCR validated the decreased mRNA expression of Tcf15 after doxycycline (Dox)-induced transient KD for 48 h. *Gapdh* served as an internal control.

(B) Immunofluorescence staining of H3K27me3 and NPM1 in transient Tcf15 KD ESCs. Note that H3K27me3 level was increased in the nucleolus. Scale bar, 10 μm.

(C) qPCR validated the increased level of CpG methylation in the nucleolus after transient KD of Tcf15 for 48 h.

4. Potential overexpression artifacts from Flag-Tcf15 remain a concern.

**Response：**Thank you for raising this critical concern regarding potential artifacts. To eliminate biases caused by supra-physiological protein abundance, we reconstituted FLAG-tagged Tcf15 in Tcf15-knockdown background cells. Quantitative qPCR validation confirms that the transcript level of exogenous FLAG-Tcf15 is comparable to the endogenous Tcf15 expression level observed in wild-type mESCs (please see S1E Fig.). This near-physiological expression setting largely avoids artificial phenotypes originating from protein overexpression. We have clarified this point in the Results section (page 8, lines 143–146):

“After confirming that the mRNA level of Tcf15 in KD-rescue ESCs was comparable to that in wild-type (WT) cells (S1E Fig), we re-examined the sub-cellular localization of Tcf15 in KD-rescue ESCs. Consistently, Tcf15 was reproducibly detected in the nucleolus of KD-rescue ESCs (S1F Fig).”

*(Fig S1E in revised manuscript. For your convenience, please also see the figure below).*

E

S1 Fig. Tcf15 is localized in the nucleolus of ESCs.

(E) qRT-PCR showed that WT and KD-rescue ESCs had comparable levels of Tcf15 mRNAs.

5. The manuscript does not examine pluripotency state changes.

**Response:** We appreciate this important comment reminding us to evaluate whether Tcf15 depletion alters mESC pluripotency. We have completed the full panel of pluripotency-related assays, including AP staining (revised S6D Fig.), qPCR quantification of core pluripotency markers (Oct4, Sox2, Nanog) (revised S6E Fig.), and embryoid body (EB) differentiation tests (revised S6F-G Fig.).

Under standard LIF-containing maintenance culture, Tcf15-knockdown colonies retain compact morphology and strong AP staining, with no significant downregulation of pluripotency transcripts, indicating intact basal self-renewal capacity. Nevertheless, EB formation assays reveal that while Tcf15-deficient cells can initiate differentiation, the resulting EBs are markedly smaller than control counterparts at matched time points. This indicates Tcf15 is dispensable for steady-state pluripotency maintenance but required for cell differentiation. All these data have been added to revised supplementary figures and integrated into the Results (page 23, lines 480-487).

*(Figure S6D-G in revised manuscript. For your convenience, the revised figures were shown below).*

D

E

F

G

Revised S6 Fig. Tcf15-Rbbp5 axis maintains genome stability independent of pluripotency.

(D) Alkaline phosphatase (AP) staining of KDC, Tcf15-KD1 and Tcf15-KD2 ESCs. Scale bar, 0.2 cm.

(E) qRT-PCR quantification of core pluripotency marker genes (Oct4, Nanog, Klf4, Sox2) in KDC, Tcf15-KD1 and Tcf15-KD2 cells.

(F) Quantification of EB formation efficiency.

(G) Representative bright-field images of embryoid bodies (EBs) derived from KDC, Tcf15-KD1 and Tcf15-KD2 cells at day 5, Scale bar, 50 μm. Distribution of day 5 EB diameters across groups; red lines indicate median values.

*(For your convenience, the revised content is provided below).*

“Given that Tcf15 KD partially reduced Oct4 and Klf4 protein levels (Fig 5F), we asked whether pluripotency itself was affected. Tcf15 KD did not affect alkaline phosphatase (AP) staining (S6D Fig), expression of core pluripotency markers (S6E Fig), or embryoid body (EB) formation efficiency (S6F Fig), although Tcf15‑KD EBs were smaller (S6C Fig), consistent with the proliferation defect caused by impaired RiBi. Thus, the defects we observed arise directly from the role of Tcf15 in rDNA chromatin regulation and rRNA transcription, rather than from compromised pluripotency. Thus, Tcf15 depletion did not cause compromised pluripotency. “

**Reviewer #3:**

This study identifies Tcf15 as a regulator of rDNA chromatin state and ribosome biogenesis in embryonic stem cells (ESCs). The authors show that Tcf15 localizes to the nucleolus, binds rRNA gene regions, and recruits epigenetic modifiers such as Tet2 and the H3K4 methyltransferase component Rbbp5 to maintain an active chromatin configuration at rDNA loci. Loss of Tcf15 increases repressive chromatin marks, reduces pre-rRNA transcription, and impairs ribosome biogenesis, leading to altered translation of mRNAs involved in DNA replication and repair. These defects ultimately cause genomic instability, revealing a previously unrecognized link between rDNA chromatin regulation, ribosome production, and genome maintenance in ESCs.

Overall, this is a really nice study and I only have minor suggestions:

- Fig. 3A - it is not clear where tet2. Rbbp5, and Tcf15 are on the plot. Also, some dots look orange. Is that on purpose and what does it signify?

**Response:** Thank you for your careful inspection of Figure 3A. The darker orange appearance of certain dots arises from overlapping data points. We have remade the figure and added clear labels to separately indicate the point of Tcf15, Tet2 and Rbbp5.

*(Fig 3A in revised manuscript. For your convenience, please also see the figure below).*

- Fig. 4D - can the authors put the flow cytometry data on log scale instead of semi-log and normalize the y-axis to %max.

**Response:** Thank you for suggestions. We have replotted the flow cytometry histograms on a full logarithmic scale and normalized the y-axis to %Max as requested in the revised Fig 4D.

*(Figure 4D in revised manuscript. For your convenience, please also see the figure below).*

D

Fig 4. The Tcf15-Rbbp5 complex maintains active rRNA transcription, ribosome biogenesis, and nascent protein synthesis.

(D) Flow cytometry analysis of OPP green fluorescence label in Tcf15 KDC and KD ESCs. Histograms are shown on a logarithmic scale with y-axis normalized to %Max. The relative fluorescence intensities are shown in the right panel. Equal starting cell numbers were loaded for each group during flow cytometry acquisition. The reduced cell events in Tcf15 KD samples reflect the decreased proportion of OPP-positive cells, consistent with the reduced nascent protein synthesis rate observed in (C). Ctrl, cells were not stained with OPP.

- Fig. 4D - Can the authors comment on why they have fewer cells for the knockdowns?

**Response:** Thank you for your careful inspection of Fig 4D. We loaded equal starting cell numbers for each group during flow cytometry acquisition. Only OPP-positive cells are visualized in Fig 4D, explaining the reduced cell events observed in Tcf15-knockdown samples. This clarification has been added to the figure legend (page 18-19, lines 380–386):

*(For your convenience, the revised content is provided below).*

“(D) Flow cytometry analysis of OPP green fluorescence label in Tcf15 KDC and KD ESCs. Histograms are shown on a logarithmic scale with y-axis normalized to %Max. The relative fluorescence intensities are shown in the right panel. Equal starting cell numbers were loaded for each group during flow cytometry acquisition. The reduced cell events in Tcf15 KD samples reflect the decreased proportion of OPP-positive cells, consistent with the reduced nascent protein synthesis rate observed in (C).”
