# Supplementary material for: Tcf15 promotes an open nucleolar chromatin state to safeguard ribosome biogenesis and genome stability in mouse embryonic stem cells
Source: PLoS Biol. 2026 Aug 25;24(8):e3003954. doi: 10.1371/journal.pbio.3003954 (PMC13505954; doi:10.1371/journal.pbio.3003954)
Supplement: Supplementary file 6 [file pbio.3003954.s022.docx]

**Point-by-point response to reviewers**

Reviewer remarks:

Reviewer #1: Through extensive and rigorous additional experiments, the authors have addressed the major mechanistic concerns raised in the first round of review. This study identifies Tcf15 as a novel regulator of rDNA chromatin state in stem cells and reveals a previously unrecognized link between ribosome biogenesis and genome stability, which is of significant scientific importance.

1.Discussion of Tet2 phenotype: The authors should briefly mention in the Discussion section the observation that Tet2 knockdown also causes DNA damage despite having no effect on rRNA transcription, and note that Tet2 may maintain genome stability through pathways independent of ribosome biogenesis.

Response: We thank the reviewer for this suggestion. In response, we have added the following statement in the Discussion section (page 25, lines 529–531):

“Notably, we also found that Tet2 depletion induces DNA damage without altering rRNA transcription, indicating Tet2 preserves genome integrity via ribosome biogenesis-independent mechanisms.”

2.Details of transient knockdown experiments: In the Methods section, please specify the cell state (including passage number and proliferation status) after 48 hours of doxycycline treatment to better exclude potential long-term adaptive effects.

Response: We thank the reviewer for this suggestion. In the Methods section, we have added the following description of the cell state during the 48-hour doxycycline treatment (page 29, lines 620–626):

“For transient knockdown experiments with the doxycycline-inducible system, ESCs were cultured in the presence of 1 μg/mL doxycycline for 48 hours. Cells were cultured at approximately passage 20 and kept in logarithmic growth throughout treatment. Cells were plated at optimized seeding density to achieve 60%–70% confluence at the 48 h endpoint, with no intermediate passaging performed. The short 48-hour induction minimizes long-term cellular adaptive responses. qRT-PCR was used to verify gene knockdown efficiency.”

3.Discussion of rDNA binding pattern: Tcf15 binds to the coding regions (18S, 28S) rather than the promoter region of rDNA, which represents an unusual binding pattern. Please briefly comment in the Discussion on the potential functional significance of this binding mode—for example, whether it participates in transcription elongation or maintains open chromatin state across the coding region.

Response: We thank the reviewer for this suggestion. We have discussed this point in the Discussion section (page 26, lines 543–551)：

“An intriguing observation from our study is that Tcf15 binds preferentially to the coding regions (18S and 28S) rather than the promoter of rRNA genes. This unusual binding pattern raises the possibility that Tcf15 may facilitate transcription elongation or maintain an open chromatin configuration across the entire rDNA coding region, rather than merely initiating transcription. Supporting this notion, our ChIP-seq and CUT&Tag data showed that Tcf15 occupancy correlates with H3K4me3 enrichment and reduced H3K27me3 deposition across the coding regions. Whether Tcf15 directly associates with the elongating RNA polymerase I complex or functions to preserve the open chromatin state during active transcription represents an open question for future investigation.”

4.Mechanistic link between MCM proteins and replication stress: More explicitly establish the direct mechanistic connection in the main text between reduced MCM protein levels, decreased DNA replication origin density, and the establishment of replication stress.

Response: We thank the reviewer for this suggestion. In the Results section (page 22–23, lines 470–476), we have expanded the description of the MCM-related phenotype as follows:

“This phenotype coincided with decreased expression of MCM proteins (Mcm2, Mcm3, Mcm6) (PMID: **28732205**), which form the core of the replicative helicase complex essential for replication origin licensing and fork progression. Reduced MCM levels result in a diminished pool of licensed origins, thereby decreasing origin density (Fig 6H). This origin scarcity impairs the cell's ability to efficiently complete DNA replication, particularly under replicative stress, leading to fork stalling, ATR-Chk1 signaling defects (Fig 6F), and ultimately DNA damage (PMID: **26190528**).”
