# Supplementary material for: Tcf15 promotes an open nucleolar chromatin state to safeguard ribosome biogenesis and genome stability in mouse embryonic stem cells
Source: PLoS Biol. 2026 Aug 25;24(8):e3003954. doi: 10.1371/journal.pbio.3003954 (PMC13505954; doi:10.1371/journal.pbio.3003954)
Supplement: Peer review history

# Editor report: Decision Letter 0

**DOI:** 10.1371/journal.pbio.3003954.r001

**Authors:** Richard Hodge

22 Jan 2026

Dear Dr Zheng,

Thank you for submitting your manuscript entitled "Tcf15 promotes an open nucleolar chromatin state to safeguard ribosome biogenesis and genome stability in mouse embryonic stem cells" for consideration as a Research Article by PLOS Biology.

Your manuscript has now been evaluated by the PLOS Biology editorial staff, as well as by an academic editor with relevant expertise, and I am writing to let you know that we would like to send your submission out for external peer review.

However, before we can send your manuscript to reviewers, we need you to complete your submission by providing the metadata that is required for full assessment. To this end, please login to Editorial Manager where you will find the paper in the 'Submissions Needing Revisions' folder on your homepage. Please click 'Revise Submission' from the Action Links and complete all additional questions in the submission questionnaire.

Once your full submission is complete, your paper will undergo a series of checks in preparation for peer review. After your manuscript has passed the checks it will be sent out for review. To provide the metadata for your submission, please Login to Editorial Manager (https://www.editorialmanager.com/pbiology) within two working days, i.e. by Jan 24 2026 11:59PM.

If your manuscript has been previously peer-reviewed at another journal, PLOS Biology is willing to work with those reviews in order to avoid re-starting the process. Submission of the previous reviews is entirely optional and our ability to use them effectively will depend on the willingness of the previous journal to confirm the content of the reports and share the reviewer identities. Please note that we reserve the right to invite additional reviewers if we consider that additional/independent reviewers are needed, although we aim to avoid this as far as possible. In our experience, working with previous reviews does save time.

If you would like us to consider previous reviewer reports, please edit your cover letter to let us know and include the name of the journal where the work was previously considered and the manuscript ID it was given. In addition, please upload a response to the reviews as a 'Prior Peer Review' file type, which should include the reports in full and a point-by-point reply detailing how you have or plan to address the reviewers' concerns.

During the process of completing your manuscript submission, you will be invited to opt-in to posting your pre-review manuscript as a bioRxiv preprint. Visit http://journals.plos.org/plosbiology/s/preprints for full details. If you consent to posting your current manuscript as a preprint, please upload a single Preprint PDF.

Feel free to email us at plosbiology@plos.org if you have any queries relating to your submission.

Kind regards,

Richard

Richard Hodge, PhD

Senior Editor, PLOS Biology

rhodge@plos.org

PLOS

Empowering researchers to transform science

Carlyle House, Carlyle Road, Cambridge, CB4 3DN, United Kingdom

California (U.S.) corporation #C2354500, based in San Francisco

# Peer review: Decision Letter 1

**DOI:** 10.1371/journal.pbio.3003954.r002

**Authors:** Richard Hodge

17 Mar 2026

Dear Dr Zheng,

Thank you for your patience while your manuscript "Tcf15 promotes an open nucleolar chromatin state to safeguard ribosome biogenesis and genome stability in mouse embryonic stem cells" was peer-reviewed at PLOS Biology as a Research Article. Please accept my sincere apologies for the delays that you have experienced during the peer review process. Your manuscript has now been evaluated by the PLOS Biology editors, an Academic Editor with relevant expertise, and by three independent reviewers.

In light of the reviews, which you will find at the end of this email, we would like to invite you to revise the work to thoroughly address the reviewers' reports.

As you will see, the reviewers are generally positive about your manuscript and think it is interesting and well done. However, they note that additional experiments are required to fully support the conclusions. Specifically, Reviewer #1 requests that additional validation for Tcf15 binding to rDNA is provided using rDNA-FISH, as well as RNA half-life experiments using Actinomycin D. However, after discussions with the academic editor, we will not make these two experimental requests essential for the revision. Reviewer #2 raises concerns that the data is too correlative at this stage to support the mechanistic claims and requests several experiments that we agree would strengthen the work. This includes the addition of causal rescue experiments to show that genome instability results from defective ribosome biogenesis mediated by Tcf15. In addition, the reviewer raises concerns that the manuscript does exclude broader chromatin roles for Tcf15 and does not directly demonstrate the recruitment of Tet2 and Rbbp5. Finally, Reviewer #3 is broadly positive about the study and provides a few minor suggestions.

Given the extent of revision needed, we cannot make a decision about publication until we have seen the revised manuscript and your response to the reviewers' comments. Your revised manuscript is likely to be sent for further evaluation by all or a subset of the reviewers.

In addition to these revisions, you will need to complete some formatting changes, which you will receive in a follow up email. A member of our team will be in touch with a set of requests shortly.

We expect to receive your revised manuscript within 3 months. Please email us (plosbiology@plos.org) if you have any questions or concerns, or would like to request an extension.

At this stage, your manuscript remains formally under active consideration at our journal; please notify us by email if you do not intend to submit a revision so that we may withdraw it.

**IMPORTANT - SUBMITTING YOUR REVISION**

Your revisions should address the specific points made by each reviewer. Please submit the following files along with your revised manuscript:

1. A 'Response to Reviewers' file - this should detail your responses to the editorial requests, present a point-by-point response to all of the reviewers' comments, and indicate the changes made to the manuscript.

*NOTE: In your point-by-point response to the reviewers, please provide the full context of each review. Do not selectively quote paragraphs or sentences to reply to. The entire set of reviewer comments should be present in full and each specific point should be responded to individually, point by point.

You should also cite any additional relevant literature that has been published since the original submission and mention any additional citations in your response.

2. In addition to a clean copy of the manuscript, please also upload a 'track-changes' version of your manuscript that specifies the edits made. This should be uploaded as a "Revised Article with Changes Highlighted" file type.

*Re-submission Checklist*

When you are ready to resubmit your revised manuscript, please refer to this re-submission checklist: https://plos.io/Biology_Checklist

To submit a revised version of your manuscript, please go to https://www.editorialmanager.com/pbiology/ and log in as an Author. Click the link labelled 'Submissions Needing Revision' where you will find your submission record.

Please make sure to read the following important policies and guidelines while preparing your revision:

*Published Peer Review*

Please note while forming your response, if your article is accepted, you may have the opportunity to make the peer review history publicly available. The record will include editor decision letters (with reviews) and your responses to reviewer comments. If eligible, we will contact you to opt in or out. Please see here for more details:

https://blogs.plos.org/plos/2019/05/plos-journals-now-open-for-published-peer-review/

*PLOS Data Policy*

Please note that as a condition of publication PLOS' data policy (http://journals.plos.org/plosbiology/s/data-availability) requires that you make available all data used to draw the conclusions arrived at in your manuscript. If you have not already done so, you must include any data used in your manuscript either in appropriate repositories, within the body of the manuscript, or as supporting information (N.B. this includes any numerical values that were used to generate graphs, histograms etc.). For an example see here: http://www.plosbiology.org/article/info%3Adoi%2F10.1371%2Fjournal.pbio.1001908#s5

*Blot and Gel Data Policy*

We require the original, uncropped and minimally adjusted images supporting all blot and gel results reported in an article's figures or Supporting Information files. We will require these files before a manuscript can be accepted so please prepare them now, if you have not already uploaded them. Please carefully read our guidelines for how to prepare and upload this data: https://journals.plos.org/plosbiology/s/figures#loc-blot-and-gel-reporting-requirements

*Protocols deposition*

To enhance the reproducibility of your results, we recommend that if applicable you deposit your laboratory protocols in protocols.io, where a protocol can be assigned its own identifier (DOI) such that it can be cited independently in the future. Additionally, PLOS ONE offers an option for publishing peer-reviewed Lab Protocol articles, which describe protocols hosted on protocols.io. Read more information on sharing protocols at https://plos.org/protocols?utm_medium=editorial-email&utm_source=authorletters&utm_campaign=protocols

Thank you again for your submission to our journal. We hope that our editorial process has been constructive thus far, and we welcome your feedback at any time. Please don't hesitate to contact us if you have any questions or comments.

Best regards,

Richard

Richard Hodge, PhD

Senior Editor, PLOS Biology

rhodge@plos.org

------------------------------------

REVIEWS:

Reviewer #1 (Lei Lei, identifies herself): This manuscript by Dong et al. investigates the role of the transcription factor Tcf15 in maintaining an open nucleolar chromatin state in embryonic stem cells (ESCs). The authors report that Tcf15 localizes to the nucleolus, binds to the coding regions of rRNA genes, and recruits the epigenetic regulators Tet2 and Rbbp5. Through these interactions, Tcf15 suppresses DNA methylation and H3K27me3 modification while promoting H3K4me3 modification at rDNA loci. Loss of Tcf15 impairs ribosome biogenesis (RiBi), selectively compromises the translation of genome-maintenance factors, and leads to marked genome instability. Overall, this study identifies an underexplored function of Tcf15: sustaining a hyperactive chromatin state at rDNA loci to support robust ribosome biogenesis in ESCs. These findings are of significant value for advancing our understanding of nucleolar function and rRNA gene regulation in embryonic stem cells.

However, several aspects related to experimental design, data interpretation, and manuscript structure warrant improvement. Specific comments are as follows:

Major:

1. Verification of Tcf15 binding to rDNA (Result 1)

The authors employed immunofluorescence and nucleocytoplasmic separation to determine the nucleolar localization of Tcf15, and provided ChIP-seq and ChIP-qPCR data to demonstrate Tcf15 binding to rDNA. However, it is recommended to supplement with co-localization experiments using rDNA-FISH combined with Tcf15 immunofluorescence to more intuitively and comprehensively demonstrate the spatial binding relationship between Tcf15 and rDNA in the nucleolus, thereby strengthening the persuasiveness of the conclusions.

2. Verification of rRNA stability (Result 4A)

Result 4A shows decreased rRNA levels, but this phenotype may arise from two mechanisms: first, reduced rRNA transcription; second, increased degradation of 47S pre-rRNA. To exclude the influence of the latter mechanism, it is recommended to perform RNA half-life experiments using Actinomycin D (ActD) to clarify whether the decrease in rRNA is due to transcriptional inhibition, thereby interpreting the data more accurately.

3. Questions regarding nascent protein localization (Result 4C)

Protein synthesis typically occurs primarily in the cytoplasm; however, Result 4C shows that nascent proteins are more enriched at the nucleolus. This phenomenon differs from classical understanding, and the authors are advised to explain this: Does this reflect specific translational regulatory mechanisms within the nucleolus? Or is it due to the particularity of experimental conditions? Adding relevant discussion will help readers understand the biological significance of this result.

4. Supplementary experiments on the impact of Tcf15 on ESC pluripotency

This study focuses on the molecular function of Tcf15 in the nucleolus, revealing its effects on rRNA gene epigenetic regulation and ribosome biogenesis. Considering that ribosome biogenesis and nucleolar function are closely related to embryonic stem cell self-renewal and differentiation potential, the authors are recommended to supplement experiments on the effects of Tcf15 knockdown on mouse embryonic stem cell pluripotency maintenance and differentiation capacity, such as AP staining, pluripotential markers expression and EB formation, etc.

5. Supplementary verification of the Tcf15-Tet2/Rbbp5 signaling pathway

This manuscript describes the interaction between Tcf15 and Tet2/Rbbp5 and their effects on rRNA transcription and ribosome biogenesis. To further complete the interpretation of this signaling pathway, the following experiments are recommended:

1) Detect the effects of manipulating Tet2 or Rbbp5 (such as knockdown/overexpression) on rRNA transcription and ribosome biogenesis.

2) Compare whether the phenotypes after manipulating Tet2/Rbbp5 are consistent with those after manipulating Tcf15.

If the results are consistent, it would more strongly support the molecular mechanism that "Tcf15 regulates nucleolar function through Tet2/Rbbp5," enhancing the logical completeness of the manuscript.

6. Optimization of the transition between Result 6 and preceding sections

The connection between Result 6 and the preceding sections is not sufficiently smooth. The preceding sections mainly describe Tcf15's effects on rDNA epigenetic regulation, rRNA transcription, and ribosome biogenesis—these factors all significantly influence nucleolar activity, while the nucleolus is a key factor in maintaining genome stability.

It is recommended that the authors add transitional content before Result 6: explicitly stating that Tcf15 may affect genome stability by regulating nucleolar structure and rRNA production, before then elaborating on the specific results of Tcf15's effects on genome stability (such as DNA damage, chromosomal instability, etc.). This would make the manuscript logic smoother and help readers understand the inferential chain from nucleolar function to genome stability.

Minor:

1. In Figure 3, the authors only assess co-localization between Tet2 and Tcf15. To strengthen the proposed recruitment model and maintain internal consistency, the authors should also perform immunofluorescence analyses to examine co-localization between Rbbp5 and Tcf15.

2. Statistical reporting is generally sound, but the number of biological replicates should be stated consistently across all figures.

3. Terminology and Abbreviation Standardization Issues

1) ESCs was not defined as "Embryonic Stem Cells" upon first appearance. It is recommended to add the full name.

2) The abbreviations RiBi (ribosome biogenesis) and rDNA (ribosomal DNA) appear multiple times throughout the manuscript.

3) Several abbreviations appear at inappropriate positions for their first usage. For example, rRNA abbreviation first appears in the Abstract rather than the Introduction section. The first appearance of pre-rRNAs abbreviation is also inappropriate. A thorough self-check of the entire manuscript is recommended.

It is suggested to adjust the first appearance positions of these abbreviations to conform to academic standards, providing the full expression when first used in the Introduction.

Reviewer #2: This study proposes that Tcf15 is an ESC-specific regulator of rDNA chromatin state that maintains ribosome biogenesis (RiBi) and genome stability. The authors report that Tcf15 localizes to the nucleolus, recruits Tet2 and Rbbp5 to rRNA genes, prevents repressive chromatin formation, sustains rRNA transcription, and thereby preserves translation of DNA replication/repair factors and genome integrity. The topic is interesting because regulation of rDNA chromatin in pluripotent stem cells is important and incompletely understood. The experiments are extensive and technically diverse (ChIP-seq, CUT&Tag, WGBS, Ribo-seq, ribosome profiling, and DNA damage assays). However, the central mechanistic claim is currently not convincingly supported by causality, and several conclusions substantially overreach the presented data. In its current form, the manuscript reads more as a correlation chain: from Tcf15 KD to rDNA chromatin changes, reduced rRNA, translation changes, and to genome instability. But the authors have not demonstrated that these events are mechanistically connected rather than parallel consequences of Tcf15 perturbation. Major conceptual and technical clarifications are required before publication in PLOS Biology.

1.The main claim is that genome instability results from defective ribosome biogenesis mediated by Tcf15. However, the evidence only shows association. But no causal rescue experiment connects these steps.

At present, Tcf15 could independently regulate nucleolar chromatin, replication programs, stress responses rather than acting through ribosome biogenesis.

2.The manuscript emphasizes a nucleolus-specific function of TCF15, but the data do not exclude broader chromatin roles. My concerns are 1) ChIP-seq binding outside rDNA is not analyzed. 2) Tet2 and Rbbp5 are global chromatin regulators. 3) Only rDNA effects are investigated.

The reviewer therefore cannot conclude Tcf15 is an rDNA-specific regulator rather than a general transcription or chromatin factor with a visible nucleolar pool.

3. Recruitment of Tet2 and Rbbp5 is not demonstrated (interaction ≠ recruitment)

The authors propose Tcf15 recruits Tet2 and Rbbp5 to rRNA genes by showing co-IP interaction and nucleolar co-localization (Tet2 only). This is a critical mechanistic gap. Without showing loss of Tet2/Rbbp5 binding at rDNA, the recruitment model is speculative.

4. The H3K27me3 interpretation is incomplete

A striking phenotype is increased H3K27me3 at rDNA. The authors interpret this as a consequence of reduced H3K4me3 antagonism. Crucially, the authors should determine whether known H3K27me3 regulators (e.g., nucleolar PRC2 localization mechanisms) are altered. Without this, the chromatin model is incomplete.

5. The translation phenotype is internally inconsistent

The study reports severe reduction in rRNA transcription but nearly equal numbers of translationally up- and down-regulated genes. This contradicts a simple ribosome shortage model.

An alternative explanation should be addressed because selective translation changes are a hallmark of stress signaling, not merely reduced ribosome abundance.

6. Over-interpretation of genome instability

The manuscript concludes Tcf15 "safeguards genome stability."

However, Tcf15 may directly regulates replication genes rather than through ribosome biogenesis.

Therefore the claim should be softened or mechanistically tested.

Minor concerns

1.Localization of Rbbp5 in the nucleolus is not shown by IF.

2.Tet2/Rbbp5 localization after Tcf15 KD is missing.

3.Only knockdown is used; acute degradation would be more convincing.

4.Potential overexpression artifacts from Flag-Tcf15 remain a concern.

5.The manuscript does not examine pluripotency state changes.

Reviewer #3: This study identifies Tcf15 as a regulator of rDNA chromatin state and ribosome biogenesis in embryonic stem cells (ESCs). The authors show that Tcf15 localizes to the nucleolus, binds rRNA gene regions, and recruits epigenetic modifiers such as Tet2 and the H3K4 methyltransferase component Rbbp5 to maintain an active chromatin configuration at rDNA loci. Loss of Tcf15 increases repressive chromatin marks, reduces pre-rRNA transcription, and impairs ribosome biogenesis, leading to altered translation of mRNAs involved in DNA replication and repair. These defects ultimately cause genomic instability, revealing a previously unrecognized link between rDNA chromatin regulation, ribosome production, and genome maintenance in ESCs.

Overall, this is a really nice study and I only have minor suggestions:

- Fig. 3A - it is not clear where tet2. Rbbp5, and Tcf15 are on the plot. Also, some dots look orange. Is that on purpose and what does it signify?

- Fig. 4D - can the authors put the flow cytometry data on log scale instead of semi-log and normalize the y-axis to %max.

- Fig. 4D - Can the authors comment on why they have fewer cells for the knockdowns?

# Author comment: Author response to Decision Letter 2

**DOI:** 10.1371/journal.pbio.3003954.r003

30 Jun 2026

Attachment Submitted filename: point to point response_letter_rm_editor.docx

# Peer review: Decision Letter 2

**DOI:** 10.1371/journal.pbio.3003954.r004

**Authors:** Richard Hodge

23 Jul 2026

Dear Dr Zheng,

Thank you for your patience while we considered your revised manuscript "Tcf15 promotes an open nucleolar chromatin state to safeguard ribosome biogenesis and genome stability in mouse embryonic stem cells" for publication as a Research Article at PLOS Biology. This revised version of your manuscript has been evaluated by the PLOS Biology editors, the Academic Editor and two of the original reviewers.

Based on the reviews, I am pleased to say that we are likely to accept this manuscript for publication, provided you satisfactorily address the remaining points raised by Reviewer #1. In addition, please make sure to address the following data and other policy-related requests that I have provided below (A-F):

(A) In the animal ethics statement in the Methods section, please provide the specific approval number issued by the Institutional Animal Care and Use Committee to conduct the study.

(B) Please also ensure that each of the relevant figure legends in your manuscript include information on *WHERE THE UNDERLYING DATA CAN BE FOUND*, and ensure your supplemental data file/s has a legend.

(C) Thank you for providing the sequencing data in the National Genomics Data Center database (CRA029710). However, we note that the data is currently on hold for release. We ask that you please make the structures publicly available at this stage before publication. Please also ensure that the mass spectrometry data deposited in the OMIX database is made publicly available.

(D) Per journal policy, if you have generated any custom code during the course of this investigation, please make it available without restrictions. Please ensure that the code is sufficiently well documented and reusable, and that your Data Statement in the Editorial Manager submission system accurately describes where your code can be found. More information on our Code Policy, what and how to share can be found here: https://journals.plos.org/plosbiology/s/code-availability

Please note that we cannot accept sole deposition of code in GitHub, as this could be changed after publication. However, you can archive this version of your publicly available GitHub code to Zenodo. Once you do this, it will generate a DOI number, which you will need to provide in the Data Accessibility Statement (you are welcome to also provide the GitHub access information). See the process for doing this here: https://docs.github.com/en/repositories/archiving-a-github-repository/referencing-and-citing-content

(E) Please ensure that you are using best practice for statistical reporting and data presentation. These are our guidelines https://journals.plos.org/plosbiology/s/best-practices-in-research-reporting#loc-statistical-reporting and a useful resource on data presentation https://journals.plos.org/plosbiology/article?id=10.1371/journal.pbio.1002128

- If you are reporting experiments where n ≤ 5, please plot each individual data point.

(F) Please note that per journal policy, the model system/species studied should be clearly stated in the abstract of your manuscript.

------------------------------------------------------------------------

As you address these items, please take this last chance to review your reference list to ensure that it is complete and correct. If you have cited papers that have been retracted, please include the rationale for doing so in the manuscript text, or remove these references and replace them with relevant current references. Any changes to the reference list should be mentioned in the cover letter that accompanies your revised manuscript.

In addition to these revisions, you may need to complete some formatting changes, which you will receive in a follow up email. A member of our team will be in touch with a set of requests shortly. If you do not receive a separate email within a few days, please assume that checks have been completed, and no additional changes are required.

We expect to receive your revised manuscript within two weeks.

To submit your revision, please go to https://www.editorialmanager.com/pbiology/ and log in as an Author. Click the link labelled 'Submissions Needing Revision' to find your submission record. Your revised submission must include the following:

- a cover letter that should detail your responses to any editorial requests, if applicable, and whether changes have been made to the reference list

- a Response to Reviewers file that provides a detailed response to the reviewers' comments (if applicable, if not applicable please do not delete your existing 'Response to Reviewers' file.)

- a track-changes file indicating any changes that you have made to the manuscript.

NOTE: If Supporting Information files are included with your article, note that these are not copyedited and will be published as they are submitted. Please ensure that these files are legible and of high quality (at least 300 dpi) in an easily accessible file format. For this reason, please be aware that any references listed in an SI file will not be indexed. For more information, see our Supporting Information guidelines:

https://journals.plos.org/plosbiology/s/supporting-information

*Published Peer Review History*

Please note that you may have the opportunity to make the peer review history publicly available. The record will include editor decision letters (with reviews) and your responses to reviewer comments. If eligible, we will contact you to opt in or out. Please see here for more details:

https://plos.org/published-peer-review-history/

*Press*

Should you, your institution's press office or the journal office choose to press release your paper, please ensure you have opted out of Early Article Posting on the submission form. We ask that you notify us as soon as possible if you or your institution is planning to press release the article.

*Protocols deposition*

To enhance the reproducibility of your results, we recommend that if applicable you deposit your laboratory protocols in protocols.io, where a protocol can be assigned its own identifier (DOI) such that it can be cited independently in the future. Additionally, PLOS ONE offers an option for publishing peer-reviewed Lab Protocol articles, which describe protocols hosted on protocols.io. Read more information on sharing protocols at https://plos.org/protocols?utm_medium=editorial-email&utm_source=authorletters&utm_campaign=protocols

Please do not hesitate to contact me should you have any questions.

Best wishes,

Richard

Richard Hodge, PhD

Senior Editor, PLOS Biology

rhodge@plos.org

-----------------------------------------------------------------------

Reviewer remarks:

Reviewer #1: Through extensive and rigorous additional experiments, the authors have addressed the major mechanistic concerns raised in the first round of review. This study identifies Tcf15 as a novel regulator of rDNA chromatin state in stem cells and reveals a previously unrecognized link between ribosome biogenesis and genome stability, which is of significant scientific importance.

1.Discussion of Tet2 phenotype: The authors should briefly mention in the Discussion section the observation that Tet2 knockdown also causes DNA damage despite having no effect on rRNA transcription, and note that Tet2 may maintain genome stability through pathways independent of ribosome biogenesis.

2.Details of transient knockdown experiments: In the Methods section, please specify the cell state (including passage number and proliferation status) after 48 hours of doxycycline treatment to better exclude potential long-term adaptive effects.

3.Discussion of rDNA binding pattern: Tcf15 binds to the coding regions (18S, 28S) rather than the promoter region of rDNA, which represents an unusual binding pattern. Please briefly comment in the Discussion on the potential functional significance of this binding mode—for example, whether it participates in transcription elongation or maintains open chromatin state across the coding region.

4.Mechanistic link between MCM proteins and replication stress: More explicitly establish the direct mechanistic connection in the main text between reduced MCM protein levels, decreased DNA replication origin density, and the establishment of replication stress.

Reviewer #2 (Xiaohua Shen, identifies herself): The authors have satisfactorily addressed my previous concerns. The additional experiments and analyses have significantly strengthened the mechanistic model, particularly regarding Tcf15 specificity at rDNA loci, recruitment of Tet2/Rbbp5, the role of the Tcf15-Rbbp5 axis in ribosome biogenesis, and the connection to genome stability. The remaining limitations are appropriately discussed in the revised manuscript and do not compromise the main conclusions. I therefore consider the manuscript suitable for publication.

# Author comment: Author response to Decision Letter 3

**DOI:** 10.1371/journal.pbio.3003954.r005

30 Jul 2026

Attachment Submitted filename: 2026.7.29 PloS biology review comments-revise-dyp-wl(1).docx

# Editor report: Decision Letter 3

**DOI:** 10.1371/journal.pbio.3003954.r006

**Authors:** Richard Hodge

3 Aug 2026

Dear Dr Zheng,

On behalf of my colleagues and the Academic Editor, Christa Buecker, I am pleased to say that we can accept your manuscript for publication, provided you address any remaining formatting and reporting issues. These will be detailed in an email you should receive within 2-3 business days from our colleagues in the journal operations team; no action is required from you until then. Please note that we will not be able to formally accept your manuscript and schedule it for publication until you have completed any requested changes.

Please take a minute to log into Editorial Manager at http://www.editorialmanager.com/pbiology/, click the "Update My Information" link at the top of the page, and update your user information to ensure an efficient production process.

PRESS

We frequently collaborate with press offices. If your institution or institutions have a press office, please notify them about your upcoming paper at this point, to enable them to help maximise its impact. If the press office is planning to promote your findings, we would be grateful if they could coordinate with biologypress@plos.org. If you have previously opted in to the early version process, we ask that you notify us immediately of any press plans so that we may opt out on your behalf.

We also ask that you take this opportunity to read our Embargo Policy regarding the discussion, promotion and media coverage of work that is yet to be published by PLOS. As your manuscript is not yet published, it is bound by the conditions of our Embargo Policy. Please be aware that this policy is in place both to ensure that any press coverage of your article is fully substantiated and to provide a direct link between such coverage and the published work. For full details of our Embargo Policy, please visit http://www.plos.org/about/media-inquiries/embargo-policy/.

Thank you again for choosing PLOS Biology for publication and supporting Open Access publishing. We look forward to publishing your study.

Best wishes,

Richard

Richard Hodge, PhD

Senior Editor, PLOS Biology

rhodge@plos.org

PLOS

Empowering researchers to transform science

Carlyle House, Carlyle Road, Cambridge, CB4 3DN, United Kingdom

California (U.S.) corporation #C2354500, based in San Francisco
